# Distinct Evolutionary Trajectories of Major Intracellular Proteolytic Systems in Gilthead Seabream and European Seabass

**DOI:** 10.3390/biology15191755

**Published:** 2026-10-02

**Authors:** Rafael Angelakopoulos, Andreas Tsipourlianos, Themistoklis Giannoulis, Katerina A. Moutou

**Affiliations:** 1 Laboratory of Genetics, Comparative and Evolutionary Biology, Department of Biochemistry and Biotechnology, University of Thessaly, 41500 Larissa, Greece; antsipou@uth.gr (A.T.); kmoutou@uth.gr (K.A.M.); 2 Laboratory of Biology, Genetics and Bioinformatics, Department of Animal Science, University of Thessaly, 41334 Larissa, Greece; thgianno@uth.gr

**Keywords:** proteolysis, teleost evolution, whole-genome duplication, regulatory divergence, gilthead seabream, European seabass

## Abstract

Intracellular proteolysis is essential for protein turnover, cellular homeostasis, stress responses, immune function, development and tissue remodeling. In teleost fishes, ancient vertebrate and teleost-specific whole-genome duplication events (1R, 2R, Ts3R) increased gene copy number, followed by lineage-specific patterns of gene retention, loss and regulatory divergence. We investigated three major intracellular proteolytic systems, the ubiquitin–proteasome system (UPS), calpains and cathepsins, in gilthead seabream (*Sparus aurata*) and European seabass (*Dicentrarchus labrax*). Using comparative genomic, phylogenetic, syntenic and transcriptomic approaches, we identified and curated gene repertoires, inferred duplication histories and examined developmental and tissue-specific expression patterns. We identified 29 and 22 calpain genes, 23 and 17 cathepsin genes and 61 and 53 UPS genes in gilthead seabream and European seabass, respectively. Phylogenetic and synteny analyses indicated that retained paralogs were inferred to derive from a combination of ancient vertebrate duplications, teleost-specific whole-genome duplication and lineage- or species-specific events. Single copy genes predominated the UPS and only selected duplicated components showed patterns compatible with retention following the Ts3R. In contrast, calpains and cathepsins showed broader paralog retention, mixed duplication origins and greater diversity in developmental and tissue-specific expression patterns. Expression profiling indicated that most UPS genes were broadly expressed, whereas several calpain and cathepsin paralogs displayed stage- or tissue-dependent expression patterns. Overall, these results suggest that intracellular proteolytic systems in teleosts followed partly distinct evolutionary trajectories, with comparatively greater conservation of the multi-subunit UPS and expression patterns consistent with greater regulatory divergence among calpain and cathepsin paralogs. Retained proteolytic paralogs therefore displayed divergent expression patterns consistent with possible regulatory divergence which could reflect subfunctionalization following genome duplication.

## Simple Summary

Cells continuously remove damaged or unnecessary proteins to function properly. This process is controlled by several protein-degradation systems. Teleost fish experienced an additional, whole genome duplication event known as the teleost-specific whole-genome duplication (Ts3R), which created extra copies of many genes; some copies were subsequently lost, whereas others may have undergone functional or regulatory divergence. We studied three major proteolytic systems; the ubiquitin–proteasome system, calpains and cathepsins in gilthead seabream and European seabass. We compared their genes and evolutionary relationships and examined their expression during development and across adult tissues. Most ubiquitin–proteasome system genes were present as single copies and broadly expressed, whereas calpains and cathepsins contained more duplicated genes, several of which displayed distinct expression patterns among developmental stages or tissues. These findings indicate that the three systems followed different evolutionary trajectories and identify duplicated genes with distinct expression patterns that may be associated with regulatory divergence in teleost fishes.

## 1. Introduction

Proteolysis is a fundamental biological process that operates across tissues and developmental stages and is essential for cellular homeostasis. At the molecular level, proteolysis involves the enzymatic hydrolysis of peptide bonds, resulting either in the degradation of proteins into smaller peptides and amino acids or in limited proteolytic processing that modifies protein function, localization, stability, or interaction capacity. Thus, proteolysis is not only a degradative mechanism, but also a regulatory process that contributes to protein turnover, protein quality control, stress responses, immune function, development, and tissue remodeling [1,2,3]. In fishes, intracellular proteolytic control is particularly relevant because growth, muscle remodeling, immune responses, stress adaptation, and developmental transitions require continuous and tightly regulated protein remodeling [4,5,6,7,8,9].

Among intracellular proteolytic systems, the ubiquitin–proteasome system (UPS), calpains, and cathepsins represent three major but mechanistically distinct components of proteostasis. In the UPS, E1 ubiquitin-activating, E2 ubiquitin-conjugating, and E3 ubiquitin-ligating enzymes mediates selective ubiquitination of target proteins which can subsequently be degraded by the ATP-dependent 26S proteasome and regulates essential cellular processes, including cell-cycle progression, stress responses, and immune regulation [10,11,12]. This multi-subunit machinery requires coordinated assembly of its catalytic core and regulatory particles. Calpains, in contrast, are calcium-dependent cysteine proteases that commonly, although not exclusively, act through limited proteolytic processing rather than complete substrate degradation. Through this mode of action, they regulate cytoskeletal remodeling, signal transduction, membrane dynamics, apoptosis, and cellular reorganization [13,14]. Cathepsins are predominantly lysosomal proteases involved in intracellular protein turnover, autophagy, antigen processing, and, in specific contexts, some cathepsins can participate in cytosolic or extracellular proteolysis [15]. Together, these systems form distinct but interacting modules of intracellular proteolysis [16]. Experimental evidence indicates that they can function cooperatively during intracellular protein degradation. Calpains, the proteasome, and lysosomal proteases have been shown to act in tandem during degradation of cytoskeletal and sarcomeric proteins, while a conserved calpain–cathepsin cascade further demonstrates functional crosstalk between cytosolic and lysosomal proteolytic pathways [17,18].

Whole-genome duplication (WGD) events have contributed substantially to the evolution of vertebrate gene families. Vertebrates experienced two ancient rounds of WGD (1R and 2R), while teleost fishes experienced an additional teleost-specific WGD (Ts3R). This event has been dated to approximately 350–320 million years [19] whereas a recent molecular-dating analysis estimated an early–middle Permian interval of 290–265 million years ago [20]. These events provided genetic material that could subsequently be retained, lost, or repurposed during evolution; following duplication, one copy may be lost through nonfunctionalization, both copies may retain similar functions, ancestral functions may be partitioned between paralogs through subfunctionalization, or one copy may acquire a new function through neofunctionalization [21,22,23,24]. Therefore, the evolutionary fate of duplicated genes depends on the interaction between redundancy, natural selection, gene dosage, molecular function, and network context.

Teleost fishes provide an especially suitable framework for studying this process. They represent the most species-rich vertebrate lineage and exhibit remarkable morphological, ecological, physiological, and developmental diversity [25,26]. Their evolutionary history includes the additional 3R-WGD, followed by extensive lineage-specific gene loss, retention, and diversification. As a result, teleost genomes provide an opportunity to examine how duplicated genes were filtered after genome duplication and whether retained paralogs diverged in genomic origin, protein structure, developmental expression, or tissue-specific regulation [27,28,29].

This question is especially relevant for proteolytic systems, because protease function must be controlled spatially and temporally, while the three systems differ substantially in molecular architecture and regulatory organization [3,14,15,30]. The UPS depends on the coordinated activity of multiple core, regulatory, and accessory components, whereas calpains and cathepsins comprise individual proteases whose activities are controlled through distinct regulatory mechanisms and cellular contexts. Consequently, duplication of a component belonging to a highly coordinated multi-protein system may be subject to different constraints from duplication of an individual protease, for which changes in expression or regulation may be accommodated more readily. These contrasting levels of molecular organization therefore provide a conceptual basis for asking whether the three proteolytic systems differ in the extent to which duplicated genes are retained and transcriptionally diversified. Although individual calpain, cathepsin and UPS components have been characterized in teleosts and their expression has been investigated in particular tissues, developmental stages, or experimental contexts [31,32,33,34,35,36,37,38], comparative work examining duplication history, paralog retention and expression divergence across multiple intracellular proteolytic systems within the same teleost species remains limited. It is therefore unclear whether proteolytic systems with different molecular architectures show distinct patterns of gene retention and expression diversification after genome duplication [31,32,33,34,35,36,37,38].

In this study, we examined whether major intracellular proteolytic systems in teleosts followed comparable or system-specific evolutionary trajectories after vertebrate and teleost genome duplications. Specifically, we asked whether differences in molecular architecture and regulatory organization among the ubiquitin–proteasome system, calpains, and cathepsins are associated with distinct patterns of paralog retention, duplication origin, and expression divergence. We addressed this question in gilthead seabream (*Sparus aurata*) and European seabass (*Dicentrarchus labrax*) by integrating phylogenetic analyses to infer gene relationships and duplication histories, syntenic analyses to support the genomic origin of retained paralogs, and developmental and tissue-specific expression profiling to evaluate regulatory differentiation among gene copies. Both species are important in Mediterranean aquaculture and have available multiple genomic and transcriptomic resources that allow comparative analysis across developmental stages and adult tissues [39,40,41,42]. This comparative framework allowed us to assess whether retained proteolytic paralogs are characterized by broadly consistent expression patterns or by developmental and tissue-specific expression differences consistent with possible regulatory divergence. By comparing a multi-subunit proteolytic complex with two protease families, we aimed to assess whether differences in molecular organization are associated with distinct patterns of paralog retention and diversification after genome duplication.

## 2. Materials and Methods

### 2.1. Comparative Genomic and Phylogenetic Analysis

#### 2.1.1. Database Mining and Sequence Retrieval

A comparative database-mining approach was used to identify genes belonging to the UPS, calpains, and cathepsins. Human gene family members were first retrieved from the HUGO Gene Nomenclature Committee database (HGNC, https://www.genenames.org, accessed on 26 February 2026) and the corresponding amino acid sequences were used as reference sequences (Supplementary Table S1). When multiple transcript isoforms were annotated for the same gene, only the longest transcript was retained; its corresponding protein sequence and, where required, its coding DNA sequence (CDS) was used for phylogenetic reconstruction. Homologous proteins were searched in Ensembl release 105 using BLASTP (https://blast.ncbi.nlm.nih.gov/Blast.cgi?PAGE=Proteins, accessed on 26 February 2026) [43]. All BLASTP settings were retained at the Ensembl interface defaults. Specifically, the e-value threshold was set to 1 × 10^−5^; the remaining settings were word size 3, BLOSUM62 scoring matrix, gap-opening and gap-extension penalties of 11 and 1, respectively, gapped alignment enabled, SEG low-complexity filtering enabled, composition-based score adjustment mode 2, and a maximum of 100 reported hits [43].

The human CAPN-family repertoire was used to retrieve calpains, human cathepsin genes were used to retrieve cathepsin homologs, and genes encoding proteasome-related and selected UPS components were retrieved using the same strategy. All reported hits meeting the E-value threshold were screened; no additional fixed minimum percentage-identity or query-coverage threshold was applied. Multiple transcript records from the same genomic locus were collapsed to the longest annotated transcript. Candidate loci were retained when their annotation and sequence similarity supported membership of the relevant family and were then evaluated by phylogenetic placement. Additional within-species BLASTP searches using the same settings were performed to recover paralogs potentially missed by the initial annotation.

The species included in the comparative analysis were selected to cover key vertebrate lineages and to provide context for the timing of duplication events. The taxonomic sampling followed the same comparative rationale for all three proteolytic systems and included teleost representatives to examine patterns of gene retention and diversification after the teleost-specific whole-genome duplication (Ts3R), together with two non-teleost *Actinopterygii* species that diverged before this duplication and therefore helped distinguish teleost-specific duplications from older vertebrate events. In addition, *Sarcopterygii* representatives were included to represent vertebrate lineages that experienced the two early rounds of whole-genome duplication (1R/2R), but not the teleost-specific duplication, while *Callorhinchus milii* was used as a chondrichthyan outgroup to provide broader phylogenetic context. Where reliable sequences were available, *Ciona intestinalis* was also included to provide information on the pre-vertebrate repertoire of the calpain family. The exact taxon varied among proteolytic systems and individual trees according to sequence availability, annotation quality, and reliable homolog recovery. The complete species list and genome assembly accessions are provided in Supplementary Table S2 and the species and sequence identifiers used for each individual phylogenetic reconstruction are provided in Supplementary Tables S5–S27.

#### 2.1.2. Sequence Alignment and Phylogenetic Reconstruction

The retrieved protein sequences were initially used for phylogenetic reconstruction because amino-acid data are generally less susceptible to saturation and compositional biases arising from synonymous substitutions, particularly over deeper evolutionary timescales. Complementary coding DNA sequences (CDS) based analyses were performed when protein alignments were short and failed to resolve relationships among closely related paralogs, as indicated by unresolved topology or consistently low bootstrap support at the relevant nodes.

In these cases, the corresponding CDSs were retrieved, aligned directly as nucleotide sequences in MAFFT. Nucleotide data were analyzed because they retain both synonymous and nonsynonymous variation and may therefore provide additional resolving information [44,45].

Initial multiple-sequence alignments were manually inspected and refined in AliView [46]. Pseudogenes, low-quality, incomplete, or clearly misannotated sequences were excluded before alignment. Sequences were excluded when they represented partial records that did not span the conserved homologous region observed in the corresponding orthologs or contained extended stretches of ambiguous characters that prevented reliable alignment. Multiple sequence alignments were generated using MAFFT with the default parameters (https://mafft.cbrc.jp/alignment/server/index.html; version 7 web server, accessed on 26 February 2026), and alignment quality was further evaluated with GUIDANCE2 (https://guidance.tau.ac.il, accessed on 26 February 2026), using the default GUIDANCE2 strategy (FFT-NS-1), 100 bootstrap replicates, a sequence-confidence cutoff of 0.60, and a column-confidence cutoff of 0.93. Sequences and alignment columns with confidence scores below the respective default cutoffs were excluded, while all other GUIDANCE2 parameters were maintained at their default parameters to improve confidence in the retained aligned positions [47]. Curated alignments were then imported into MEGA X (Molecular Evolutionary Genetics Analysis release 10; version 10.2.4) for downstream phylogenetic analysis [48].

For each gene family, the best-fitting substitution model was selected according to the Bayesian Information Criterion (BIC); the selected model for each reconstruction is reported with the corresponding supplementary tree. Maximum-likelihood trees were reconstructed using the selected amino acid or nucleotide model, and branch support was evaluated with 1000 bootstrap replicates. Broader family-level trees were also reconstructed from all recovered members of each proteolytic system to examine the relationships among paralogous groups. In contrast to the calpain and cathepsin family-level trees, which used broader taxonomic sampling, the UPS family-level tree used a more restricted taxon set. Specifically, five representative species were included: *Homo sapiens* as a tetrapod reference, *Sparus aurata* and *Dicentrarchus labrax* as the focal teleost species, *Oryzias latipes* as an additional well-annotated teleost representative, and *Lepisosteus oculatus* as a non-teleost actinopterygian outgroup. This reduced sampling strategy was selected because the UPS family-level tree includes functionally distinct subunit classes with substantial differences in amino acid sequence, domain composition, and evolutionary divergence. Including a very large number of species across all UPS subgroups would increase sequence heterogeneity and reduce the clarity of the family-level topology. Therefore, the aim of this tree was not to infer the detailed duplication history of each UPS gene, which was examined in the individual gene-family trees, but to visualize the broader phylogenetic separation of the main UPS subunit classes. This approach provided clearer resolution of the major UPS groups while retaining the key evolutionary comparisons needed for the present study.

Duplication timing was inferred by integrating gene-tree topology with taxonomic distribution and syntenic evidence. Topologies separating copies before the teleost/non-teleost divergence were interpreted as compatible with older vertebrate duplications, whereas duplication patterns restricted to teleost lineages were considered compatible with Ts3R. Copies restricted to particular clades or species were interpreted as compatible with lineage- or species-specific events. Duplication origins were therefore assigned based on the concordance of phylogenetic placement, taxonomic distribution, and conserved synteny.

#### 2.1.3. Synteny, Collinearity, and Duplicate-Gene Origin Classification

Phylogenetic analysis was complemented with synteny and collinearity analyses because gene trees alone cannot always distinguish among whole-genome or segmental duplication, tandem duplication, proximal duplication, dispersed duplication, and lineage-specific gene loss. BLASTP searches were performed using the command-line BLAST+ program with an E-value threshold of <1 × 10^−5^ to identify candidate paralogous regions in the genomes of gilthead seabream (GCA_900880675.1) and European seabass (GCA_905237075.1) according to Tsipourlianos et al., 2026 [49]. Ensembl annotation files for both species were parsed to generate the GFF files required by MCScanX (Multiple Collinearity Scan Toolkit, version 1.0.0) (Supplementary Table S2). Collinear blocks were identified using a MATCH_SIZE value of 2, indicating that at least two matching genes were required to define a collinear block; all other parameters were maintained at their default values. The MCScanX duplicate gene classifier was subsequently used to categorize duplicated genes according to their inferred origin. Duplicates were classified as WGD/segmental duplicates, comprising anchor genes located within collinear blocks; tandem duplicates, comprising adjacent paralogs; proximal duplicates, comprising nonadjacent paralogs located within the same genomic region; or dispersed duplicates, comprising duplicates that did not meet any of the preceding criteria. For duplicate-gene classification, “MATCH_SIZE” and “N_PROXIMAL” were set to 2 and 10, respectively, with “N_PROXIMAL” specifying the maximum number of intervening genes permitted between two proximal duplicates. These algorithmic classifications were interpreted alongside phylogenetic topology as supporting, not definitive, evidence of historical duplication origin [50]. Because detectable collinearity depends on genome assembly and annotation, cross-species discrepancies were interpreted cautiously.

### 2.2. Gene Expression Profiling

#### 2.2.1. Larval Rearing and Developmental Sampling

Egg hatching, larval rearing, and developmental sampling were performed at the Hellenic Centre for Marine Research (HCMR) as previously described [51,52]. European seabass and gilthead seabream larvae were reared at 17 and 20 °C, respectively, and sampled at first feeding (FF), notochord flexion (FL), and mid-metamorphosis (MM) [53]. Samples were preserved in RNAlater and stored at −20 °C until RNA extraction.

#### 2.2.2. RNA Extraction, Sequencing, and Transcriptome Data Processing

For the laboratory-generated transcriptomic data, larvae were homogenized using glass-bead beating using a Precellys tissue homogenizer (Bertin Technologies, Montigny-le-Bretonneux, France). Total RNA was extracted using the E.Z.N.A.^®^ Total RNA extraction kit (Omega Bio-tek, Inc., Atlanta, GA, USA), followed by DNase treatment using the DNA-free™ DNA Removal Kit (Invitrogen, Waltham, MA, USA) to remove genomic DNA contamination. RNA quality was evaluated by gel electrophoresis, and RNA concentration was measured with the Qubit™ RNA BR Assay Kit (Invitrogen, Waltham, MA, USA). Before library preparation, RNA integrity was further assessed by Novogene UK using an Agilent Bioanalyzer (Agilent Technologies, Santa Clara, CA, USA). For each species-stage combination, 12 biological samples were initially collected. At first feeding, each biological sample comprised pooled individuals to obtain sufficient RNA, whereas samples from later stages were extracted from individual larvae [40]. Equal amounts of RNA from the 12 samples within each species-stage combination were then pooled after extraction to generate one sequencing library per stage (*n* = 1 library per stage).

Libraries were sequenced on an Illumina HiSeq 2000 platform (Illumina Inc., San Diego, CA, USA) using 150-bp paired-end reads, generating approximately 30–44 million clean read pairs per sample, with Q20 values exceeding 97%. Sequencing was performed from Novogene, Cambridge, UK and the transcriptome data were deposited in the SRA (European seabass (*D. labrax*): PRJNA1050410 and gilthead seabream (*S. aurata*): PRJNA1050571). Detailed sequencing quality-control and mapping statistics are provided in Supplementary Table S28. Because the RNA-seq datasets consisted of one pooled library per developmental stage, these expression patterns should be interpreted cautiously as descriptive rather than as statistically tested stage-specific differences. Nevertheless, they provide a useful exploratory framework for comparing the relative expression of retained proteolytic paralogs across ontogenesis.

Raw sequencing reads were processed according to the pipeline described by Tsipourlianos et al. (2026) [54]. Briefly, read quality was assessed using FastQC v0.12.1 [55], and quality-control reports were summarized with MultiQC v1.14. Transcript abundance was quantified with Salmon v1.10.1 in quasi-mapping mode against the corresponding reference transcriptome (*Dicentrarchus*_*labrax*.seabass_V1.0.105 and *Sparus_aurata*.fSpaAur1.1.116). Salmon mapping statistics were inspected together with the quality-control reports to evaluate overall dataset quality [56]. Transcript-level abundance estimates were subsequently imported into R using tximport v1.28.0 [57] and summarized at the gene level for downstream normalization and targeted gene-expression interpretation. Transcript identifiers and gene annotations were obtained from the corresponding Ensembl GTF files, which were also used to generate transcript-to-gene mapping tables for gene-level summarization. When datasets from different sources were combined, potential batch effects were corrected using the ComBat-Seq model from the sva R package [58,59]. ComBat-Seq was applied to untransformed gene-level count estimates before the calculation or log transformation of expression values. BioProject or sequencing study was specified as the batch variable, whereas tissue or developmental stage was included as the biological grouping variable to be preserved. This correction was performed solely to reduce technical variation among sequencing sources. However, because the datasets differed in sampling design and source, residual study-associated effects cannot be excluded. Expression values were then extracted for the standardized UPS, calpain, and cathepsin gene sets. Scripts used are deposited on GitHub platform (https://github.com/RafaelAngelakopoulos/Bioz_lab/tree/main/RNAseq, accessed on 9 September 2026).

#### 2.2.3. Developmental and Tissue-Specific Expression Analysis

Expression profiling was performed using three transcriptomic data sources. First, RNA-seq data generated as described above, were used for post-hatching developmental stages of both European seabass and gilthead seabream, corresponding to first feeding (FF), notochord flexion (FL), and mid-metamorphosis (MM). Second, publicly available embryonic RNA-seq data for European seabass (*D. labrax*) from the AQUAFAANG project were included (BioProject ID: PRJEB52160), allowing expression patterns to be examined from early embryogenesis to hatching (*n* = 3 per stage). Comparable embryonic transcriptomic data were not available for gilthead seabream. Nevertheless, we retained the European seabass embryonic dataset because it provides additional biologically relevant information on proteolytic-gene expression during earlier development. Third, publicly available transcriptomic datasets from adult tissues of both species were used to examine tissue-specific expression profiles (Supplementary Table S3).

For developmental expression analysis, embryonic stages were evaluated only in European seabass, whereas post-hatching stages were compared in both European seabass and gilthead seabream. For tissue-specific expression analysis, adult tissue transcriptomes were analyzed in both species to determine whether duplicated paralogs showed broad expression, tissue-restricted expression, or absence from specific tissues.

For each proteolytic system, expression patterns of paralogous genes were compared within each species and, where common tissues were available, between gilthead seabream and European seabass. Heatmaps were generated using pheatmap to visualize expression differences among paralogs across developmental stages and adult tissues using log_2_(TPM + 0.25) values. The log_2_ transformation was applied to compress the wide dynamic range of expression values and reduce the visual dominance of highly expressed genes, while 0.25 was added as a pseudocount to permit the transformation of zero TPM values [60,61,62]. Paralog expression profiles were qualitatively examined to identify broad similarities, predominance of one copy, and stage- or tissue-associated variation. The analysis was designed to provide a descriptive comparison of expression patterns across developmental stages and tissues and not as differential expression analysis.

To examine coordinated expression among UPS components, pairwise Spearman correlation coefficients were calculated between genes across the profiles within each dataset; developmental and tissue datasets and the two species were analyzed separately. The European seabass developmental matrix comprised 18 stages and 40 aggregated UPS components, the gilthead seabream developmental matrix comprised three pooled stage-level profiles and 51 components, and the European seabass and gilthead seabream tissue matrices each comprised four adult tissues and 39 and 50 components, respectively. For subunits represented by multiple numbered paralogs, TPM values were summed before applying the transformation log_2_(TPM + 0.25), thereby estimating their combined subunit-level expression. Zero values were retained through the pseudocount, no missing values were present in the analyzed matrices, and invariant profiles were excluded because their correlations are undefined. Correlations were descriptive: *p*-values were not interpreted, no multiple-testing correction or significance threshold was applied, and qualitative labels such as strong or weak were not used for inference. Matrices were visualized as heatmaps with genes grouped as 20S core, 19S regulator, proteasome activator, specialized core, or assembly/other regulator components.

## 3. Results

### 3.1. Phylogenetic Analysis and Gene Repertoire Identification

Using the HGNC database as a reference, we first identified genes encoding components of three intracellular proteolytic systems in humans, namely the ubiquitin–proteasome system (UPS), calpains and cathepsins, and subsequently searched for homologous and paralogous sequences in the teleost genomes under study. The names assigned to fish paralogs and their human orthologs were frequently inconsistent in the Ensembl annotations, and several sequences were annotated as novel genes. Therefore, to improve clarity throughout the manuscript, a standardized nomenclature was adopted. Each teleost gene was assigned a name from the combined evidence of sequence similarity and phylogenetic placement, using the closest supported human homolog as the reference; ascending numbers were appended to distinguish paralogs.

For the UPS, 51 human genes encoding UPS subunits were identified and used as reference sequences. A larger repertoire was recovered in both focal teleost species, with 61 UPS-related genes identified in gilthead seabream (*Sparus aurata*) and 53 in European seabass (*Dicentrarchus labrax*) (Table 1).

Phylogenetic reconstruction indicated that the retained paralogs of these UPS gene families were mostly compatible with an origin in the teleost-specific whole-genome duplication (Ts3R). For the calpain family, 16 CAPN-family genes were identified in humans, 15 of which had orthologs in teleosts. A larger repertoire was recovered in the two focal species, with 29 calpain genes identified in gilthead seabream and 22 in European seabass. For the cathepsin family, 15 human cathepsin genes were used as starting queries. Orthologs were recovered in teleosts for 11 genes, namely *ctsa*, *ctsb*, *ctsc*, *ctsd*, *ctsf*, *ctsh*, *ctsk*, *ctsl*, *ctso*, *ctss* and *ctsz*. In total, 23 cathepsin genes were identified in gilthead seabream and 17 in European seabass. The correspondence between standardized gene names and Ensembl gene IDs is provided in Supplementary Table S4, while complete species-level correspondences are provided in Supplementary Tables S5–S27.

Maximum-likelihood phylogenetic trees were reconstructed for each gene family separately, and family-level trees were also produced for all recovered UPS, calpain, and cathepsin sequences. Across the three proteolytic systems, several genes showed a consistent single-copy pattern across the species examined. This single-copy pattern predominated in the UPS. In calpains, the phylogenetic analysis of *capn1*, *capn10*, *capn12*, *capn7*, and *capn9* revealed the presence of a single copy in all species studied. In cathepsins, *ctsa*, *ctso*, *ctsk*, *ctsf*, and *ctsc* were also recovered as single-copy genes across the species studied.

**Figure 1:**
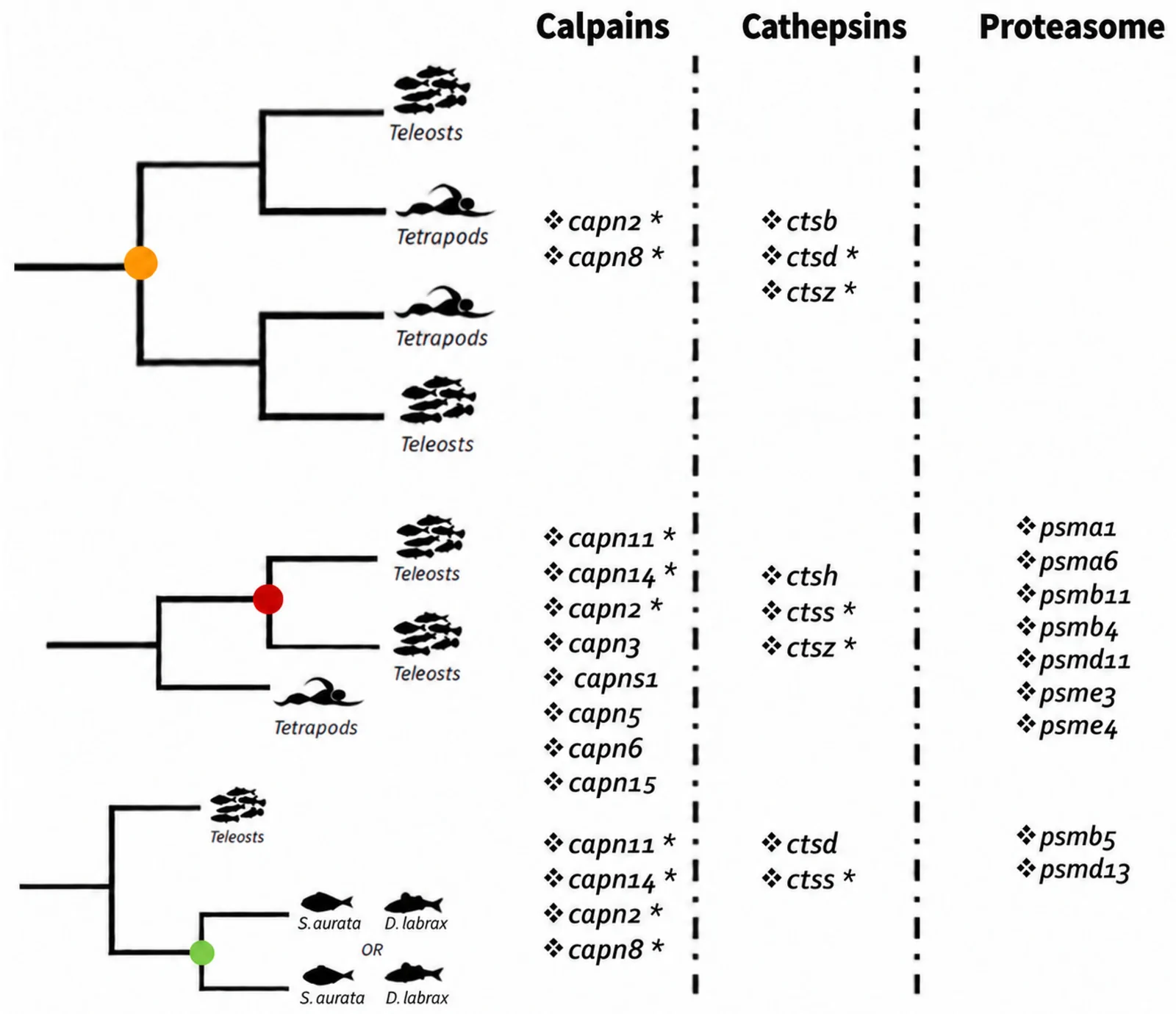
Correspondence between gene families and phylogenetic topologies that may explain the origin of the gene families. Orange denotes patterns compatible with an older vertebrate duplication (1R/2R), red denotes patterns compatible with duplication near the base of teleosts (Ts3R) and lineage- or species-specific duplications are indicated in green. Asterisks identify families containing copies associated with more than one inferred history.

Three recurring phylogenetic topologies were considered to infer the likely timing of duplication events. The first topology corresponded to duplication events inferred before the teleost/non-teleost divergence and was therefore compatible with the first or second round of vertebrate genome duplication (1R/2R). The second topology placed a duplication near the base of teleosts, compatible with the Ts3R. The third topology indicated lineage- or species-specific duplication events (Figure 1). These topology-based assignments were not treated as proof of a specific duplication mechanism and were evaluated with taxonomic distribution, branch support, and, where available, syntenic evidence.

**Figure 2:**
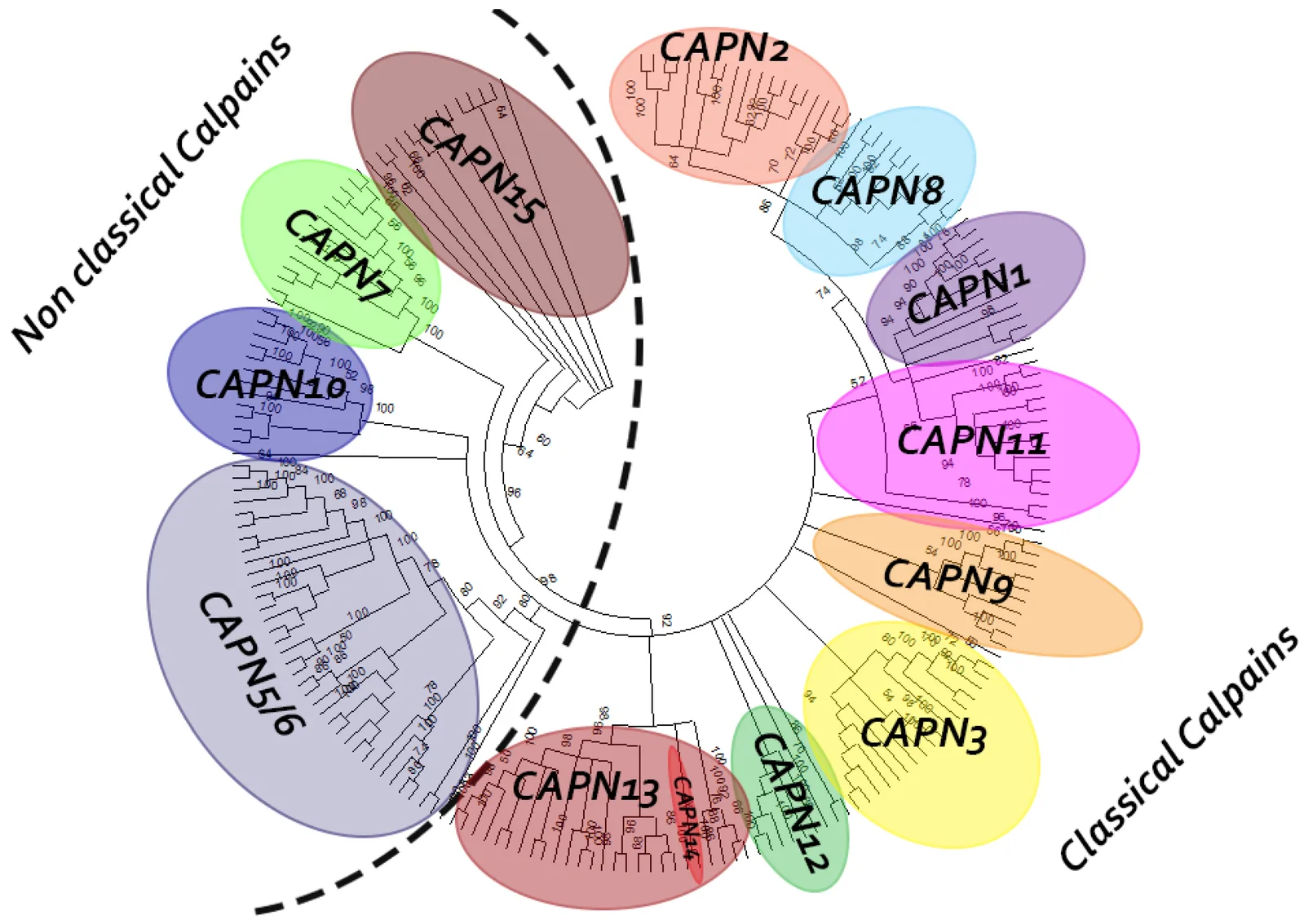
Unrooted maximum-likelihood family-level phylogeny of vertebrate calpains. The tree separates classical and non-classical calpains. Each gene family is color coded for illustration purposes.

In calpains, duplicated gene families followed all three topological patterns, indicating that the present-day calpain repertoire was shaped by multiple evolutionary events rather than by a single duplication episode. Some duplicated families, including *capn2* and *capn8*, showed a topology compatible with an older duplication event predating the divergence of teleost and non-teleost vertebrates. Several other calpain families, including *capn11*, *capn14*, *capn2*, *capn3*, *capns1*, *capn5*, and *capn6*, showed topologies compatible with duplication near the base of teleosts, suggesting retention after the teleost-specific genome duplication. In addition, lineage- or species-specific patterns were observed for *capn11*, *capn14*, *capn2*, and *capn8*, indicating that additional duplication or retention/loss events occurred after the diversification of teleost lineages (Supplementary Figures S1–S9). The individual supplementary trees display bootstrap support at the relevant nodes; assignments were not made when the focal relationships lacked sufficient support. The family-level calpain phylogeny further supported the subdivision of the repertoire into classical and non-classical calpains. Non-classical calpains formed distinct clades including *capn5*, *capn6*, *capn7*, *capn10*, *capn13* and *capn15*, whereas classical calpains included *capn1*, *capn2*, *capn3*, *capn8*, *capn9*, *capn11*, and *capn12*. Within the classical calpain group, *capn11*, *capn1*, *capn8*, and *capn2* were placed in closely related branches (bootstrap value = 74), supporting a close evolutionary relationship among these lineages (Figure 2).

**Figure 3:**
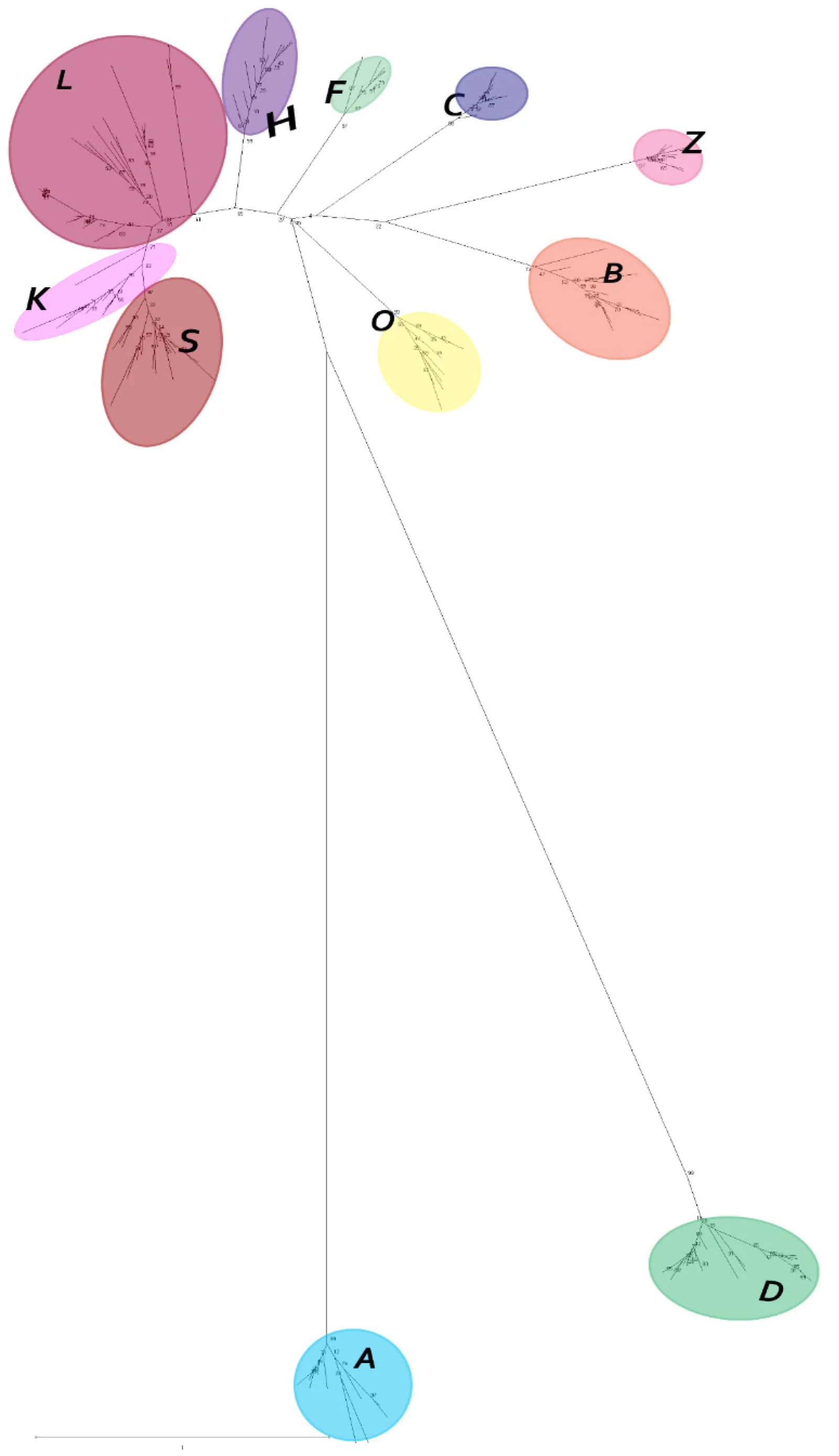
Unrooted family-level phylogenetic tree of cathepsins in vertebrates. The cathepsin tree separates *ctsa* and *ctsd* from cysteine cathepsins and resolves two broad cysteine cathepsin clades. Each gene family is color coded for illustration purposes.

The phylogenetic analysis of cathepsins revealed a different but similarly complex pattern. A family-level tree separated the cysteine proteases from the serine cathepsin *ctsa* and the aspartic cathepsin *ctsd*, which were more distantly related. Within the cysteine cathepsins, two broad clades were recovered: one containing *ctss*, *ctsk*, *ctsl*, *ctsh*, and *ctsf*, and another containing *ctsc*, *ctsz*, *ctsb*, and *ctso* (Figure 3). Among duplicated cathepsins, the *ctsb* tree suggested a duplication event in the common ancestor of teleost and non-teleost vertebrates, most likely associated with the second round of vertebrate genome duplication. A similar topology was observed for *ctsd*, which was compatible with an older duplication event before the divergence of ray-finned and lobe-finned fishes. The *ctsh* tree revealed a duplication node consistent with the TS3R, while *ctss* also showed multiple copies within teleost species and a topology compatible with duplication at the base of teleosts. The *ctsz* topology suggested two duplication nodes, one compatible with an older vertebrate duplication and another within the teleost lineage (Supplementary Figures S10–S14). Bootstrap values are displayed on each supplementary tree. The *ctsl* branches were insufficiently supported, and no specific duplication history was assigned. The *ctsl* tree contained several duplication signals, but the branches were not sufficiently supported by high bootstrap values; therefore, its evolutionary interpretation remained uncertain.

**Figure 4:**
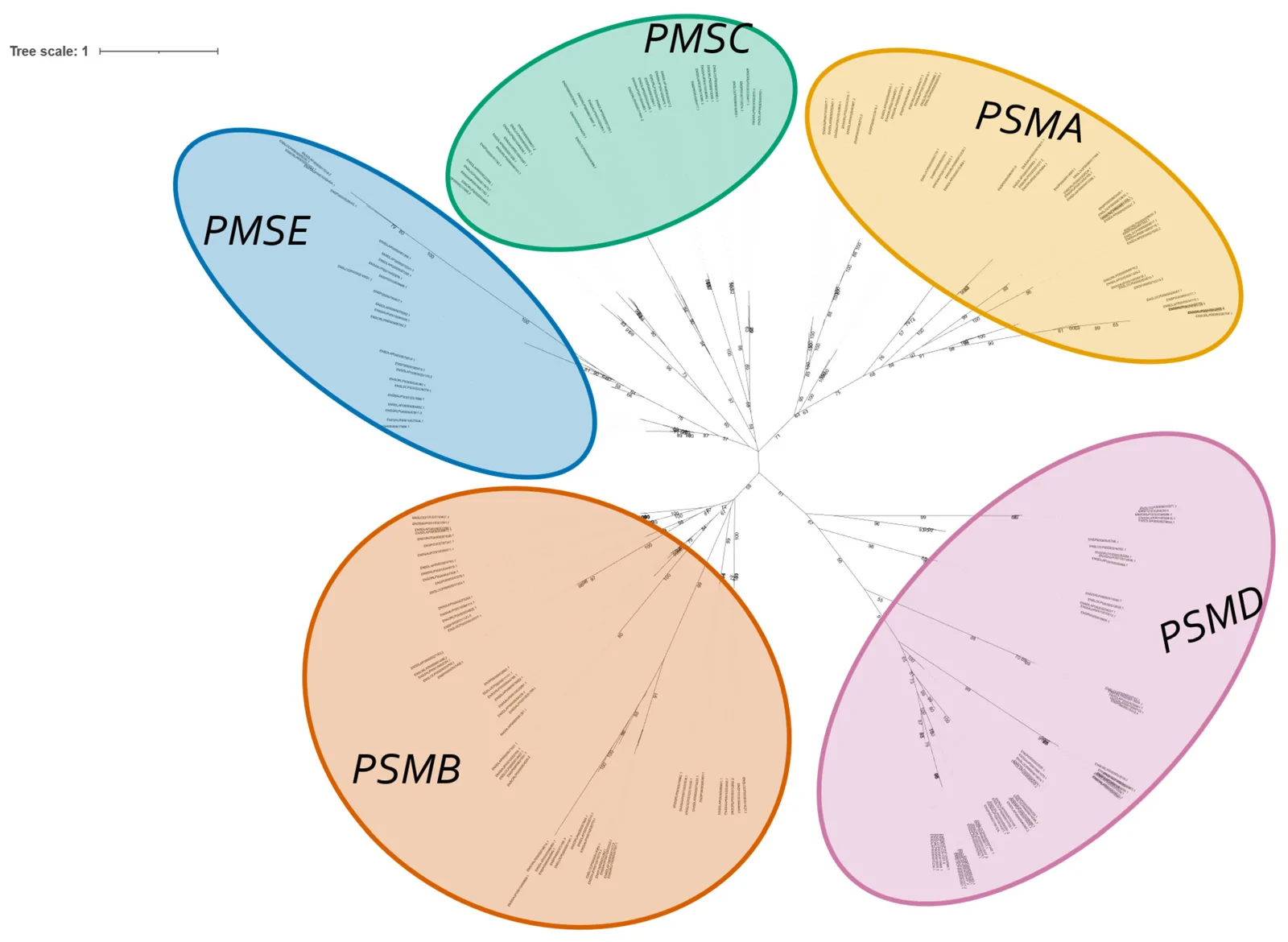
Unrooted family-level phylogenetic tree of the UPS in selected representatives of tetrapods (*H. sapiens*) and *Actinopterygii* (*Holostei* (*L. oculatus*); *Teleostei* (*S. aurata*, *D. labrax*, *O. latipes*)). The UPS tree resolves five distinct clades corresponding to the major UPS subunit classes. Each gene family is color coded for illustration purposes.

The family-level phylogenetic reconstruction of UPS genes separated as expected the repertoire into distinct clades corresponding to the major proteasome-related subgroups, namely *PSMA*, *PSMB*, *PSMC*, *PSMD*, and *PSME*. The recovered topology was consistent with the organization of the different UPS components, with members of each functional subunit class clustering into separate branches (Figure 4). Duplication histories were therefore evaluated primarily from the individual gene-family trees.

Duplicated UPS gene families in gilthead seabream included *psma4*, *psma6*, *psmb4*, *psmb11*, *psmd4*, *psmd11*, *psmd13*, *psme3* and *psme4*, whereas duplicated UPS gene families in European seabass included *psma4*, *psma6*, *psmb11*, *psmd4*, *psmd11* and *psme4*. These duplicated gene families were generally recovered as retained paralogs following a topology compatible with the TS3R. The genes *psmb4* and *psmd13* were duplicated only in gilthead seabream and may have arisen through lineage-specific duplication. The *psma6* tree contained several duplication signals, but the branches were not sufficiently supported by high bootstrap values; therefore, its evolutionary interpretation remained uncertain (Supplementary Figures S15–S22).

### 3.2. Synteny Analysis and Duplicate-Gene Origin Classification

Synteny analysis was used to provide genomic context for duplicated proteolytic genes in gilthead seabream and European seabass. MCScanX identified collinear blocks, and its duplicate-gene classifier assigned each copy to a duplication category. These assignments were interpreted as supporting evidence complementary to the phylogenetic results.

Most duplicated UPS genes were classified as WGD/segmental or dispersed in at least one species, which along with the phylogenetic analysis is consistent with paralogs retention following the Ts3R. However, *psmb4* and *psmd13* were detected as duplicated only in gilthead seabream and were classified as tandem and proximal duplicates, respectively. For calpains, duplicated genomic regions were also identified in both species, but duplicate-gene classification indicated mixed origins. The *capn2* family showed WGD/segmental signal, being classified as WGD/segmental in both species and additionally as proximal in gilthead seabream in agreement with the phylogenetic topology that showed a duplication compatible with WGD events. In gilthead seabream, *capn5*, *capn8*, and *capn11* also showed WGD/segmental or mixed WGD-related classifications, whereas *capn14* included a tandem component. In European seabass, *capn3* was classified as WGD/segmental, while several other duplicated calpain families were classified as dispersed. For cathepsins, duplicated genes were mostly classified as dispersed, although WGD/segmental signals were detected for specific families. In gilthead seabream, *ctsd* and *ctss* were classified as WGD/segmental and tandem, whereas their European seabass counterparts were classified as dispersed. The *ctsb*, *ctsl*, and *ctsz* families were classified as dispersed in both species, while *ctsh* was classified as WGD/segmental in European seabass (Table 2). Because MCScanX classifications reflect present-day genomic organization, classification as dispersed does not necessarily exclude retention following the Ts3R. Accordingly, dispersed paralogs whose phylogenetic topologies were consistent with duplication at the Ts3R node were interpreted as putative WGD-derived paralogs. Subsequent chromosomal rearrangements, translocations, gene loss, genome fractionation, or erosion of detectable collinearity may obscure the ancestral duplicated regions and cause WGD-derived paralogs to be classified as dispersed. Therefore, discordance between the two species may reflect genuine lineage-specific differences or post-WGD genome restructuring, although differences in genome assembly, annotation quality, and detectable collinearity may also contribute.

### 3.3. Gene Expression Profiling During Development and in Adult Tissues

Whole-transcriptome sequencing data from public repositories and from analyses performed by the Laboratory of Genetics, Comparative and Evolutionary Biology were used to examine developmental expression patterns of calpain, cathepsin and UPS genes. The datasets included post-hatching stages in gilthead seabream and European seabass, namely onset of external feeding (FF), notochord flexion (FL) and mid-metamorphosis (MM). For European seabass, embryonic-stage data were also included, allowing expression profiles to be followed from embryogenesis to post-hatching development.

#### 3.3.1. Ontogenetic Expression Patterns

Developmental expression profiles differed among proteolytic systems, species and paralogs. In European seabass, expression was examined across embryonic and post-hatching developmental windows, whereas in gilthead seabream expression was examined at FF, FL and MM. The heatmaps showed broadly comparable abundance between some copies, predominance of one copy in other pairs, and low or stage-associated abundance of individual paralogs. Because the post-hatching series lacked biological replication, these observations are descriptive rather than statistically tested stage-specific differences.

**Figure 5:**
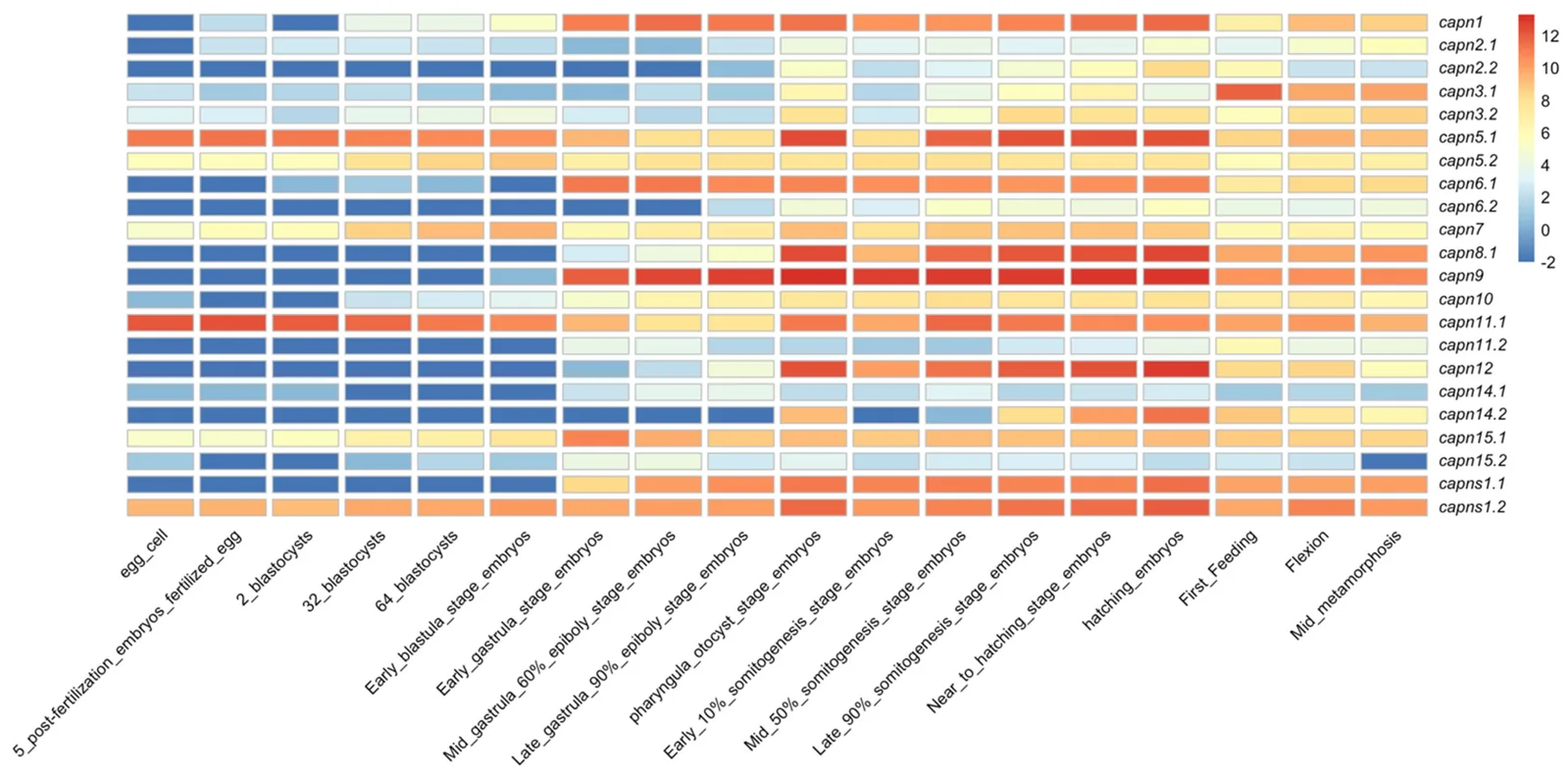
Heatmaps of calpain gene expression during development in European seabass across embryonic and post-hatching developmental windows. Rows are genes and columns are the chronologically ordered stages labeled on the *x*-axis. Colors represent log_2_(TPM + 0.25) according to the displayed scale.

**Figure 6:**
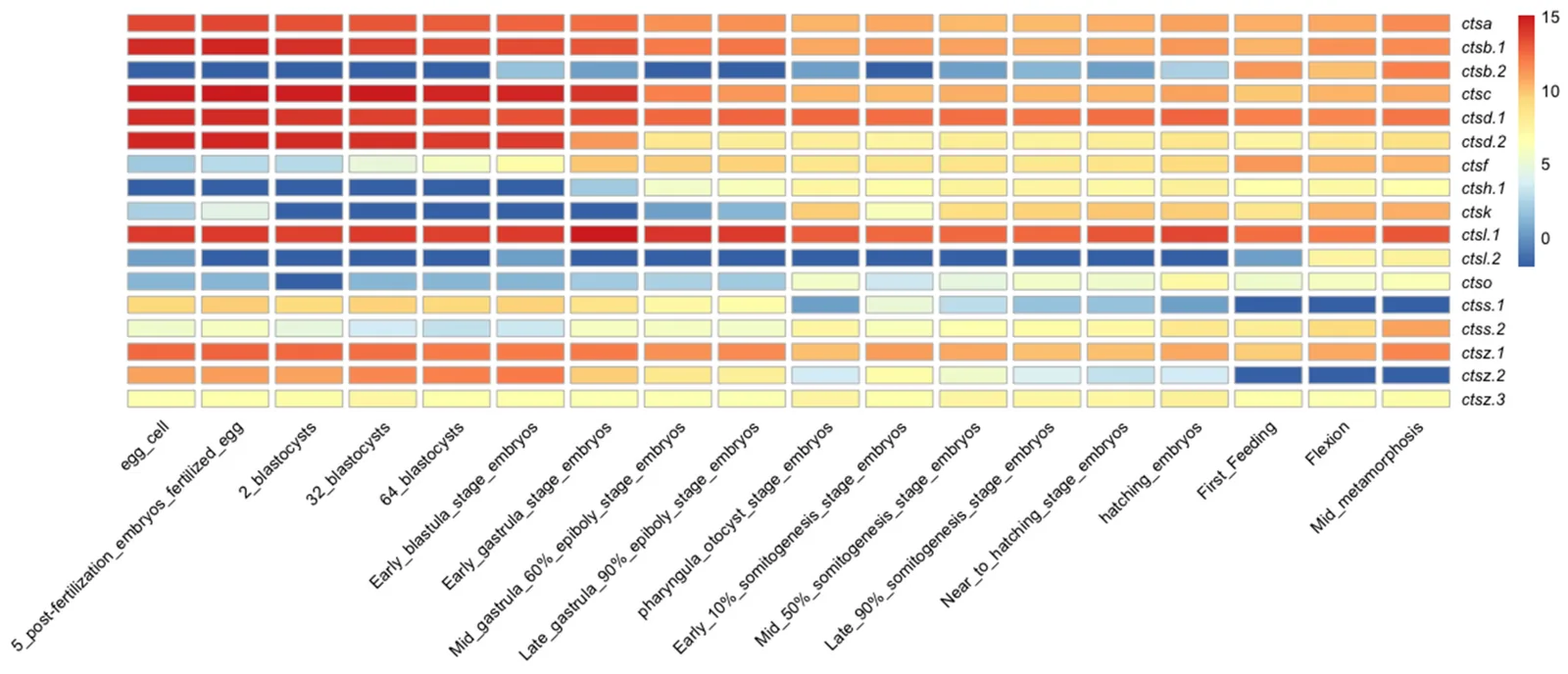
Heatmaps of cathepsin gene expression during development in European seabass across embryonic and post-hatching developmental windows. Rows are genes and columns are the chronologically ordered stages labeled on the *x*-axis. Colors represent log_2_(TPM + 0.25) according to the displayed scale.

**Figure 7:**
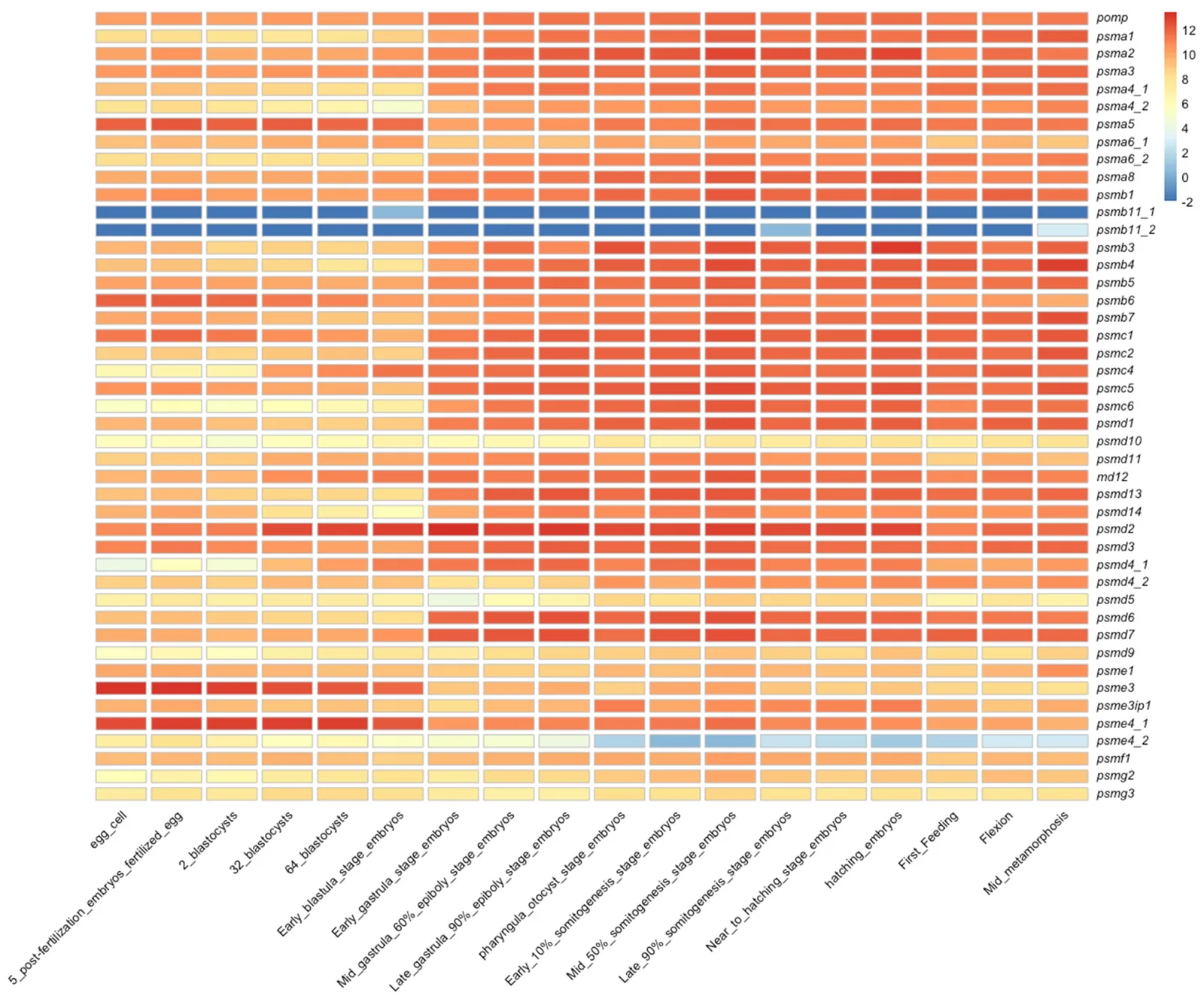
Heatmaps of UPS gene expression during development in European seabass across embryonic and post-hatching developmental windows. Rows are genes and columns are the chronologically ordered stages labeled on the *x*-axis. Colors represent log_2_(TPM + 0.25) according to the displayed scale.

**Figure 8:**
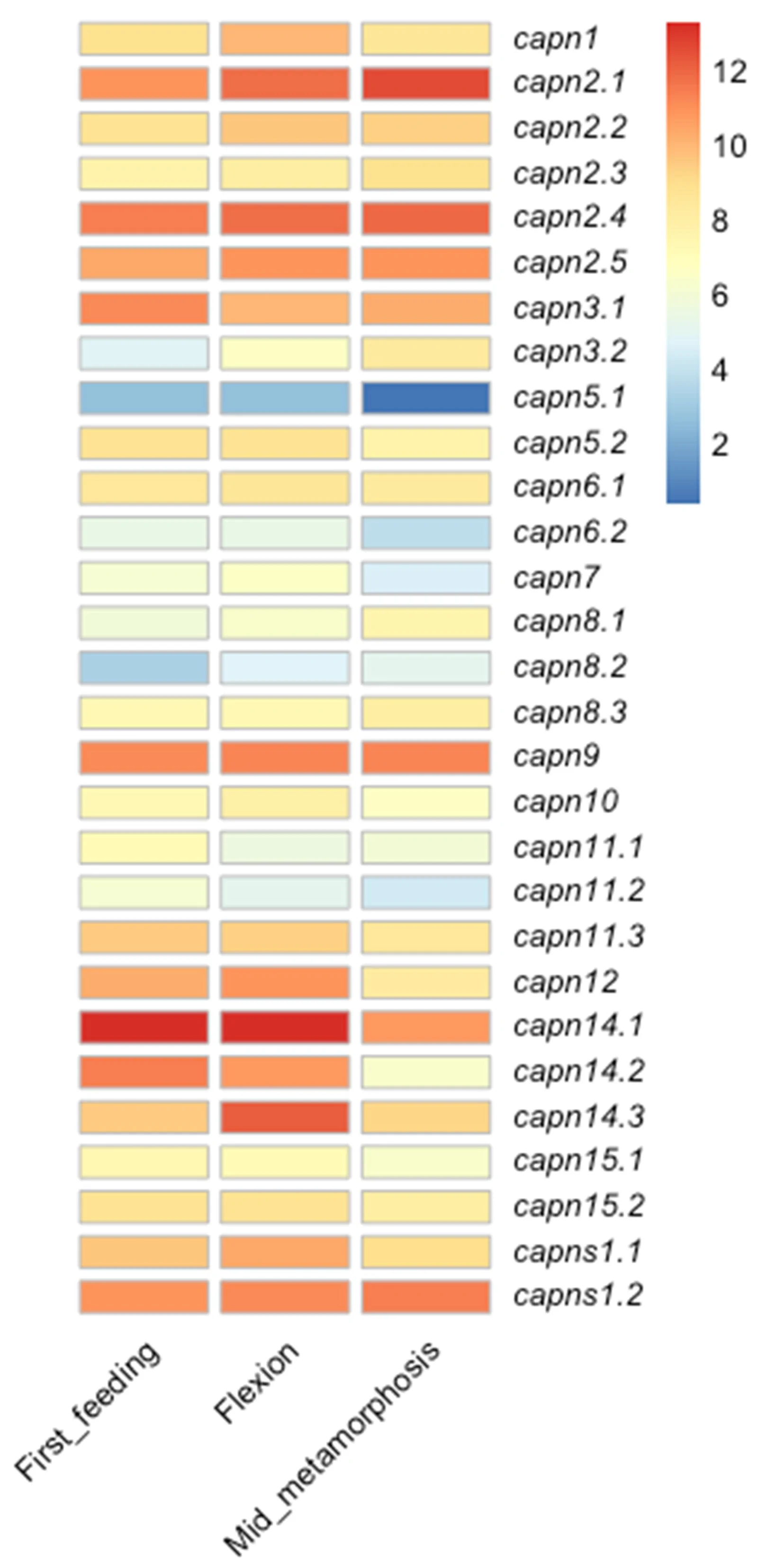
Heatmaps of calpain gene expression during development in gilthead seabream at first feeding, flexion, and mid-metamorphosis. Rows are genes and columns are the chronologically ordered stages labeled on the *x*-axis. Colors represent log_2_(TPM + 0.25) according to the displayed scale.

**Figure 9:**
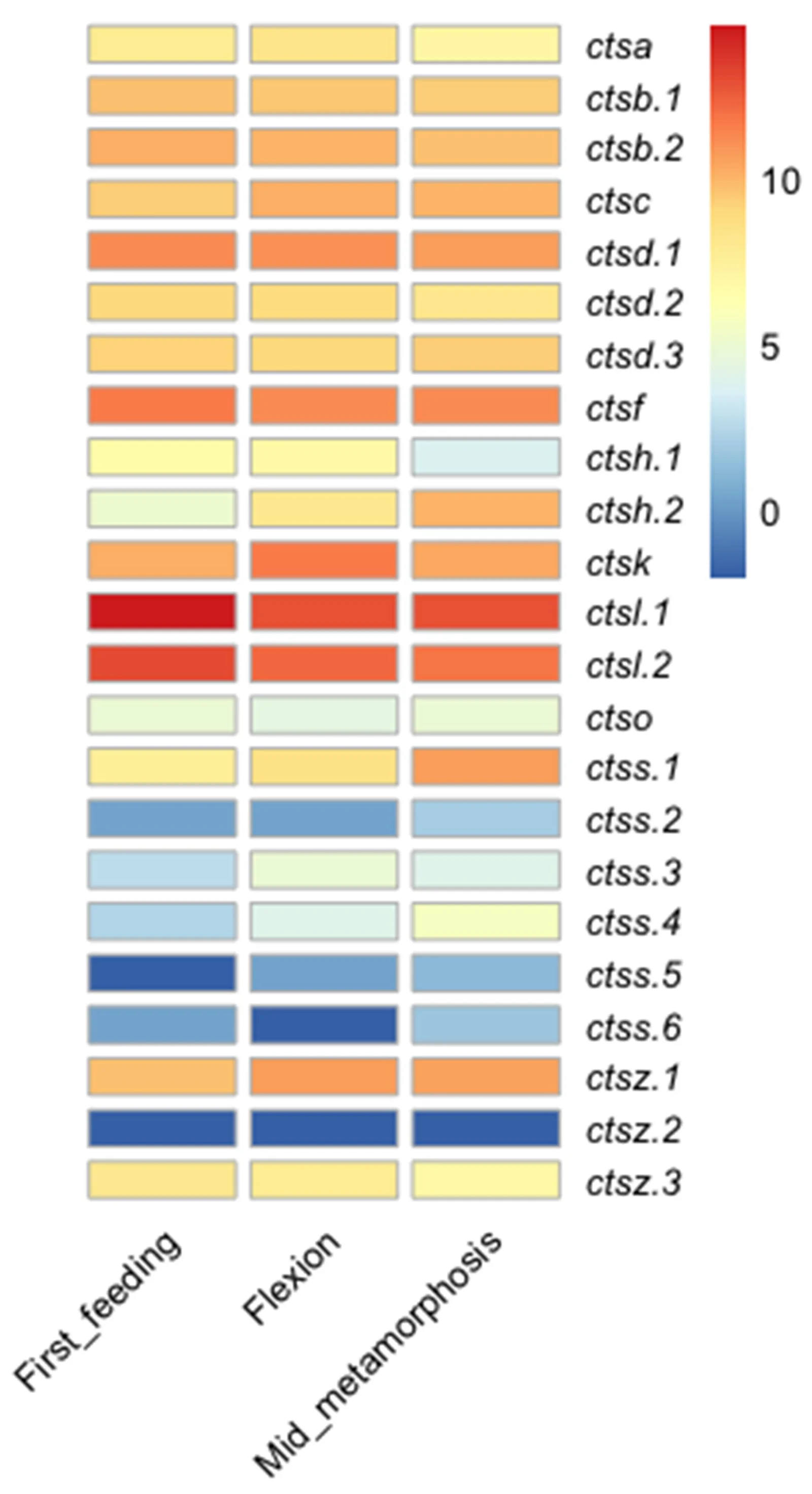
Heatmaps of cathepsin gene expression during development in gilthead seabream at first feeding, flexion, and mid-metamorphosis. Rows are genes and columns are the chronologically ordered stages labeled on the *x*-axis. Colors represent log_2_(TPM + 0.25) according to the displayed scale.

**Figure 10:**
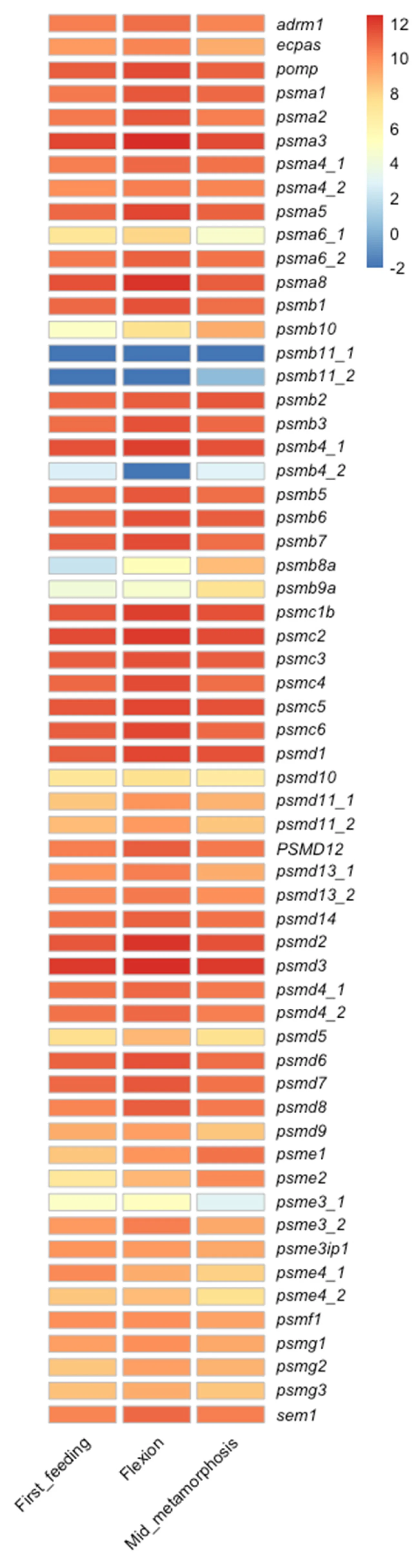
Heatmaps of UPS gene expression during development in gilthead seabream at first feeding, flexion, and mid-metamorphosis. Rows are genes and columns are the chronologically ordered stages labeled on the *x*-axis. Colors represent log_2_(TPM + 0.25) according to the displayed scale.

Calpain transcript abundance varied among paralogs. In gilthead seabream, *capn14.1* was among the most abundant calpain transcripts at FF and FL, while *capn9* and *capn14.1* showed higher TPM values at MM. *capn2* copies were detected at all three stages, *capn3* displayed an asymmetric abundance pattern between copies, and several *capn8* and *capn11* copies showed lower transcript abundance. In European seabass, most calpain transcripts showed low transcript abundance or were not detected during early cleavage, except *capn11.1*, *capn5.1*, and *capns1.2*; the number of calpain genes with detectable transcript abundance increased from gastrulation to hatching (Figure 5, Figure 6, Figure 7 and Figure 8). Cathepsin profiles also varied. In gilthead seabream, all three *ctsd* copies were detected at FF, FL, and MM; *ctsl* was among the more abundant cysteine-cathepsin transcripts; and most *ctss* copies showed low TPM values or were not detected except *ctss.1*, *ctss.3*, and *ctss.4*. In European seabass, *ctsd* and *ctsa* showed high transcript abundance early in development, *ctsl.1* remained detectable after gastrulation, and *ctsb.2* and *ctsl.2* showed higher abundance after hatching, whereas *ctss.1* and embryonic *ctsz.2* were not detected post-hatching (Figure 6, Figure 7, Figure 8 and Figure 9). Most UPS transcripts were detected throughout development in both species, with lower abundance for selected duplicates, including *psmb11* copies (Figure 7, Figure 8, Figure 9 and Figure 10).

Overall, UPS genes were broadly detected across the sampled developmental stages, whereas calpain and cathepsin paralogs showed more varied stage-associated abundance.

#### 3.3.2. Tissue-Specific Expression Patterns

Adult-tissue profiles were examined in gilthead seabream and European seabass to describe broad detection, tissue-associated differences, and non-detection of duplicated UPS, calpain, and cathepsin genes in the analyzed datasets.

**Figure 11:**
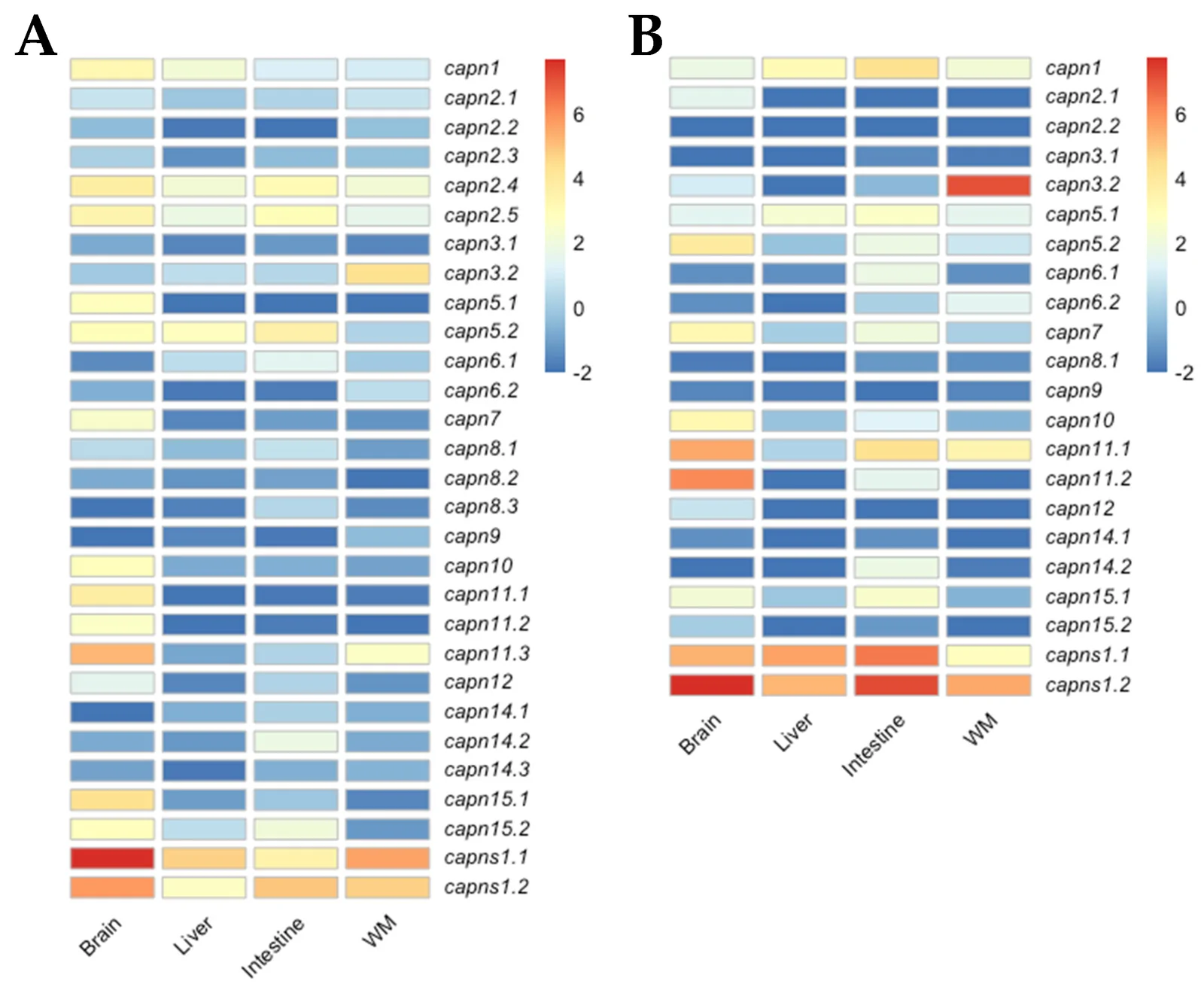
Heatmaps of tissue-specific expression of calpain paralogs in (**A**) adult gilthead seabream tissues and (**B**) adult European seabass tissues. Rows represent genes and columns represent the adult tissues analyzed. Colors represent log_2_(TPM + 0.25) according to the displayed scale.

In gilthead seabream, calpain paralogs displayed tissue-associated differences. The *capn11* paralogs had the highest combined abundance in the brain, whereas the *capn14* copies were more abundant in the intestine and lower in the brain. Several *capn8*, *capn5*, and *capn6* copies were not detected in selected tissues; *capns1* and *capn15* were broadly detected but differed in the predominant copy (Figure 11A). In European seabass, calpain paralogs also showed tissue-dependent expression patterns. *Capn11.2* was non detected in liver and white muscle, while *capn11.1* showed higher abundance in brain and intestine. *Capn14* and *capn2* paralogs were non detected in several tissues, and *capn3*, *capn5*, *capn6*, *capn15* and *capns1* also differed in abundance level or presence. *Capn11* and *capns1* showed similar patterns between species, whereas *capn2* showed the strongest interspecific differences in both copy number and abundance (Figure 11B). Non-detection does not imply absence of biological function.

**Figure 12:**
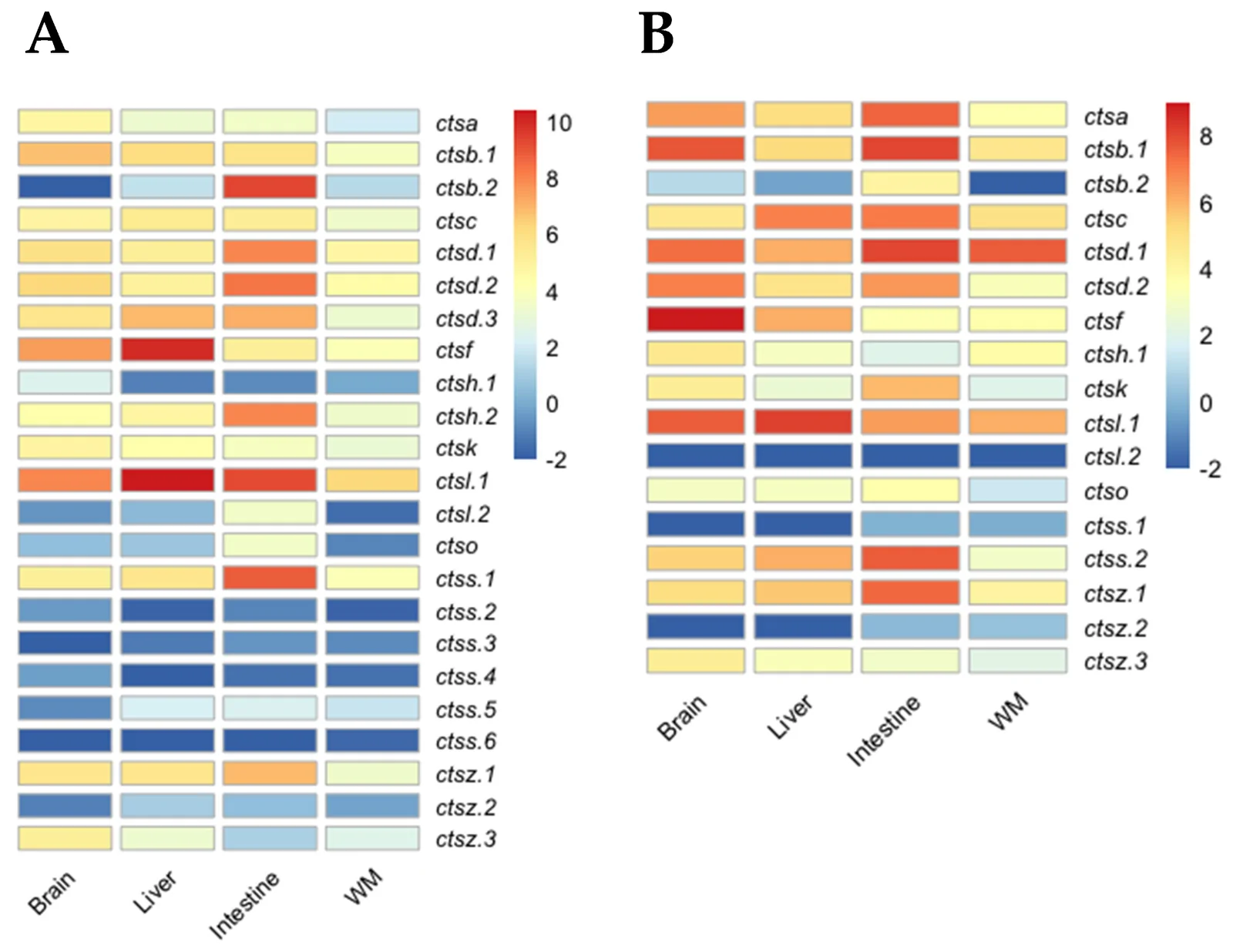
Heatmaps of tissue-specific expression of cathepsin paralogs in (**A**) adult gilthead seabream tissues and (**B**) adult European seabass tissues. Rows represent genes and columns represent the adult tissues analyzed. Colors represent log_2_(TPM + 0.25) according to the displayed scale.

Cathepsin paralogs also showed tissue-associated expression patterns. In gilthead seabream, all three *ctsd* copies were detected in all tissues, although the predominant copy differed. *ctsb.2* was not detected in brain, and at least two *ctss* copies were detected in most tissues (Figure 12A). In European seabass, *ctsd* copies had comparable abundance in brain, while *ctsd.1* was more abundant in intestine, liver, and white muscle. *ctsb.2* was not detected in white muscle; *ctsl.2* was not detected in brain, intestine, liver, or white muscle; and *ctsz.2* and *ctss.1* were not detected in brain or liver. *ctsd* and *ctss* were broadly similar between species, whereas *ctsz* and particularly *ctsl* differed in tissue detection and copy-specific abundance (Figure 12B).

**Figure 13:**
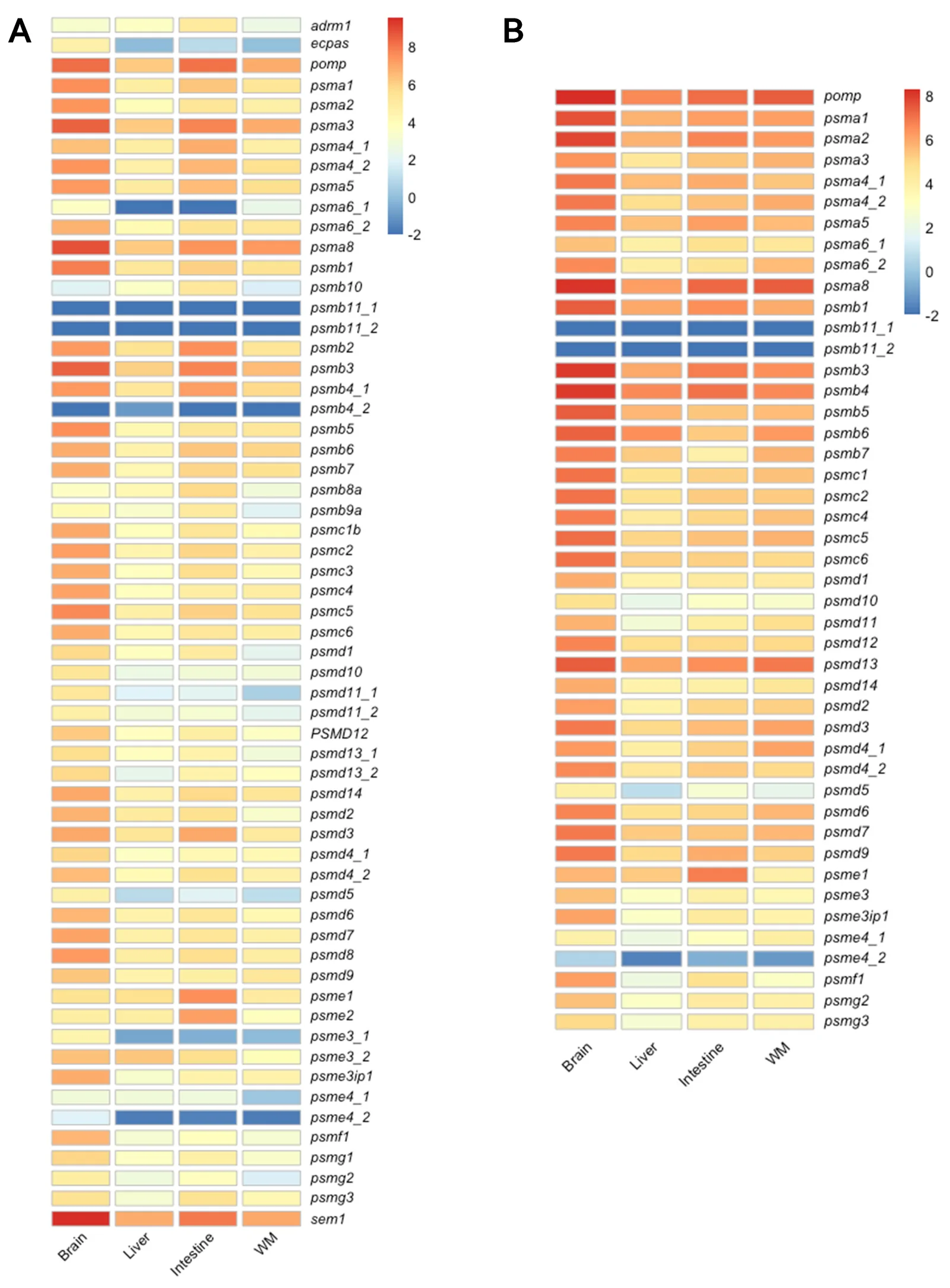
Heatmaps of tissue-specific expression of UPS paralogs in (**A**) adult gilthead seabream tissues and (**B**) adult European seabass tissues. Rows are genes and columns are the chronologically ordered stages labeled on the *x*-axis. Colors represent log_2_(TPM + 0.25) according to the displayed scale.

UPS genes showed broad tissue expression patterns in both species, with most subunits detected in all adult tissues examined. In gilthead seabream, several UPS genes were more abundant in brain, while duplicated genes such as *psma4*, *psma6*, *psmb11*, *psmd4* and *psme4* displayed different abundance patterns between copies. The *psmb11* paralogs and *psme4.2* showed lower abundance than most UPS genes across tissues (Figure 13A). In European seabass, duplicated UPS genes also showed paralog-specific differences, including *psma4*, *psma6*, *psmb11*, *psmb4*, *psmd11*, *psmd13*, *psmd4*, and *psme4*. Several of these pairs showed asymmetric abundance patterns between copies, with *psmb11*, *psmb4.2* and *psme4.2* being among the less abundant transcripts (Figure 13B).

#### 3.3.3. Coexpression Patterns of UPS Components

Positive correlations predominated among constitutive 20S and 19S proteasome subunits in all four matrices (Supplementary Figures S24–S27). Among the constitutive subunits in European seabass, 87.6% of 435 gene-pair correlations were positive across 18 developmental stages (median Spearman *rho* = 0.74), and all 435 were positive across four adult tissues (median *rho* = 0.8). Among the constitutive subunits in gilthead seabream, 96.8% of 528 pairs were positive across the three post-hatching stages (median *rho* = 0.7), and 99.2% were positive across four adult tissues (median *rho* = 0.9). Correlations involving activators, specialized core components, and assembly or other regulators were more heterogeneous. Because the gilthead seabream developmental matrix had only three profiles and each tissue matrix had only four, the coefficients are discrete and descriptive; no *p*-values, multiple-testing correction, or inferential correlation threshold was applied. Overall, the patterns are consistent with coordinated transcript abundance among constitutive proteasome subunits, not direct evidence of functional coordination or protein stoichiometry.

### 3.4. Summary of Paralog Expression Patterns and Coexpression

Across the developmental and adult-tissue datasets, retained UPS, calpain, and cathepsin paralogs displayed diverse transcript-abundance profiles. In both species, at least one copy of each examined multicopy family was detected in the sampled developmental stages or tissues. Some paralogs showed broadly comparable abundance, whereas others displayed copy-asymmetric or stage- or tissue-associated patterns. Thus, duplicated proteolytic genes differed not only in copy number and inferred duplication history but also in their expression profiles, with the specific patterns varying among systems and between species.

## 4. Discussion

### 4.1. Evolutionary Diversification of Calpains in Teleosts

**Figure 14:**
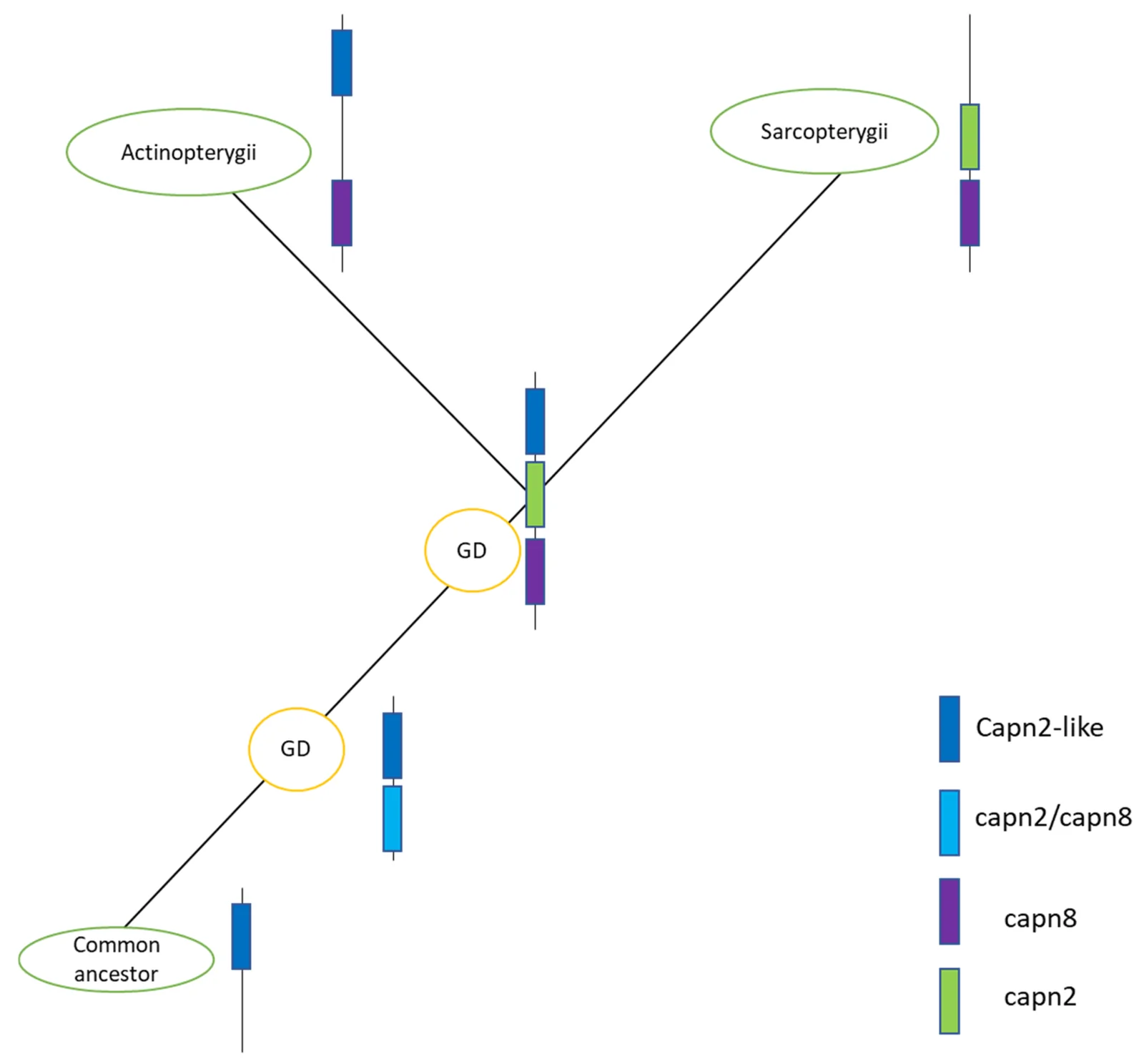
Proposed evolutionary scenario for the origin of *capn2* and *capn8*. The *capn2*-like gene is shown in dark blue, the *capn2*/*capn8* ancestral gene in light blue, *capn2* in green, and *capn8* in purple. Orange circles indicate gene duplication events.

Calpains have central roles in vertebrate cellular physiology, including cytoskeletal remodeling [63,64], cell-cycle regulation [64], and apoptosis [18]. However, information on their evolutionary history and functional diversification in teleosts remains limited. Previous studies have mainly focused on *CAPN1* and *CAPN2* because of their broad conservation across vertebrates [65]. In rainbow trout (*Oncorhynchus mykiss*), orthologs of mammalian *capn1* and *capn2* display similar sequence and structural characteristics [66], and biochemical analyses of *capn2* showed Ca^2+^ requirements comparable to those of other vertebrates and the ability to form a heterodimer with the small regulatory subunit of conventional calpains [67]. In the present study, the calpain family was examined in gilthead seabream and European seabass through phylogenetic, syntenic, and expression patterns analyses. The family-level phylogeny highlighted the close relationship between the *capn2* and *capn8* clades. This is consistent with previous evidence suggesting that teleost and tetrapod *capn2* genes are not direct one-to-one orthologs, despite their high sequence and structural conservation [33]. Under this evolutionary hypothesis, a *capn2*-like gene was present before the divergence of Sarcopterygii and Actinopterygii and was duplicated early in vertebrate evolution, probably during the first round of vertebrate whole-genome duplication. One resulting lineage subsequently gave rise to the *capn2*/*capn8* ancestor, and *capn2* and *capn8* are considered to have derived from a tandem duplication that occurred near the base of vertebrates and before the split between Sarcopterygii and Actinopterygii [68]. After the divergence of these lineages, teleosts appear to have lost the tetrapod-like *capn2* gene, whereas Sarcopterygii lost the *capn2*-like gene (Figure 14).

The complete calpain phylogeny also recovered a close evolutionary relationship among *capn11*, *capn1*, *capn2*, and *capn8*. Rather than indicating a simple linear origin, this topology places *capn11* in a position consistent with an early-diverging member of this clade. This interpretation is also supported by the fact that *capn11*, like *capn1* and *capn2*, associates with the small regulatory subunit *capns1* and shares similar properties [34,69]. In teleosts, *capn11* has also been associated with the degradation of a broad range of substrates [70]. Together with the synteny data, which placed these genes in neighboring genomic regions in gilthead seabream and European seabass (Supplementary Table S28), these findings support a close relationship among *capn11*, *capn2*, and *capn8*, in agreement with previous studies [33,65,70]. Previous studies have also associated *capn1* with this gene cluster. Although *capn1* was not located within the corresponding syntenic region in either species examined here, previous findings support its evolutionary association with these genes. Its present-day genomic separation may reflect chromosomal rearrangements that disrupted the ancestral syntenic organization during the evolution of these two lineages. Several calpain genes were retained as multiple copies, particularly in gilthead seabream, including five copies of *capn2* and three copies each of *capn8*, *capn11*, and *capn14*. Based on the phylogenetic data, some *capn2*, *capn11*, and *capn14* copies in gilthead seabream appear to derive from duplications that occurred within the gilthead seabream lineage. By contrast, two of the three *capn8* paralogs appear to derive from a duplication within *Percomorpha*, as sequences from *Seriola dumerilii* were also recovered in the same clades. These patterns indicate that the present-day conventional calpain repertoire is the result of multiple evolutionary events rather than a single duplication episode. The non-conventional calpains formed a distinct subgroup, with *capn15* occupying the most distant position within this set. This finding was consistent with the structure of *capn15*, which showed the most divergent architecture among the calpains, lacking the C2 domain and containing a SOL domain at the C-terminal of the CysPC catalytic domain [14]. Furthermore, the broader taxonomic analysis indicated that the calpain repertoire expanded after the divergence of vertebrates from the urochordate lineage. In *Ciona intestinalis*, a urochordate that diverged before the vertebrate whole-genome duplications [71], five calpain-family genes were identified, suggesting a more restricted repertoire than in vertebrates. Each of these genes clustered with one of the five major calpain subgroups described by Sorimachi and colleagues [69], indicating that vertebrate whole-genome duplications contributed to the subsequent expansion of the calpain family (Supplementary Figure S24).

Although, the observed pattern is broadly compatible with the general expectation, as described by Ohno, that genome duplication can expand gene families (“1-2-4-8” theory) [28] the limited taxon set and annotation completeness preclude a causal inference. A recent review of insect embryogenesis placed calpains within evolving signaling networks governing cell-cycle progression and dorsoventral patterning and argued that gene-family expansion, modular rearrangement, and regulatory recruitment can promote functional diversification [72]. At a broader phylogenetic scale, Fletcher et al. proposed a testable cross-eukaryotic link between calpains, microtubule organization, and cytokinesis, while explicitly noting that this remains hypothetical [73].

### 4.2. Evolutionary Trajectory of Cathepsins in Teleosts

The evolutionary history of cathepsins appears also complex. Cathepsins are multifunctional enzymes found in a wide range of species [15], including *Caenorhabditis elegans* [74,75], while related proteases are also present in other invertebrates [76]. Whole-genome duplication, local gene duplication, and subsequent diversification under different evolutionary pressures have contributed to the emergence of distinct cathepsin groups with specialized functions [77]. The family-level cathepsin tree placed *ctsa* and *ctsd* as more distantly related to the cysteine cathepsins. Within the cysteine cathepsins, two major clades were recovered: one including *ctss*, *ctsk*, *ctsl*, *ctsh*, and *ctsf*, and another including *ctsc*, *ctsz*, *ctsb*, and *ctso*. The available phylogenetic evidence suggests that these cathepsin lineages diverged from *ctsb*- and *ctsl*-related groups, which separated early in eukaryotic evolution. In vertebrates, duplication events within the *ctsl* lineage contributed to the emergence of *ctss* and *ctsk*, as well as to a rodent-specific group containing multiple *ctsl* copies. The monophyletic organization of this rodent *ctsl* group suggests divergence within the rodent lineage from mammalian L-cathepsins [78]. A notable feature of *ctss* evolution was the presence of tandemly arranged paralogs. In gilthead seabream, five *ctss* copies were located within a single genomic region, with one member also showing WGD/segmental evidence, whereas *X. maculatus* contained two tandem pairs at two separate genomic locations. Two non-mutually exclusive evolutionary scenarios could explain these arrangements. First, a WGD-derived copy may have undergone independent lineage-specific tandem expansion, potentially facilitated by local genomic instability and mechanisms such as unequal crossing-over, non-allelic homologous recombination, or replication-associated duplication. The repeated retention of *ctss* paralogs in these regions may indicate that additional copies were tolerated or potentially favored, although copy number alone cannot demonstrate selection. Alternatively, the common ancestor of these lineages may already have possessed a tandemly duplicated *ctss* arrangement. Subsequent WGD, chromosomal rearrangement, differential gene loss, and additional local duplication could then have produced the distinct present-day configurations observed in the two species. Similar expansions of other cathepsin genes have been reported in zebrafish and mammalian lineages such as rodents [35]. Broader taxonomic sampling and comparative analysis of flanking synteny and duplication breakpoints would be required to distinguish between ancestral inheritance and recurrent independent expansion. Sequence similarities of cathepsins in gilthead seabream and European seabass showed high conservation across several regions, consistent with conservation of the structural features required for catalytic activity.

At the same time, the signal-peptide region was highly polymorphic. Although signal peptides share general features, such as a hydrophobic core and a cleavage site, they may vary in length and amino acid composition [79]. Variation in signal-peptide sequences could potentially influence protein targeting or secretion, although these effects were not evaluated experimentally in the present study [80]. In contrast, the universal conservation of active-site residues indicates strong evolutionary constraint on positions required for enzymatic activity [81,82]. A particularly close parallel is provided by common carp, in which nine *ctsl* paralogs were traced to ancient vertebrate, teleost-specific, and carp-specific whole-genome duplications, together with a subsequent tandem duplication [36]. The coexistence of conserved synteny with lineage-specific retention and loss supports the interpretation that teleost cathepsin repertoires are assembled through successive layers of duplication and rediploidization rather than through a single whole-genome duplication event.

### 4.3. Evolutionary Trajectory of the Ubiquitin–Proteasome System in Teleosts

The ubiquitin–proteasome system (UPS) is one of the major intracellular proteolytic pathways responsible for selective protein degradation and proteome quality control. In contrast to calpains and cathepsins, which are organized as protease families with members that may differ in regulation, localization, tissue distribution, and substrate context, the UPS functions through a highly organized multi-subunit proteolytic machinery. Ubiquitinated substrates are recognized and degraded by the 26S proteasome, which consists of the 20S catalytic core particle and regulatory particles. Consistent with this organization, the family-level phylogenetic tree separated UPS components into distinct clusters corresponding to their functional subunit classes. This organization requires coordinated assembly and balanced expression of multiple interacting subunits [83]. Compared with calpains and cathepsins, the literature on UPS evolution and paralog-specific expression in fish remains relatively scarce. Available studies include systematic genomic and expression analyses of proteasome subunit genes in grass carp, in which proteasome genes were identified, characterized, and shown to respond transcriptionally to environmental stress conditions [37]. However, comparable studies in marine teleosts and analyses focusing specifically on retained UPS paralogs following the Ts3R remain limited. In the present study, 51 human genes encoding UPS subunits were used as reference sequences, whereas 61 genes were identified in gilthead seabream and 53 in European seabass. Although both teleost species showed a modest expansion relative to humans, most UPS genes were recovered as single-copy genes across the species examined. This pattern differs from the broader paralog retention observed for calpains and cathepsins and is consistent with the conserved organization of the proteasome as a multi-subunit complex. It also agrees with a previous study in grass carp that reported a similarly conserved gene repertoire [37]. According to the gene balance hypothesis, genes encoding components of macromolecular complexes may be strongly affected by dosage and stoichiometric constraints, as imbalance among interacting subunits can affect complex assembly and function [84]. Although it should be noted that the predominance of single-copy UPS genes and limited retention of selected duplicates is compatible with that hypothesis, but this study did not measure gene dosage, subunit stoichiometry, protein abundance, or correlated copy retention; gene balance therefore remains an interpretation rather than a demonstrated mechanism. Nevertheless, several UPS gene families were retained as duplicated copies in the two teleost species. In gilthead seabream, duplicated UPS genes included *psma4*, *psma6*, *psmb4*, *psmb11*, *psmd4*, *psmd11*, *psmd13*, *psme3*, and *psme4*, whereas in European seabass duplicated genes included *psma4*, *psma6*, *psmb11*, *psmd4*, *psmd11*, and *psme4*. Integration of phylogenetic and syntenic evidence indicated that most retained UPS duplicates were compatible with an origin in the teleost-specific whole-genome duplication, suggesting that Ts3R contributed to shaping the current UPS repertoire in both species. However, this retention was limited to selected components rather than affecting the UPS uniformly. Among the duplicated UPS genes, *psmb11* is particularly relevant because comparative genomic work has shown that teleost fishes possess two apparently functional *PSMB11* genes, designated *PSMB11a* and *PSMB11b* [85]. In the present analysis, the retention of *psmb11* paralogs as transcriptionally active identifies this gene as a candidate for further functional investigation. However, the presence of duplicated copies should not by itself be interpreted as evidence of tissue- or immune-specific specialization without additional functional validation. The duplicated UPS genes also included proteasome core subunits, regulatory-particle subunits, and proteasome activator-related genes. This indicates that retained duplicates were not restricted to a single structural part of the UPS. In addition, the gilthead seabream-specific duplications of *psmb4* and *psmd13* suggest that local duplication mechanisms may have contributed to limited species-specific expansion of the UPS repertoire. Compared with the broader duplication patterns observed in calpains and cathepsins, however, this local expansion appears more restricted. Overall, the UPS appears to have followed a more constrained evolutionary trajectory than calpains and cathepsins. Its modest expansion in gilthead seabream and European seabass, together with the predominance of single-copy genes, suggests relatively strong conservation of the proteasome gene copies and expression across the two focal species. At the same time, the retention of selected duplicated genes, mostly compatible with a 3R origin, indicates that teleost genome duplication left a detectable but limited signal in the UPS.

### 4.4. Developmental and Tissue Expression Patterns

The expression data provide an additional layer for interpreting the fate of retained calpain and cathepsin paralogs although descriptive due to lack of biological replicates in the post-hatching samples. Calpains were first isolated from rat brain in 1964 and later from chicken in 1978, supporting their evolutionary conservation across vertebrates [86,87]. Nevertheless, studies on calpain expression and physiological function in teleosts remain limited, and many available studies focus on post-mortem muscle degradation in commercially important fish species [88,89,90,91]. In mammals, six of the fifteen calpain genes show tissue-specific expression: *CAPN3* in skeletal muscle, *CAPN6* in placenta and embryonic muscle cells, *CAPN8* and *CAPN9* in the gastrointestinal tract, *CAPN11* in testes, and *CAPN12* in hair follicles [69]. In fish, tissue-expression studies have focused mainly on conventional calpains, particularly *capn1* and *capn2*. Broad tissue expression of *capn2* has been reported in trout [66], Atlantic halibut [33], and zebrafish [92]. Calpain expression has also been associated with nutritional status, since several fish species use skeletal-muscle proteins as energy reserves during prolonged food deprivation [93]. In *Pagrus major*, *capn3* was highly expressed in muscle and skin of healthy individuals, whereas pathogen challenge increased *capn3* expression in the liver, suggesting a possible involvement in host defense [94,95]. Expression data for non-conventional calpains remain scarce, although *capn5* is highly expressed in the retina of adult zebrafish and its expression is increased in retinal cones in a model of chronic photoreceptor degeneration and regeneration [96]. The developmental expression patterns observed here are consistent with roles for calpains in embryogenesis and tissue formation. In zebrafish, *capn5* is highly expressed during development and participates in embryonic brain, optic vesicle, and retinal photoreceptor development [96], while *capn15* has also been implicated in visual-system development [97]. Calpains also contribute to myogenesis, including migration and differentiation of myogenic progenitor cells and fusion of white-muscle myogenic cells into small myoblasts [98]. In zebrafish embryos, *capn1*, *capn2*, and *capns1* expression patterns include maternally derived transcripts, followed by stage-specific expression after zygotic-genome activation [99], in agreement with the early expression patterns detected in European seabass. Calpains participate in cell division, cell differentiation, blastula formation, gastrulation, cell migration, organogenesis, apoptosis, and autophagy [99,100], thereby contributing to the maintenance of cellular homeostasis during development [13].

Cathepsins also showed expression patterns consistent with their broad roles in cellular physiology. They are integral components of intracellular protein degradation across diverse organisms, from viruses to vertebrates [101], are mainly localized in lysosomes [102], and contribute to protein recycling, antigen presentation, pathogen defense [77], apoptosis [103], extracellular matrix remodeling [104], bone remodeling and resorption [15,105], hormone processing [106], sperm maturation, and embryonic development [100]. In mammals, *ctsb* and *ctsl* participate in extracellular type I collagen degradation in bone and in the processing of hormones and antigenic proteins [107], whereas in arthropods *ctsl* has a role in molting and chitin degradation [101]. During vertebrate reproduction and early development, *ctsb*, *ctsl*, and *ctsd* have been associated with yolk formation and modification [108,109]. During oocyte development, vitellogenin is internalized by endocytosis and degraded into smaller peptides that support embryonic energy demands, a process facilitated by *ctsd* [108]. The high abundance levels of *ctsd* paralogs during European seabass development, together with the early abundance of, supported by literature, maternally supplied transcripts, is consistent with this role. Gilthead seabream and European seabass produce highly hydrated eggs [110], and additional proteolysis during oocyte maturation generates small peptides and free amino acids that contribute to the osmotic gradient required for water uptake [111]. In teleosts, several cathepsins participate in maturation-associated yolk proteolysis and are activated by pH changes in the yolk during embryonic and larval development [112]. Most cathepsin genes showed high transcript abundance from early European seabass embryogenesis, with at least one paralog expressed for each gene, and this expression remained high after zygotic-genome activation, supporting a role in embryonic development [113]. However, delayed or low expression of specific paralogs, such as *ctsb.2*, *ctsl.2*, and *ctss.2*, relative to their corresponding copies, suggests that cathepsin expression is controlled by paralog-specific regulatory mechanisms. Similar developmental patterns have been reported for *ctsl* paralogs in zebrafish and killifish, where one copy is detected from the morula stage and increases during gastrulation, supporting a role in yolk resorption [111,114]. Transcripts of other cathepsins, including *ctss* and *ctsc*, have also been detected in the yolk syncytial layer of zebrafish embryos, suggesting that yolk degradation during embryogenesis requires the combined action of different proteases [115,116]. In adult tissues, cathepsin expression may also be linked to energy redistribution and reproduction. Lysosomal enzymes contribute to the reallocation of energy toward reproduction [103], and gonadal maturation is often accompanied by peripheral muscle proteolysis, particularly in salmon [100]. This process involves activation of the lysosomal system, especially cathepsins D and L, which degrade muscle proteins and redirect the released energy toward reproductive processes [100]. The common-carp study likewise reported pronounced tissue- and cell-specific partitioning among *ctsl* paralogs, together with a conserved global fold but variation in substrate-binding loops [36]. This direct teleost comparison strengthens the interpretation that conserved catalytic capacity can coexist with regulatory and potentially functional divergence; however, expression partitioning alone remains insufficient to assign subfunctionalization or neofunctionalization.

The expression pattern analysis showed that most UPS genes were broadly expressed, whereas duplicated families displayed modest differences in transcript abundance across stages or tissues. This pattern is consistent with the essential role of the UPS in cellular proteostasis, but it also suggests that selected duplicated UPS paralogs may have undergone some degree of regulatory differentiation. The coexpression analysis provides an additional, although indirect, line of evidence for dosage-related constraints in the UPS. The predominantly positive correlations among constitutive 20S and 19S subunits across developmental stages and tissues are consistent with coordinated transcript abundance among components that must assemble in appropriate proportions. Sundararajan et al. showed that loss of correlated proteasomal subunit expression was associated with stoichiometric imbalance, reduced formation of double-capped proteasomes, and accumulation of free 20S cores, demonstrating that disruption of subunit coordination can affect proteasome assembly and function [117]. However, because our analysis was restricted to transcript abundance and did not measure protein abundance, subunit stoichiometry, or proteasome assembly, these findings provide supportive rather than direct evidence of dosage sensitivity. Accordingly, expression differences among UPS paralogs should be interpreted more cautiously than those observed among calpain or cathepsin paralogs.

### 4.5. Fate of Retained Paralogs

The combined developmental and tissue-specific expression pattern analyses revealed differentiated regulatory patterns among calpain and cathepsin paralogs in gilthead seabream and European seabass, although these patterns differed among the UPS, calpain, and cathepsin systems. In humans, six of the fifteen calpain genes show tissue-specific expression patterns, whereas the remaining genes are more broadly expressed [69]. In both teleost species examined here, each calpain gene family was represented by at least one expressed copy in the tissues analyzed. Similarly, human *CTSB*, *CTSH*, and *CTSL* are broadly expressed, whereas *CTSS* shows tissue-specific expression [35,118]. In gilthead seabream and European seabass, each cathepsin gene family was also represented by at least one expressed copy in the tissues examined. For the UPS, most genes were broadly expressed, consistent with the conserved and essential role of the proteasome in cellular proteostasis. Gene duplication, and especially whole-genome duplication, provides redundant genetic material that may facilitate functional divergence [21]. However, the fate of duplicated genes is shaped by natural selection, gene dosage effects [119], and the balance between the benefits of redundancy and the costs of maintaining duplicated genomic regions [24]. Initially redundant paralogs may persist, diverge, or be lost [24,120], and gene loss is generally more common than long-term retention of functionally redundant copies [21,23,24,121,122]. Thus, single-copy calpain, cathepsin, and UPS genes may reflect paralog loss during rediploidization after whole-genome duplication [123]. When duplicated genes are retained, mutations may accumulate in coding or regulatory regions, leading either to changes in protein structure or to divergence in spatiotemporal regulation [23,24,124,125,126,127,128].

In the present study, calpain, cathepsin, and UPS paralogs showed conserved catalytic regions in their respective sequence, suggesting retention of catalytic potential. However, conserved catalytic motifs do not exclude divergence in regulation, localization, substrate preference, interaction partners, or activity.

The observed differences in transcripts abundance, in the number of paralogs expressed at each developmental stage, and in tissue-specific expression patterns therefore highlight candidate paralogs that may have undergone regulatory divergence. Such patterns are consistent with possible regulatory subfunctionalization, while regulatory neofunctionalization cannot be excluded, particularly if expression differences reflect changes in cis-regulatory elements or paralog-specific regulatory responses. Nevertheless, the present data cannot distinguish among these possibilities. Expression patterns alone cannot reconstruct ancestral states and therefore cannot demonstrate whether the observed divergence resulted from subfunctionalization or neofunctionalization. Rather, they provide a useful framework for identifying paralogs of particular interest for future functional investigation.

Several characteristics of the datasets should also be considered when interpreting these expression patterns. For the post-hatching stages, one pooled library was available per developmental stage, precluding statistical testing of stage-specific effects. Embryonic transcriptomic data were available only for European seabass, while the adult-tissue datasets originated from heterogeneous publicly available studies. Although batch correction was applied to improve comparability among these datasets, some residual variation associated with sampling, library preparation, or sequencing may remain. The results should therefore be interpreted as comparative and hypothesis-generating. Nevertheless, the combined evolutionary, and expression analyses provide a biologically informative framework for investigating paralog retention and divergence, which can be further examined through replicated expression analyses and protein-level, biochemical, and functional validation.

Taken together, the UPS, calpains, and cathepsins appear to have followed different evolutionary trajectories after genome duplication. The UPS showed a more constrained pattern, with most genes retained as single copies and only selected duplicated components maintained after the teleost-specific whole-genome duplication. The coordinated multi-subunit organization of the proteasome requires appropriate stoichiometry among core, regulatory, and activator components for its assembly and function. Retained UPS paralogs may therefore reflect dosage balance, coordinated retention, or subtle regulatory specialization rather than extensive divergence.

By contrast, calpains and cathepsins exhibited broader variation in copy number, duplication origin, and expression profiles. Calpain paralogs generally retained conserved catalytic domains but displayed developmental- and tissue-specific expression differences consistent with possible regulatory divergence. Cathepsins showed a similar pattern, with broadly conserved active-site residues and paralog-specific differences in transcript abundance, particularly among duplicated cysteine cathepsins. Because calpains and cathepsins function as individual proteases and can differ in their regulatory properties, tissue distribution, and developmental timing, their duplicates are less constrained by the stoichiometric requirements of multicomponent complexes and may therefore undergo regulatory subfunctionalization or neofunctionalization.

Overall, genome duplication did not affect intracellular proteolysis uniformly in teleosts; the evolutionary trajectory of each system appears to have been shaped by its molecular architecture, cellular role, and dependence on stoichiometric interactions. Although the genomic and expression data identify candidate paralogs showing possible regulatory divergence, direct functional studies will be required to establish the precise biological roles of individual calpain, cathepsin, and UPS paralogs.

## 5. Conclusions

The comparative analysis presented here suggests that the major intracellular proteolytic systems of teleosts followed partly distinct evolutionary trajectories. In gilthead seabream and European seabass, the UPS, calpain, and cathepsin repertoires differed in gene number, inferred duplication history, and expression patterns. The UPS exhibited a more constrained evolutionary pattern, characterized by lower paralog retention and comparatively modest diversification, with most genes represented by single copies and only selected duplicated components retained after the teleost-specific whole-genome duplication. By contrast, calpains and cathepsins showed broader paralog retention, mixed duplication origins, and more pronounced developmental and tissue-specific expression patterns. These patterns are consistent with an association between the molecular organization of each proteolytic system and the retention and expression divergence of duplicated genes. In the multi-subunit UPS constraints related to coordinated subunit function and dosage balance may contribute to the comparatively limited retention of duplicated components whereas calpain and cathepsin families may have experienced greater regulatory flexibility. Overall, the integration of phylogenetic, syntenic, and transcriptomic evidence identifies retained proteolytic paralogs with divergent expression patterns that may reflect regulatory divergence, potentially consistent with possible regulatory subfunctionalization and/or neofunctionalization in teleost fishes.

## Figures and Tables

**Table 1:** Summary of UPS, calpain and cathepsin gene repertoires identified in human, gilthead seabream and European seabass.

Gene Family/System	Human Reference Genes	*Sparus aurata*	*Dicentrarchus labrax*
UPS	51	61 (9)	53 (6)
Calpains	16	29 (9)	22 (8)
Cathepsins	15	23 (6)	17 (5)

Table note: Numbers outside parentheses indicate the total number of genes, whereas numbers in parentheses indicate the number of multicopy gene families. In *S. aurata*, the multicopy UPS families were *psma4*, *psma6*, *psmb4*, *psmb11*, *psmd4*, *psmd11*, *psmd13*, *psme3*, and *psme4*; the multicopy calpain families were *capn2*, *capn3*, *capn5*, *capn6*, *capn8*, *capn11*, *capn14*, *capn15*, and *capns1*; and the multicopy cathepsin families were *ctsb*, *ctsd*, *ctsh*, *ctsl*, *ctss*, and *ctsz*. In *D. labrax*, the multicopy UPS families were *psma4*, *psma6*, *psmb11*, *psmd4*, *psmd11*, and *psme4*; the multicopy calpain families were *capn2*, *capn3*, *capn5*, *capn6*, *capn11*, *capn14*, *capn15*, and *capns1*; and the multicopy cathepsin families were *ctsb*, *ctsd*, *ctsl*, *ctss*, and *ctsz*.

**Table 2:** MCScanX duplicate-gene classifications in European seabass and gilthead seabream. Assignments are algorithmic categories; differences between species may reflect biological variation or differences in assembly, annotation, and detectable collinearity. The final column reports the duplication event or events inferred from phylogenetic topology and taxonomic distribution. A plus sign indicates that multiple duplication events contributed to the expansion of the respective gene family. “1R/2R” indicates an early vertebrate duplication whereas “Ts3R” indicates the teleost-specific third round of whole-genome duplication. “Lineage-specific” denotes a subsequent duplication restricted to a particular lineage or species, and “Unresolved” indicates that the phylogenetic evidence was insufficient to assign a duplication origin. A dash indicates that the respective gene family was not duplicated in that species.

Gene	*S. aurata*	*D. labrax*	Phylogenetic Inference
*capn2*	WGD/segmental & proximal	WGD/segmental	1R/2R + Ts3R + Lineage specific
*capn3*	dispersed	WGD/segmental	Ts3R
*capn5*	WGD/segmental	dispersed	Ts3R
*capn6*	dispersed	dispersed	Ts3R
*capn8*	WGD/segmental & tandem	-	1R/2R + Lineage specific
*capn11*	WGD/segmental & proximal	dispersed	Ts3R
*capn14*	dispersed & tandem	dispersed	Ts3R + Lineage specific
*capn15*	dispersed	dispersed	Ts3R
*capns1*	dispersed	dispersed	Ts3R
*ctsb*	dispersed	dispersed	1R/2R
*ctsd*	WGD/segmental & tandem	dispersed	1R/2R + Lineage specific
*ctsh*	WGD/segmental	-	Ts3R
*ctsl*	dispersed	dispersed	Unresolved
*ctss*	WGD/segmental & tandem	dispersed	Ts3R + Lineage specific
*ctsz*	dispersed	dispersed	1R/2R + Ts3R + Lineage specific
*psma4*	dispersed	WGD/Segmental	Ts3R
*psma6*	WGD/Segmental	WGD/Segmental	Ts3R
*psmb4*	tandem	-	Lineage specific
*psmb11*	WGD/Segmental	dispersed	Ts3R
*psmd4*	dispersed	WGD/Segmental	Ts3R
*psmd11*	WGD/Segmental	WGD/Segmental	Ts3R
*psmd13*	Proximal	-	Lineage specific
*psme3*	WGD/Segmental	-	Ts3R
*psme4*	WGD/Segmental	WGD/Segmental	Ts3R

## Data Availability

All sequencing data used were retrieved from the Sequence Read Archive (SRA), and the corresponding accession numbers are provided in the main body of the manuscript and in Supplementary Table S3.

## Supplementary Materials

The following supporting information can be downloaded at: https://www.mdpi.com/article/10.3390/biology15191755/s1. Figure S1. Maximum-likelihood (ML) phylogenetic tree of the *capn2* protein. The orange circle indicates the duplication node consistent with the first or second round of whole-genome duplication. The red circle indicates the duplication node consistent with the third round of whole-genome duplication, while the green circle indicates the duplication node specific to the gilthead seabream lineage. The substitution model used was T92+G+I. Numbers at the nodes represent bootstrap values; Figure S2. Maximum-likelihood (ML) phylogenetic tree of the *capn3* protein. The orange circle indicates the duplication node consistent with the third round of whole-genome duplication. The substitution model used was GTR+G+I. Numbers at the nodes represent bootstrap values; Figure S3. Maximum-likelihood (ML) phylogenetic tree of the *capn5* protein. The red circle indicates the duplication node consistent with the third round of whole-genome duplication. The substitution model used was HKY+G. Numbers at the nodes represent bootstrap values; Figure S4. Maximum-likelihood (ML) phylogenetic tree of the *capn6* protein. The red circle indicates the duplication node consistent with the third round of whole-genome duplication. The substitution model used was T92+G+I. Numbers at the nodes represent bootstrap values; Figure S5. Maximum-likelihood (ML) phylogenetic tree of the *capn8* protein. The orange circle indicates the duplication node consistent with the first or second round of whole-genome duplication, while the green circle indicates the duplication node specific to the gilthead seabream lineage. The substitution model used was K2+G+I. Numbers at the nodes represent bootstrap values; Figure S6. Maximum-likelihood (ML) phylogenetic tree of the *capn11* protein. The red circle indicates the duplication node consistent with the third round of whole-genome duplication. The green circle indicates the duplication node specific to the gilthead seabream lineage. The substitution model used was K2+G. Numbers at the nodes represent bootstrap values; Figure S7. Maximum-likelihood (ML) phylogenetic tree of the *capn14* protein. The red circle indicates the duplication node consistent with the third round of whole-genome duplication. The green circle indicates the duplication node specific to the *Percomorpha* lineage. The substitution model used was K2+G. Numbers at the nodes represent bootstrap values; Figure S8. Maximum-likelihood (ML) phylogenetic tree of the *capn15* protein. The red circle indicates the duplication node consistent with the third round of whole-genome duplication. The substitution model used was T92+G. Numbers at the nodes represent bootstrap values; Figure S9. Maximum-likelihood (ML) phylogenetic tree of the *capns1* protein. The red circle indicates the duplication node consistent with the third round of whole-genome duplication. The substitution model used was JTT+G. Numbers at the nodes represent bootstrap values; Figure S10. Maximum-likelihood (ML) phylogenetic tree of the *ctsb* protein. The orange circle indicates the duplication node consistent with the second round of whole-genome duplication. The substitution model used was JTT+G+I. Numbers at the nodes represent bootstrap values; Figure S11. Maximum-likelihood (ML) phylogenetic tree of the *ctsd* protein. The orange circle indicates the duplication node consistent with the second round of whole-genome duplication. The substitution model used was JTT+G+I. Numbers at the nodes represent bootstrap values; Figure S12. Maximum-likelihood (ML) phylogenetic tree of the *ctsh* protein. The red circle indicates the duplication node consistent with the TS-WGD. The substitution model used was JTT+G+I. Numbers at the nodes represent bootstrap values; Figure S13. Maximum-likelihood (ML) phylogenetic tree of the *ctss* protein. The red circle indicates the duplication node consistent with the TS-WGD, while the green circle indicates a single lineage duplication node. The substitution model used was JTT+G. Numbers at the nodes represent bootstrap values; Figure S14. Maximum-likelihood (ML) phylogenetic tree of the *ctsz* protein. The red circle indicates the duplication node consistent with the TS-WGD, while the orange circle indicates the duplication node consistent with the first or second round of whole-genome duplication. The substitution model used was JTT+G+I. Numbers at the nodes represent bootstrap values; Figure S15. Maximum-likelihood (ML) phylogenetic tree of the *psmd11* gene. The red circle indicates the duplication node consistent with the TS-WGD. The substitution model used was GTR+G+I. Numbers at the nodes represent bootstrap values; Figure S16. Maximum-likelihood (ML) phylogenetic tree of the *psmd4* gene. The red circle indicates the duplication node consistent with the TS-WGD. The substitution model used was GTR+G+I. Numbers at the nodes represent bootstrap values; Figure S17. Maximum-likelihood (ML) phylogenetic tree of the *psme3* gene. The red circle indicates the duplication node consistent with the TS-WGD. The substitution model used was GTR+G+I. Numbers at the nodes represent bootstrap values; Figure S18. Maximum-likelihood (ML) phylogenetic tree of the *psmb11* gene. The red circle indicates the duplication node consistent with the TS-WGD. The substitution model used was GTR+G+I. Numbers at the nodes represent bootstrap values; Figure S19. Maximum-likelihood (ML) phylogenetic tree of the *psme4* gene. The red circle indicates the duplication node consistent with the TS-WGD. The substitution model used was GTR+G+I. Numbers at the nodes represent bootstrap values; Figure S20. Maximum-likelihood (ML) phylogenetic tree of the *psma4* gene. The red circle indicates the duplication node consistent with the TS-WGD. The substitution model used was GTR+G+I. Numbers at the nodes represent bootstrap values; Figure S21. Maximum-likelihood (ML) phylogenetic tree of the *psmb4* gene. The green circle indicates a single lineage duplication node. The substitution model used was GTR+G+I. Numbers at the nodes represent bootstrap values; Figure S22. Maximum-likelihood (ML) phylogenetic tree of the *psmd13* gene. The green circle indicates a single lineage duplication node. The substitution model used was GTR+G+I. Numbers at the nodes represent bootstrap values; Figure S23. Maximum-likelihood (ML) phylogenetic tree of the calpain family. The underlined *Ciona intestinalis* sequences indicate the five urochordate calpain-family genes identified in the analysis. Their clustering within the major calpain subgroups suggests that these lineages were already present before vertebrate whole-genome duplications, whereas the broader expansion of the calpain repertoire occurred later in vertebrates; Figure S24. Spearman correlation heatmap of UPS subunit expression during European seabass development. Pairwise Spearman correlation coefficients were calculated across the available developmental stages. Where numbered paralogs were present, their TPM values were summed before log_2_(TPM + 0.25) transformation. Genes with invariant expression profiles were excluded because their correlations could not be calculated. The colour scale ranges from −1 (blue) to +1 (red), while the upper and right annotation bars indicate the functional classification of each subunit. Genes were ordered according to their functional modules without hierarchical clustering; Figure S25. Spearman correlation heatmap of UPS subunit expression during gilthead seabream development. Pairwise Spearman correlation coefficients were calculated across the available developmental stages. Where numbered paralogs were present, their TPM values were summed before log_2_(TPM + 0.25) transformation. Genes with invariant expression profiles were excluded because their correlations could not be calculated. The colour scale ranges from −1 (blue) to +1 (red), while the upper and right annotation bars indicate the functional classification of each subunit. Genes were ordered according to their functional modules without hierarchical clustering; Figure S26. Spearman correlation heatmap of UPS subunit expression across European seabass adult tissues. Pairwise Spearman correlation coefficients were calculated between genes across the four adult-tissue profiles. Where numbered paralogs were present, their TPM values were summed before log_2_(TPM + 0.25) transformation. The matrix is descriptive; no *p*-value or multiple-testing threshold was applied; Figure S27. Spearman correlation heatmap of UPS subunit expression across gilthead seabream adult tissues. Pairwise Spearman correlation coefficients were calculated between genes across the four adult-tissue profiles. Where numbered paralogs were present, their TPM values were summed before log_2_(TPM + 0.25) transformation. The matrix is descriptive; no *p*-value or multiple-testing threshold was applied. Table S1. Human gene family members retrieved from the HGNC (HUGO Gene Nomenclature Committee) database with the corresponding Ensembl IDs; Table S2. Species selected for the phylogenetic analysis and the accession numbers of each species genome. *: Stickleback Tetraodon and Lamprey no genome accession number was available; therefore, the corresponding Ensembl assembly ID is reported; Table S3. Public RNAseq data from various tissues in both *S. aurata* and *D. labrax*. Missing tissue for each species is reported with “-”; Table S4. Correspondence between standardized gene names and Ensembl IDs in gilthead seabream and European seabass; Table S5. Table showing the correspondence between Ensembl IDs and the names used in the *capn2* phylogenetic tree; Table S6. Table showing the correspondence between Ensembl IDs and the names used in the *capn3* phylogenetic tree; Table S7. Table showing the correspondence between Ensembl IDs and the names used in the *capn5* phylogenetic tree; Table S8. Table showing the correspondence between Ensembl IDs and the names used in the *capn6* phylogenetic tree; Table S9. Table showing the correspondence between Ensembl IDs and the names used in the *capn8* phylogenetic tree; Table S10. Table showing the correspondence between Ensembl IDs and the names used in the *capn11* phylogenetic tree; Table S11. Table showing the correspondence between Ensembl IDs and the names used in the *capn14* phylogenetic tree; Table S12. Table showing the correspondence between Ensembl IDs and the names used in the *capn15* phylogenetic tree; Table S13. Table showing the correspondence between Ensembl IDs and the names used in the *capns1* phylogenetic tree; Table S14. Table showing the correspondence between Ensembl IDs and the names used in the *ctsb* phylogenetic tree; Table S15. Table showing the correspondence between Ensembl IDs and the names used in the *ctsd* phylogenetic tree; Table S16. Table showing the correspondence between Ensembl IDs and the names used in the *ctsh* phylogenetic tree; Table S17. Table showing the correspondence between Ensembl IDs and the names used in the *ctss* phylogenetic tree; Table S18. Table showing the correspondence between Ensembl IDs and the names used in the *ctsz* phylogenetic tree; Table S19. Table showing the correspondence between Ensembl IDs and the names used in the *psmd11* phylogenetic tree; Table S20. Table showing the correspondence between Ensembl IDs and the names used in the *psmd4* phylogenetic tree; Table S21. Table showing the correspondence between Ensembl IDs and the names used in the *psme3* phylogenetic tree; Table S22. Table showing the correspondence between Ensembl IDs and the names used in the *psmb11* phylogenetic tree; Table S23. Table showing the correspondence between Ensembl IDs and the names used in the *psme4* phylogenetic tree; Table S24. Table showing the correspondence between Ensembl IDs and the names used in the *psma4* phylogenetic tree; Table S25. Table showing the correspondence between Ensembl IDs and the names used in the *psmb4* phylogenetic tree; Table S26. Table showing the correspondence between Ensembl IDs and the names used in the *psmd13* phylogenetic tree; Table S27. Table showing the correspondence between Ensembl IDs and the names used in the *psma6* phylogenetic tree; Table S28. Detailed sequencing quality-control and mapping statistics for both species for each pooled sample; Table S29. Coordinates of *capn1*, *capn2*, *capn8*, and *capn11* in *Dicentrarchus labrax* and Sparus aurata retrieved from Ensembl.
