# Supplementary material for: Distinct Evolutionary Trajectories of Major Intracellular Proteolytic Systems in Gilthead Seabream and European Seabass
Source: Biology (Basel). 2026 Oct 2;15(19):1755. doi: 10.3390/biology15191755 (PMC13664112; doi:10.3390/biology15191755)
Supplement: Supplementary file 1 [file biology-15-01755-s001.zip › biology-4522971-supplementary.pdf]

Supplementary Table S1. Human gene family members retrieved from the HGNC (HUGO Gene Nomenclature Committee) database with the corresponding Ensembl IDs

Gene name	Ensembl ID
PSMB8	ENSG00000204264
PSMB4	ENSG00000159377
PSMB9	ENSG00000240065
PSMB10	ENSG00000205220
PSMB5	ENSG00000100804
PSMA3	ENSG00000100567
PSMB1	ENSG00000008018
PSMA1	ENSG00000129084
POMP	ENSG00000132963
PSMA6	ENSG00000100902
PSMC5	ENSG00000087191
PSMB2	ENSG00000126067
PSMB6	ENSG00000142507
PSMD4	ENSG00000159352
PSMB7	ENSG00000136930
PSMA5	ENSG00000143106
PSMD2	ENSG00000175166
PSMG2	ENSG00000128789
PSMD1	ENSG00000173692
PSMA2	ENSG00000106588
PSMA4	ENSG00000041357
PSMC1	ENSG00000100764
PSMC2	ENSG00000161057
PSMC3	ENSG00000165916
PSMB3	ENSG00000277791
PSMD11	ENSG00000108671
PSMC4	ENSG00000013275
PSMD14	ENSG00000115233
PSMC6	ENSG00000100519
PSME3	ENSG00000131467
PSMD3	ENSG00000108344
PSMD13	ENSG00000185627
PSMD7	ENSG00000103035
PSMD12	ENSG00000197170
PSMD6	ENSG00000163636
PSMD8	ENSG00000099341
PSMD9	ENSG00000110801
PSME1	ENSG00000092010
PSMF1	ENSG00000125818
PSMD10	ENSG00000101843

PSME2	ENSG00000100911
PSMD5	ENSG00000095261
PSME4	ENSG00000068878
PSMA8	ENSG00000154611
ADRM1	ENSG00000130706
SEM1	ENSG00000127922
PSMB11	ENSG00000222028
ECPAS	ENSG00000136813
PSMG1	ENSG00000183527
PSMG3	ENSG00000157778
PSME3IP1	ENSG00000172775
CTSA	ENSG00000064601
CTSB	ENSG00000164733
CTSC	ENSG00000109861
CTSD	ENSG00000117984
CTSE	ENSG00000196188
CTSF	ENSG00000174080
CTSG	ENSG00000100448
CTSH	ENSG00000103811
CTSK	ENSG00000143387
CTSL	ENSG00000135047
CTSO	ENSG00000256043
CTSS	ENSG00000163131
CTSV	ENSG00000136943
CTSW	ENSG00000172543
CTSZ	ENSG00000101160
CAPN1	ENSG00000014216
CAPN2	ENSG00000162909
CAPN3	ENSG00000092529
CAPN5	ENSG00000149260
CAPN6	ENSG00000077274
CAPN7	ENSG00000131375
CAPN8	ENSG00000203697
CAPN9	ENSG00000135773
CAPN10	ENSG00000142330
CAPN11	ENSG00000137225
CAPN12	ENSG00000182472
CAPN13	ENSG00000162949
CAPN14	ENSG00000214711
CAPN15	ENSG00000103326
CAPNS1	ENSG00000126247
CAPNS2	ENSG00000256812

Supplementary Table S2. Species selected for the phylogenetic analysis and the accession numbers of each species genome. *: Stickleback Tetraodon and Lamprey no genome accession number was available; therefore, the corresponding Ensembl assembly ID is reported

Species	Representative of	Genome
Sparus aurata (Gilthead seabream)	Teleosts	GCA_900880675.1
Dicentrarchus labrax (European seabass)	Teleosts	GCA_905237075.1
Seriola dumerili (Greater amberjack)	Teleosts	GCA_002260705.1
Gasterosteus aculeatus (Stickleback)	Teleosts	GCA_016920845.1
Takifugu rubripes (Fugu)	Teleosts	GCA_901000725.2
Tetraodon nigroviridis (Tetraodon)	Teleosts	TETRAODON 8.0 *
Oreochromis niloticus (Nile tilapia)	Teleosts	GCA_001858045.3
Oryzias latipes (Japanese medaka)	Teleosts	GCA_002234675.1
Poecilia formosa (Amazon molly)	Teleosts	GCA_000485575.1
Xiphophorus maculatus (Platyfish)	Teleosts	GCA_002775205.2
Danio rerio (Zebrafish)	Teleosts	GCA_000002035.4
Salmo salar (Atlantic salmon)	Teleosts	GCA_905237065.2
Salmo trutta (Brown trout)	Teleosts	GCA_901001165.1
Oncorhynchus mykiss (Rainbow trout)	Teleosts	GCA_013265735.3
Oncorhynchus kisutch (Coho salmon)	Teleosts	GCA_002021735.2
Oncorhynchus tshawytscha (Chinook salmon)	Teleosts	GCA_018296145.1
Esox lucius (Northern pike)	Teleosts	GCA_011004845.1
Larimichthys crocea (Large yellow croaker)	Teleosts	GCA_000972845.2
Lepisosteus oculatus (Spotted gar)	Actinopterygii	GCA_000242695.1
Erpetoichthys calabaricus (Reedfish)	Actinopterygii	GCA_900747795.2
Latimeria chalumnae (Coelacanth)	Sarcopterygii	GCA_000225785.1
Homo sapiens (Human)	Sarcopterygii	GCA_000001405.29
Mus musculus (Mouse)	Sarcopterygii	GCA_000001635.9
Gallus gallus (Chicken)	Sarcopterygii	GCA_016699485.1
Meleagris gallopavo (Turkey)	Sarcopterygii	GCA_000146605.4
Taeniopygia guttata (Zebra finch)	Sarcopterygii	GCA_003957565.2
Anolis carolinensis (Green anole)	Sarcopterygii	GCA_000090745.2
Pelodiscus sinensis (Chinese softshell turtle)	Sarcopterygii	GCA_000230535.1
Xenopus tropicalis (Tropical clawed frog)	Sarcopterygii	GCA_000004195.4
Callorhinchus milii (Elephant shark)	Chondrichthyes	GCA_000165045.2
Petromyzon marinus (Lamprey)	Cyclostomata	Pmarinus_7.0 *
Ciona intestinalis (Vase tunicate)	Urochordata	GCA_000224145.1

Supplementary Table S3. Public RNAseq data from various tissues in both *S. aurata* and *D. labrax*. Missing tissue for each species is reported with "- "

Tissue	*S. aurata*	*D. labrax*	Library Type	Instrument
Brain	SRR6237496	ERR9715603	PE 150 bp	Illumina Hiseq

Intestine	SRR6237498	ERR9715623	PE 150 bp	Illumina Hiseq
Liver	SRR6237500	ERR9715614	PE 150 bp	Illumina Hiseq
White muscle	SRR6237499	ERR9715622	PE 150 bp	Illumina Hiseq
Embryonic stages	-	PRJEB52160	PE 150 bp	Illumina Hiseq
Post-hatching larvae stages	PRJNA1050571	PRJNA1050410	PE 150 bp	Illumina Hiseq

Supplementary Table S4. Correspondence between standardized gene names and Ensembl IDs in gilthead seabream and European seabass

Gene	*S.aurata*	*D. labrax*
capn1	ENSSAUG00010007897	ENSDLAG00005017924
capn2.1	ENSSAUG00010000032	ENSDLAG00005002296
capn2.2	ENSSAUG00010026026	ENSDLAG00005000590
capn2.3	ENSSAUG00010000030	-
capn2.4	ENSSAUG00010006640	-
capn2.5	ENSSAUG00010000034	-
capn3.1	ENSSAUG00010017861	ENSDLAG00005015494
capn3.2	ENSSAUG00010012311	ENSDLAG00005011625
capn5.1	ENSSAUG00010002636	ENSDLAG00005005420
capn5.2	ENSSAUG00010005676	ENSDLAG00005004342
capn6.1	ENSSAUG00010025836	ENSDLAG00005001788
capn6.2	ENSSAUG00010014146	ENSDLAG00005014943
capn7	ENSSAUG00010013017	ENSDLAG00005006030
capn8.1	ENSSAUG00010000033	ENSDLAG00005000702
capn8.2	ENSSAUG00010006205	-
capn8.3	ENSSAUG00010026019	-
capn9	ENSSAUG00010001056	ENSDLAG00005018075
capn10	ENSSAUG00010016851	ENSDLAG00005000250
capn11.1	ENSSAUG00010000077	ENSDLAG00005001439
capn11.2	ENSSAUG00010008141	ENSDLAG00005016201
capn11.3	ENSSAUG00010025995	-
capn12	ENSSAUG00010013339	ENSDLAG00005000961
capn14.1	ENSSAUG00010003429	ENSDLAG00005024962
capn14.2	ENSSAUG00010016749	ENSDLAG00005005672
capn14.3	ENSSAUG00010016757	-
capn15.1	ENSSAUG00010012619	ENSDLAG00005009199
capn15.2	ENSSAUG00010016176	ENSDLAG00005022265

capns1.1	ENSSAUG00010017388	ENSDLAG00005012396
capns1.2	ENSSAUG00010002445	ENSDLAG00005006529
ctsa	ENSSAUG00010005776	ENSDLAG00005013147
ctsb.1	ENSSAUG00010008071	ENSDLAG00005004816
ctsb.2	ENSSAUG00010003083	ENSDLAG00005013196
ctsc	ENSSAUG00010007964	ENSDLAG00005017730
ctsd.1	ENSSAUG00010015701	ENSDLAG00005022128
ctsd.2	ENSSAUG00010016838	ENSDLAG00005004808
ctsd.3	ENSSAUG00010016344	-
ctsf	ENSSAUG00010024233	ENSDLAG00005006074
ctsh.1	ENSSAUG00010015817	ENSDLAG00005023385
ctsh.2	ENSSAUG00010021061	-
ctsk	ENSSAUG00010011634	ENSDLAG00005014479
ctsl.1	ENSSAUG00010016582	ENSDLAG00005022121
ctsl.2	ENSSAUG00010010127	ENSDLAG00005007883
ctso	ENSSAUG00010002932	ENSDLAG00005022875
ctss.1	ENSSAUG00010011098	ENSDLAG00005005416
ctss.2	ENSSAUG00010011632	ENSDLAG00005014499
ctss.3	ENSSAUG00010011115	-
ctss.4	ENSSAUG00010011147	-
ctss.5	ENSSAUG00010017292	-
ctss.6	ENSSAUG00010011128	-
ctsz.1	ENSSAUG00010025140	ENSDLAG00005004507
ctsz.2	ENSSAUG00010014606	ENSDLAG00005011006
ctsz.3	ENSSAUG00010010858	ENSDLAG00005026027
adrm1	ENSSAUG00010027295	-
ecpas	ENSSAUG00010024842	-
pomp	ENSSAUG00010008220	ENSDLAG00005024500
psma1	ENSSAUG00010025303	ENSDLAG00005012225
psma2	ENSSAUG00010014669	ENSDLAG00005001650
psma3	ENSSAUG00010025465	ENSDLAG00005016689
psma4_1	ENSSAUG00010003232	ENSDLAG00005022298
psma4_2	ENSSAUG00010017501	ENSDLAG00005025222
psma5	ENSSAUG00010011510	ENSDLAG00005020772
psma6_1	ENSSAUG00010021478	ENSDLAG00005023268
psma6_2	ENSSAUG00010011210	ENSDLAG00005016118
psma8	ENSSAUG00010025737	ENSDLAG00005017693
psmb1	ENSSAUG00010004197	ENSDLAG00005007545
psmb10	ENSSAUG00010011974	ENSDLAG00005032590
psmb11_1	ENSSAUG00010018769	ENSDLAG00005018299
psmb11_2	ENSSAUG00010015241	ENSDLAG00005009568
psmb12	ENSSAUG00010020617	ENSDLAG00005031475
psmb2	ENSSAUG00010008400	ENSDLAG00005034806

psmb3	ENSSAUG00010001256	ENSDLAG00005011694
psmb4_1	ENSSAUG00010007698	ENSDLAG00005025170
psmb4_2	ENSSAUG00010007708	-
psmb5	ENSSAUG00010018758	ENSDLAG00005018277
psmb6	ENSSAUG00010002994	ENSDLAG00005020235
psmb7	ENSSAUG00010021443	ENSDLAG00005011418
psmb8a	ENSSAUG00010020623	-
psmb9a	ENSSAUG00010020612	ENSDLAG00005031842
psmc1b	ENSSAUG00010011143	ENSDLAG00005016370
psmc2	ENSSAUG00010024694	ENSDLAG00005025389
psmc3	ENSSAUG00010004152	ENSDLAG00005026507
psmc4	ENSSAUG00010023618	ENSDLAG00005001086
psmc5	ENSSAUG00010006373	ENSDLAG00005014093
psmc6	ENSSAUG00010002779	ENSDLAG00005020310
psmd1	ENSSAUG00010027595	ENSDLAG00005011359
psmd10	ENSSAUG00010026934	ENSDLAG00005010812
psmd11_1	ENSSAUG00010026051	ENSDLAG00005023007
psmd11_2	ENSSAUG00010021008	ENSDLAG00005032181
psmd12	ENSSAUG00010006905	ENSDLAG00005018025
psmd13_1	ENSSAUG00010016828	ENSDLAG00005022275
psmd13_2	ENSSAUG00010018216	-
psmd14	ENSSAUG00010021992	ENSDLAG00005016166
psmd2	ENSSAUG00010020121	ENSDLAG00005013752
psmd3	ENSSAUG00010015928	ENSDLAG00005010572
psmd4_1	ENSSAUG00010012828	ENSDLAG00005013691
psmd4_2	ENSSAUG00010018503	ENSDLAG00005013701
psmd5	ENSSAUG00010014769	ENSDLAG00005000700
psmd6	ENSSAUG00010006993	ENSDLAG00005023211
psmd7	ENSSAUG00010023937	ENSDLAG00005017766
psmd8	ENSSAUG00010021649	ENSDLAG00005031438
psmd9	ENSSAUG00010012274	ENSDLAG00005008258
psme1	ENSSAUG00010011610	ENSDLAG00005004361
psme2	ENSSAUG00010024593	ENSDLAG00005030547
psme3_1	ENSSAUG00010007133	ENSDLAG00005020891
psme3_2	ENSSAUG00010021784	-
psme3ip1	ENSSAUG00010011450	ENSDLAG00005014325
psme4_1	ENSSAUG00010023586	ENSDLAG00005014383
psme4_2	ENSSAUG00010026507	ENSDLAG00005020027
psmf1	ENSSAUG00010015466	ENSDLAG00005020243
psmg1	ENSSAUG00010002764	-
psmg2	ENSSAUG00010001825	ENSDLAG00005002548
psmg3	ENSSAUG00010002914	ENSDLAG00005015972
sem1	ENSSAUG00010013413	-

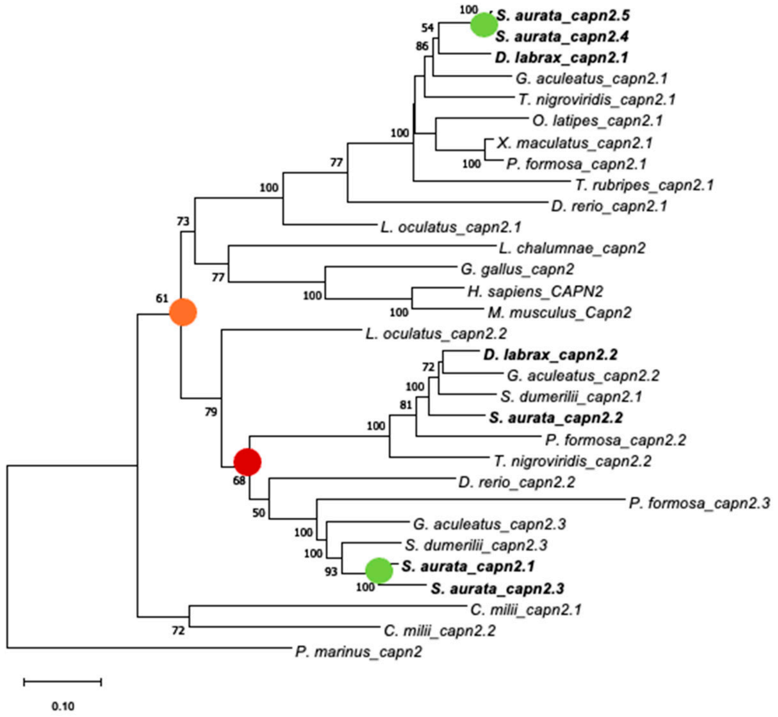

Supplementary Figure S1. Maximum-likelihood (ML) phylogenetic tree of the capn2 protein. The orange circle indicates the duplication node consistent with the first or second round of whole-genome duplication. The red circle indicates the duplication node consistent with the third round of whole-genome duplication, while the green circle indicates the duplication node specific to the gilthead seabream lineage. The substitution model used was T92+G+I. Numbers at the nodes represent bootstrap values.

Supplementary Table S5. Table showing the correspondence between Ensembl IDs and the names used in the capn2 phylogenetic tree.

Ensembl ID	*species*	*a/a*	*Tree ID*
ENSCMIG00000003092	*C. milii*	*capn2.1*	*C. milii_capn2.1*
ENSCMIG00000000577	*C. milii*	*capn2.2*	*C. milii_capn2.2*
ENSDLAG00005002296	*D. labrax*	*capn2.1*	***D. labrax_capn2.1***
ENSDLAG00005000590	*D. labrax*	*capn2.2*	***D. labrax_capn2.2***
ENSDARG00000055592	*D. rerio*	*capn2.1*	*D. rerio_capn2.1*
ENSDARG00000034211	*D. rerio*	*capn2.2*	*D. rerio_capn2.2*
ENSGACG00000019944	*G. aculeatus*	*capn2.1*	*G. aculeatus_capn2.1*
ENSGACG00000004767	*G. aculeatus*	*capn2.2*	*G. aculeatus_capn2.2*
ENSGACG00000019985	*G. aculeatus*	*capn2.3*	*G. aculeatus_capn2.3*
ENSGALG00000009360	*G. gallus*	capn2	*G. gallus_capn2*

ENSG00000162909	*H. sapiens*	CAPN2	*H. sapiens_CAPN2*
ENSLACG00000001902	*L. chalumnae*	capn2	*L. chalumnae_capn2*
ENSLOCG00000015646	*L. oculatus*	*capn2.1*	*L. oculatus_capn2.1*
ENSLOCG00000015661	*L. oculatus*	*capn2.2*	*L. oculatus_capn2.2*
ENSMUSG00000026509	*M. musculus*	*Capn2*	*M. musculus_Capn2*
ENSORLG00000020892	*O. latipes*	*capn2.1*	*O. latipes_capn2.1*
ENSPFOG00000018571	*P. formosa*	*capn2.1*	*P. formosa_capn2.1*
ENSPFOG00000016945	*P. formosa*	*capn2.2*	*P. formosa_capn2.2*
ENSPFOG00000018624	*P. formosa*	*capn2.3*	*P. formosa_capn2.3*
ENSPMAG00000007039	*P. marinus*	capn2	*P. marinus_capn2*
ENSSAUG00010000034	*S. aurata*	*capn2.5*	***S. aurata_capn2.5***
ENSSAUG00010006640	*S. aurata*	*capn2.4*	***S. aurata_capn2.4***
ENSSAUG00010026026	*S. aurata*	*capn2.2*	***S. aurata_capn2.2***
ENSSAUG00010000032	*S. aurata*	*capn2.1*	***S. aurata_capn2.1***
ENSSAUG00010000030	*S. aurata*	*capn2.3*	***S. aurata_capn2.3***
ENSSDUG00000001627	*S. dumerilii*	*capn2.1*	*S. dumerilii_capn2.1*
ENSSDUG00000021627	*S. dumerilii*	*capn2.3*	*S. dumerilii_capn2.3*
ENSTNIG00000005059	*T. nigroviridis*	*capn2.1*	*T. nigroviridis_capn2.1*
ENSTNIG00000016630	*T. nigroviridis*	*capn2.2*	*T. nigroviridis_capn2.2*
ENSTRUG00000012768	*T. rubripes*	*capn2.1*	*T. rubripes_capn2.1*
ENSXMAG00000008802	*X. maculatus*	*capn2.1*	*X. maculatus_capn2.1*

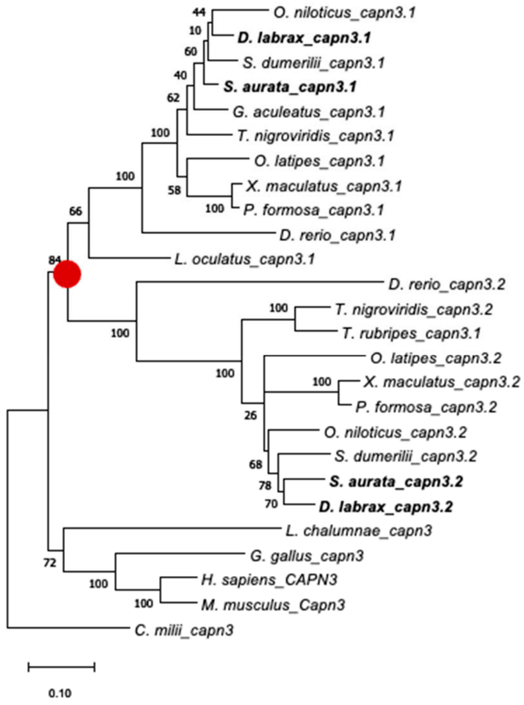

Supplementary Figure S2. Maximum-likelihood (ML) phylogenetic tree of the capn3 protein. The orange circle indicates the duplication node consistent with the third round of whole-

genome duplication. The substitution model used was GTR+G+I. Numbers at the nodes represent bootstrap values.

Supplementary Table S6. Table showing the correspondence between Ensembl IDs and the names used in the capn3 phylogenetic tree.

Ensembl ID	*species*	*a/a*	*Tree ID*
ENSCMIG00000016327	*C. milii*	*capn3*	*C. milii_capn3*
ENSDLAG00005015494	*D. labrax*	*capn3.1*	***D. labrax_capns3.1***
ENSDLAG00005011625	*D. labrax*	*capn3.2*	***D. labrax_capns3.2***
ENSDARG00000041864	*D. rerio*	*capn3.1*	*D. rerio_capns3.1*
ENSDARG00000043035	*D. rerio*	*capn3.2*	*D. rerio_capns3.2*
ENSGACG00000005212	*G. aculeatus*	*capn3.1*	*G. aculeatus_capns3.1*
ENSGALG00000009050	*G. gallus*	*capn3*	*G. gallus_capn3*
ENSG00000092529	*H. sapiens*	*CAPN3*	*H. sapiens_CAPN3*
ENSLACG00000003659	*L. chalumnae*	*capn3*	*L. chalumnae_capn3*
ENSLOCG00000013428	*L. oculatus*	*capns3.1*	*L. oculatus_capns3.1*
ENSMUSG00000079110	*M. musculus*	*Capn3*	*M. musculus_Capn3*
ENSORLG00000018135	*O. latipes*	*capns3.1*	*O. latipes_capns3.1*
ENSORLG00000012402	*O. latipes*	*capns3.2*	*O. latipes_capns3.2*
ENSONIG00000000403	*O. niloticus*	*capns3.1*	*O. niloticus_capns3.1*
ENSONIG00000006073	*O. niloticus*	*capns3.2*	*O. niloticus_capns3.2*
ENSPFOG00000006474	*P. formosa*	*capns3.1*	*P. formosa_capns3.1*
ENSPFOG00000019159	*P. formosa*	*capns3.2*	*P. formosa_capns3.2*
ENSSAUG00010017861	*S. aurata*	*capns3.1*	***S. aurata_capns3.1***
ENSSAUG00010012311	*S. aurata*	*capns3.2*	***S. aurata_capns3.2***
ENSSDUG00000001649	*S. dumerilii*	*capns3.1*	*S. dumerilii_capns3.1*
ENSSDUG00000002257	*S. dumerilii*	*capns3.2*	*S. dumerilii_capns3.2*
ENSTNIG00000010529	*T. nigroviridis*	*capns3.1*	*T. nigroviridis_capns3.1*
ENSTNIG00000003670	*T. nigroviridis*	*capns3.2*	*T. nigroviridis_capns3.2*
ENSTRUG00000018064	*T. rubripes*	*capns3.1*	*T. rubripes_capns3.1*
ENSXMAG00000009351	*X. maculatus*	*capns3.1*	*X. maculatus_capns3.1*
ENSXMAG00000017636	*X. maculatus*	*capns3.2*	*X. maculatus_capns3.2*

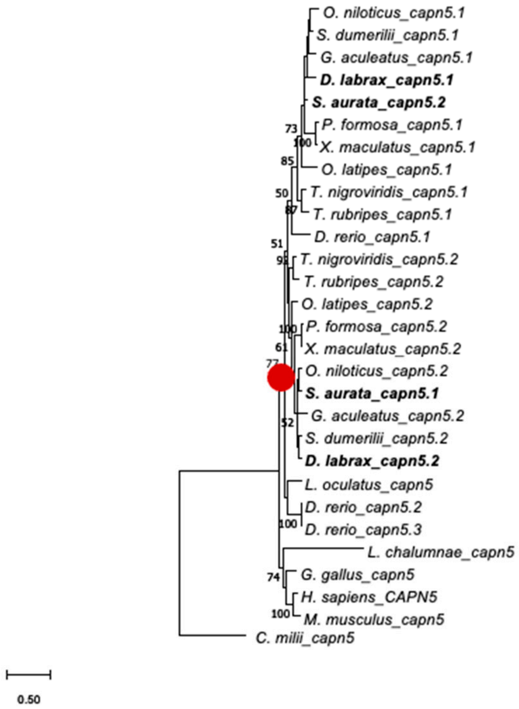

Supplementary Figure S3. Maximum-likelihood (ML) phylogenetic tree of the capn5 protein. The red circle indicates the duplication node consistent with the third round of whole-genome duplication. The substitution model used was HKY+G. Numbers at the nodes represent bootstrap values.

Supplementary Table S7. Table showing the correspondence between Ensembl IDs and the names used in the capn5 phylogenetic tree

Ensembl ID	*species*	*a/a*	*Tree ID*
ENSCMIG00000009156	*C. milii*	*capn5*	*C. milii_capn5*
ENSDLAG00005005420	*D. labrax*	*capn5.1*	***D. labrax_capn5.1***
ENSDLAG00005004342	*D. labrax*	*capn5.2*	***D. labrax_capn5.2***
ENSDARG00000103317	*D. rerio*	capn5.1	*D. rerio_capn5.1*
ENSDARG00000069748	*D. rerio*	capn5.2	*D. rerio_capn5.2*
ENSDARG00000116589	*D. rerio*	capn5.3	*D. rerio_capn5.3*
ENSGACG00000013281	*G. aculeatus*	*capn5.1*	*G. aculeatus_capn5.1*
ENSGACG00000020786	*G. aculeatus*	capn5.2	*G. aculeatus_capn5.2*
ENSGALG00000040478	*G. gallus*	*capn5*	*G. gallus_capn5*
ENSG00000149260	*H. sapiens*	*CAPN5*	*H. sapiens_CAPN5*
ENSLACG00000001126	*L. chalumnae*	*capn5*	*L. chalumnae_capn5*
ENSLOCG00000006677	*L. oculatus*	*capn5*	*L. oculatus_capn5*
ENSMUSG00000035547	*M. musculus*	*capn5*	*M. musculus_capn5*
ENSORLG00000002081	*O. latipes*	*capn5.1*	*O. latipes_capn5.1*
ENSORLG00000014975	*O. latipes*	capn5.2	*O. latipes_capn5.2*
ENSONIG00000007780	*O. niloticus*	*capn5.1*	*O. niloticus_capn5.1*
ENSONIG00000009550	*O. niloticus*	capn5.2	*O. niloticus_capn5.2*

ENSPFOG00000012108	*P. formosa*	*capn5.1*	*P. formosa_capn5.1*
ENSPFOG00000016938	*P. formosa*	capn5.2	*P. formosa_capn5.2*
ENSSAUG00010005676	*S. aurata*	*capn5.2*	***S. aurata_capn5.2***
ENSSAUG00010002636	*S. aurata*	capn5.1	***S. aurata_capn5.1***
ENSSDUG00000003779	*S. dumerilii*	*capn5.1*	*S. dumerilii_capn5.1*
ENSSDUG00000007506	*S. dumerilii*	capn5.2	*S. dumerilii_capn5.2*
ENSTNIG00000006028	*T. nigroviridis*	capn5.1	*T. nigroviridis_capn5.1*
ENSTNIG00000003712	*T. nigroviridis*	capn5.2	*T. nigroviridis_capn5.2*
ENSTRUG00000005683	*T. rubripes*	capn5.1	*T. rubripes_capn5.1*
ENSTRUG00000010580	*T. rubripes*	capn5.2	*T. rubripes_capn5.2*
ENSXMAG00000006995	*X. maculatus*	*capn5.1*	*X. maculatus_capn5.1*
ENSXMAG00000011169	*X. maculatus*	capn5.2	*X. maculatus_capn5.2*

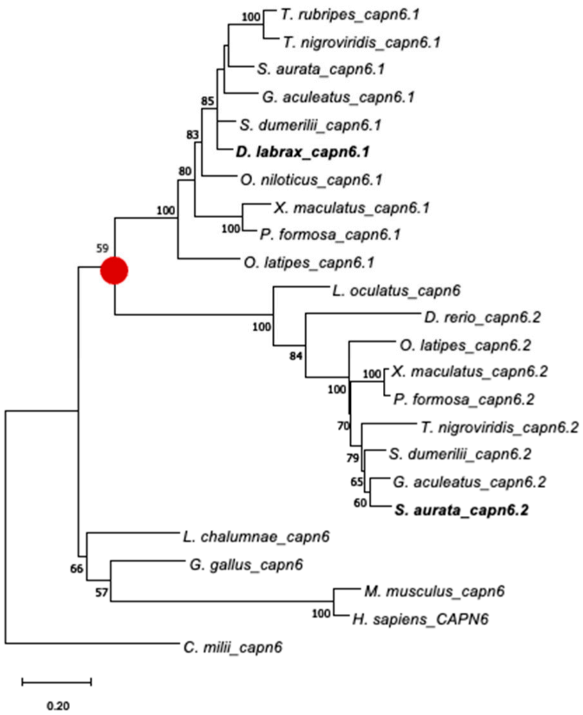

Supplementary Figure S4. Maximum-likelihood (ML) phylogenetic tree of the capn6 protein. The red circle indicates the duplication node consistent with the third round of whole-genome duplication. The substitution model used was T92+G+I. Numbers at the nodes represent bootstrap values.

Supplementary Table S8. Table showing the correspondence between Ensembl IDs and the names used in the capn6 phylogenetic tree

Ensembl ID	*species*	*a/a*	*Tree ID*

ENSCMIG00000016727	*C. milii*	capn6	*C. milii_capn6*
ENSDLAG00005001788	*D. labrax*	*capn6.1*	*D. labrax_capn6.1*
ENSDARG00000061850	*D. rerio*	capn6.2	*D. rerio_capn6.2*
ENSGACG00000020704	*G. aculeatus*	*capn6.1*	*G. aculeatus_capn6.1*
ENSGACG00000019314	*G. aculeatus*	capn6.2	*G. aculeatus_capn6.2*
ENSGALG00000008006	*G. gallus*	*capn6*	*G. gallus_capn6*
ENSG00000077274	*H. sapiens*	CAPN6	*H. sapiens_CAPN6*
ENSLACG00000006651	*L. chalumnae*	capn6	*L. chalumnae_capn6*
ENSLOCG00000014011	*L. oculatus*	capn6	*L. oculatus_capn6*
ENSMUSG00000067276	*m. musculus*	*capn6*	*m. musculus_capn6*
ENSORLG00000007043	*O. latipes*	capn6.1	*O. latipes_capn6.1*
ENSORLG00000006903	*O. latipes*	capn6.2	*O. latipes_capn6.2*
ENSONIG00000005243	*O. niloticus*	*capn6.1*	*O. niloticus_capn6.1*
ENSPFOG00000012541	*P. formosa*	capn6.1	*P. formosa_capn6.1*
ENSPFOG00000010952	*P. formosa*	capn6.2	*P. formosa_capn6.2*
ENSSAUG00010025836	*S. aurata*	*capn6.1*	***S. aurata_capn6.1***
ENSSAUG00010014146	*S. aurata*	capn6.2	***S. aurata_capn6.2***
ENSSDUG00000009382	*S. dumerilii*	*capn6.1*	*S. dumerilii_capn6.1*
ENSSDUG00000021396	*S. dumerilii*	capn6.2	*S. dumerilii_capn6.2*
ENSTNIG00000010048	*T. nigroviridis*	*capn6.1*	*T. nigroviridis_capn6.1*
ENSTNIG00000006853	*T. nigroviridis*	capn6.2	*T. nigroviridis_capn6.2*
ENSTRUG00000016780	*T. rubripes*	*capn6.1*	*T. rubripes_capn6.1*
ENSXMAG00000012735	*X. maculatus*	*capn6.1*	*X. maculatus_capn6.1*
ENSXMAG00000015348	*X. maculatus*	capn6.2	*X. maculatus_capn6.2*

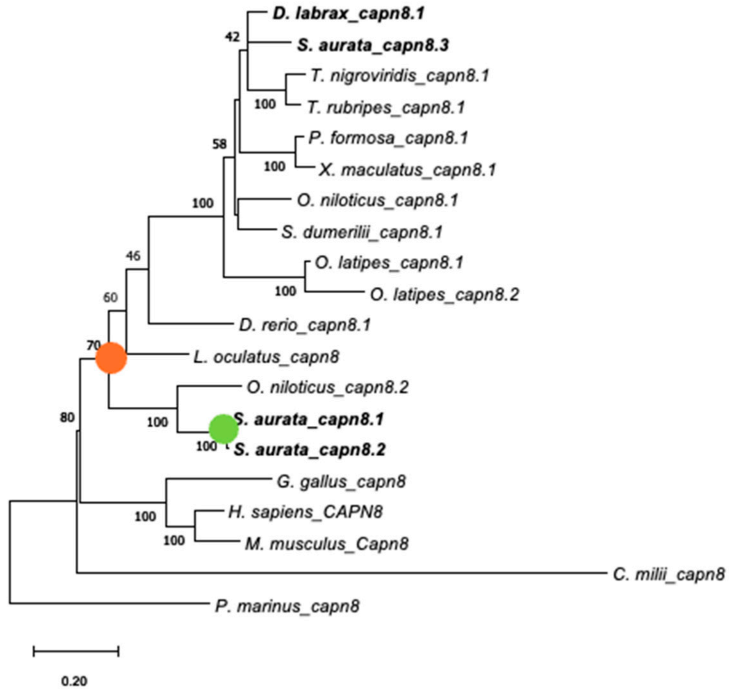

Supplementary Figure S5. Maximum-likelihood (ML) phylogenetic tree of the capn8 protein. The orange circle indicates the duplication node consistent with the first or second round of whole-genome duplication, while the green circle indicates the duplication node specific to the gilthead seabream lineage. The substitution model used was K2+G+I. Numbers at the nodes represent bootstrap values.

Supplementary Table S9. Table showing the correspondence between Ensembl IDs and the names used in the capn8 phylogenetic tree

Ensembl ID	*species*	*a/a*	*Tree ID*
ENSCMIG00000004869	*C. milii*	capn8	*C. milii_capn8*
ENSDLAG00005000702	*D. labrax*	*capns8.1*	***D. labrax_capns8.1***
ENSDARG00000055715	*D. rerio*	capns8.1	*D. rerio_capns8.1*
ENSGALG00000009382	*G. gallus*	capn8	*G. gallus_capn8*
ENSG00000203697	*H. sapiens*	*CAPN8*	*H. sapiens_CAPN8*
ENSLOCG00000015657	*L. oculatus*	capn8	*L. oculatus_capn8*
ENSMUSG00000038599	*M. musculus*	Capn8	*M. musculus_Capn8*
ENSORLG00000011856	*O. latipes*	capns8.1	*O. latipes_capns8.1*
ENSORLG00000023383	*O. latipes*	capns8.2	*O. latipes_capns8.2*
ENSONIG00000009988	*O. niloticus*	*capns8.1*	*O. niloticus_capns8.1*
ENSONIG00000007284	*O. niloticus*	capns8.2	*O. niloticus_capns8.2*
ENSPFOG00000022294	*P. formosa*	*capns8.1*	*P. formosa_capns8.1*
ENSPMAG00000007039	*P. marinus*	capn8	*P. marinus_capn8*
ENSSAUG00010026019	*S. aurata*	*capns8.3*	***S. aurata_capns8.3***
ENSSAUG00010000033	*S. aurata*	capns8.1	***S. aurata_capns8.1***
ENSSAUG00010006205	*S. aurata*	capns8.2	***S. aurata_capns8.2***

ENSSDUG00000001541	*S. dumerilii*	*capns8.1*	*S. dumerilii_capns8.1*
ENSTNIG00000016629	*T. nigroviridis*	*capns8.1*	*T. nigroviridis_capns8.1*
ENSTRUG00000007432	*T. rubripes*	*capns8.1*	*T. rubripes_capns8.1*
ENSXMAG00000006017	*X. maculatus*	*capns8.1*	*X. maculatus_capns8.1*

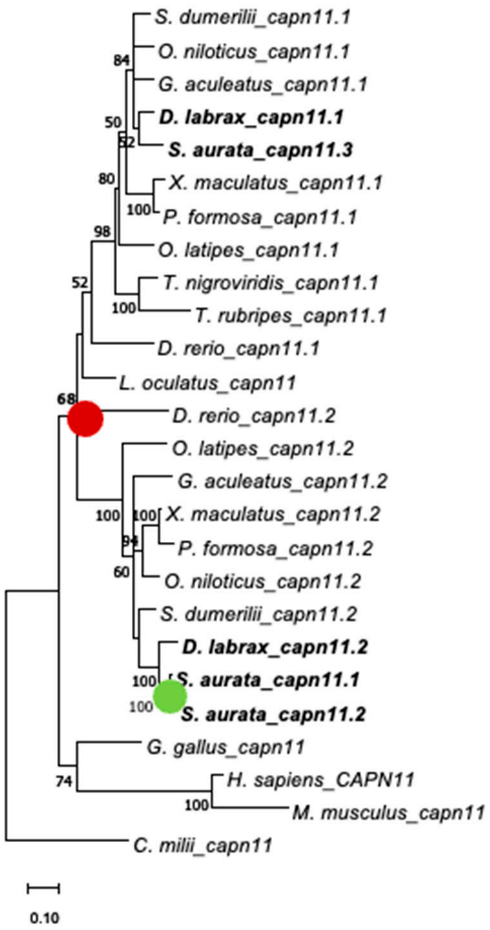

Supplementary Figure S6. Maximum-likelihood (ML) phylogenetic tree of the capn11 protein. The red circle indicates the duplication node consistent with the third round of whole-genome duplication. The green circle indicates the duplication node specific to the gilthead seabream lineage. The substitution model used was K2+G. Numbers at the nodes represent bootstrap values.

Supplementary Table S10. Table showing the correspondence between Ensembl IDs and the names used in the capn11 phylogenetic tree

Ensembl ID	*Species*	*a/a*	*Tree masked ID*
ENSCMIG00000000327	*C. milii*	*capn11*	*C. milii_capn11*
ENSDLAG00005001439	*D. labrax*	*capn11.1*	***D. labrax_capn11.1***
ENSDLAG00005016201	*D. labrax*	*capn11.2*	***D. labrax_capn11.2***
ENSDARG00000052702	*D. rerio*	*capn11.1*	*D. rerio_capn11.1*

ENSDARG00000052748	*D. rerio*	*capn11.2*	*D. rerio_capn11.2*
ENSGACG00000004833	*G. aculeatus*	*capn11.1*	*G. aculeatus_capn11.1*
ENSGACG00000019940	*G. aculeatus*	*capn11.2*	*G. aculeatus_capn11.2*
ENSGALG00000010186	*G. gallus*	*capn11*	*G. gallus_capn11*
ENSG00000137225	*H. sapiens*	*CAPN11*	*H. sapiens_CAPN11*
ENSLOCG00000015637	*L. oculatus*	*capn11*	*L. oculatus_capn11*
ENSMUSG00000058626	*M. musculus*	*capn11*	*M. musculus_capn11*
ENSORLG00000011738	*O. latipes*	*capn11.1*	*O. latipes_capn11.1*
ENSORLG00000022256	*O. latipes*	*capn11.2*	*O. latipes_capn11.2*
ENSONIG00000009965	*O. niloticus*	*capn11.1*	*O. niloticus_capn11.1*
ENSONIG00000007243	*O. niloticus*	*capn11.2*	*O. niloticus_capn11.2*
ENSPFOG00000016800	*P. formosa*	*capn11.1*	*P. formosa_capn11.1*
ENSPFOG00000018520	*P. formosa*	*capn11.2*	*P. formosa_capn11.2*
ENSSAUG00010025995	*S. aurata*	*capn11.3*	***S. aurata_capn11.3***
ENSSAUG00010000077	*S. aurata*	*capn11.1*	***S. aurata_capn11.1***
ENSSAUG00010008141	*S. aurata*	*capn11.2*	***S. aurata_capn11.2***
ENSSDUG00000001497	*S. dumerilii*	*capn11.1*	*S. dumerilii_capn11.1*
ENSSDUG00000021683	*S. dumerilii*	*capn11.2*	*S. dumerilii_capn11.2*
ENSTNIG00000016627	*T. nigroviridis*	*capn11.1*	*T. nigroviridis_capn11.1*
ENSTRUG00000008487	*T. rubripes*	*capn11.1*	*T. rubripes_capn11.1*
ENSXMAG00000005878	*X. maculatus*	*capn11.1*	*X. maculatus_capn11.1*
ENSXMAG00000008745	*X. maculatus*	*capn11.2*	*X. maculatus_capn11.2*

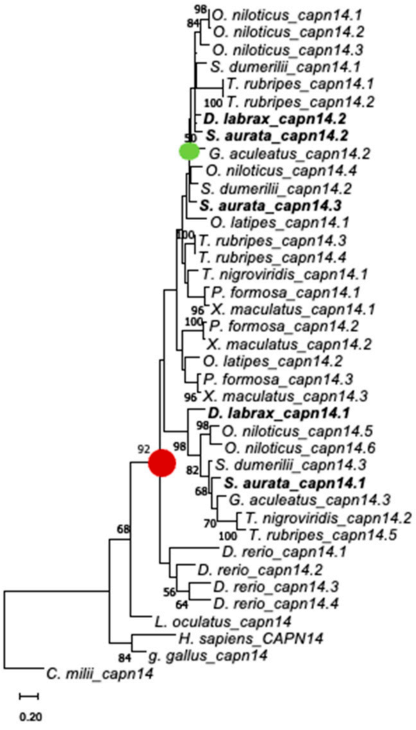

Supplementary Figure S7. Maximum-likelihood (ML) phylogenetic tree of the capn14 protein. The red circle indicates the duplication node consistent with the third round of whole-genome duplication. The green circle indicates the duplication node specific to the Percomorpha lineage. The substitution model used was K2+G. Numbers at the nodes represent bootstrap values.

Supplementary Table S11. Table showing the correspondence between Ensembl IDs and the names used in the capn14 phylogenetic tree

Ensembl ID	*species*	*a/a*	*Tree ID*
ENSCMIG00000018177	*C. milii*	*capn14*	*C. milii_capn14*
ENSDLAG00005005672	*D. labrax*	*capn14.2*	***D. labrax_capn14.2***
ENSDLAG00005024962	D. labrax	capn14.1	***D. labrax_capn14.1***
ENSDARG00000008553	*D. rerio*	capn14.1	*D. rerio_capn14.1*
ENSDARG00000077217	*D. rerio*	capn14.2	*D. rerio_capn14.2*
ENSDARG00000052917	*D. rerio*	capn14.3	*D. rerio_capn14.3*
ENSDARG00000091723	*D. rerio*	capn14.4	*D. rerio_capn14.4*
ENSGACG00000005683	*G. aculeatus*	capn14.2	*G. aculeatus_capn14.2*
ENSGACG00000020515	*G. aculeatus*	capn14.3	*G. aculeatus_capn14.3*
ENSGALG00000009069	*g. gallus*	capn14	*g. gallus_capn14*
ENSG00000214711	*H. sapiens*	CAPN14	*H. sapiens_CAPN14*
ENSLOCG00000016403	*L. oculatus*	capn14	*L. oculatus_capn14*
ENSORLG00000019014	*O. latipes*	capn14.1	*O. latipes_capn14.1*
ENSORLG00000019002	*O. latipes*	capn14.2	*O. latipes_capn14.2*
ENSONIG00000034252	*O. niloticus*	*capn14.1*	*O. niloticus_capn14.1*
ENSONIG00000034793	*O. niloticus*	*capn14.2*	*O. niloticus_capn14.2*
ENSONIG00000033326	*O. niloticus*	*capn14.3*	*O. niloticus_capn14.3*
ENSONIG00000027569	*O. niloticus*	capn14.4	*O. niloticus_capn14.4*
ENSONIG00000005520	*O. niloticus*	capn14.5	*O. niloticus_capn14.5*
ENSONIG00000005524	*O. niloticus*	capn14.6	*O. niloticus_capn14.6*
ENSPFOG00000009124	*P. formosa*	capn14.1	*P. formosa_capn14.1*
ENSPFOG00000010475	*P. formosa*	*capn14.2*	*P. formosa_capn14.2*
ENSPFOG00000010042	*P. formosa*	capn14.3	*P. formosa_capn14.3*
ENSSAUG00010016749	*S. aurata*	*capn14.2*	***S. aurata_capn14.2***
ENSSAUG00010016757	*S. aurata*	capn14.3	***S. aurata_capn14.3***
ENSSAUG00010003429	*S. aurata*	capn14.1	***S. aurata_capn14.1***
ENSSDUG00000013563	*S. dumerilii*	*capn14.1*	*S. dumerilii_capn14.1*
ENSSDUG00000013642	*S. dumerilii*	capn14.2	*S. dumerilii_capn14.2*
ENSSDUG00000016700	*S. dumerilii*	capn14.3	*S. dumerilii_capn14.3*
ENSTNIG00000001585	*T. nigroviridis*	capn14.1	*T. nigroviridis_capn14.1*
ENSTNIG00000016160	*T. nigroviridis*	capn14.2	*T. nigroviridis_capn14.2*
ENSTRUG00000001713	*T. rubripes*	*capn14.1*	*T. rubripes_capn14.1*
ENSTRUG00000030387	*T. rubripes*	*capn14.2*	*T. rubripes_capn14.2*
ENSTRUG00000002373	*T. rubripes*	capn14.3	*T. rubripes_capn14.3*
ENSTRUG00000027205	*T. rubripes*	capn14.4	*T. rubripes_capn14.4*
ENSTRUG00000000168	*T. rubripes*	capn14.5	*T. rubripes_capn14.5*

ENSXMAG00000000065	*X. maculatus*	*capn14.1*	*X. maculatus_capn14.1*
ENSXMAG00000000188	*X. maculatus*	capn14.2	*X. maculatus_capn14.2*
ENSXMAG00000000132	*X. maculatus*	*capn14.3*	*X. maculatus_capn14.3*

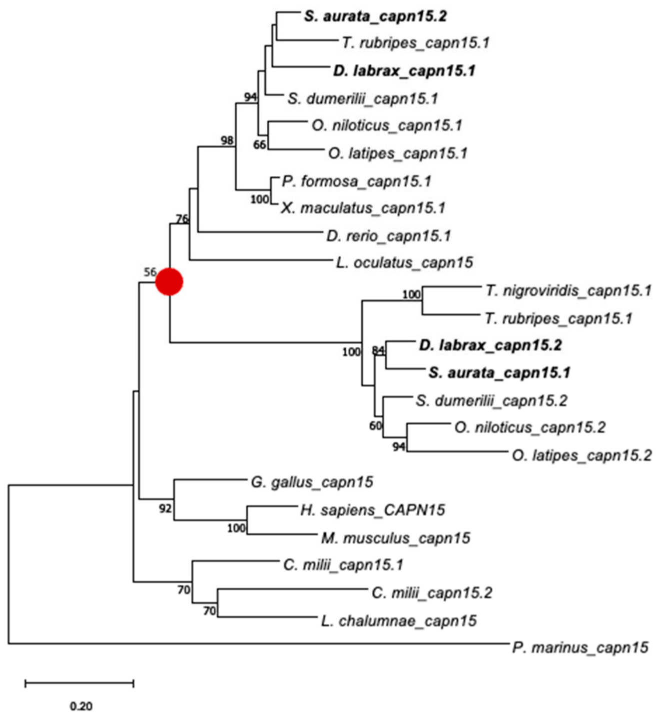

Supplementary Figure S8. Maximum-likelihood (ML) phylogenetic tree of the capn15 protein. The red circle indicates the duplication node consistent with the third round of whole-genome duplication. The substitution model used was T92+G. Numbers at the nodes represent bootstrap values.

Supplementary Table S12. Table showing the correspondence between Ensembl IDs and the names used in the capn15 phylogenetic tree

Ensembl ID	*species*	*a/a*	*Tree ID*
ENSCMIG00000008010	*C. milii*	*capn15.1*	*C. milii_capn15.1*
ENSCMIG00000008014	*C. milii*	*capn15.2*	*C. milii_capn15.2*
ENSDLAG00005009199	*D. labrax*	*capn15.1*	***D. labrax_capn15.1***
ENSDLAG00005022265	*D. labrax*	capn15.2	***D. labrax_capn15.2***
ENSDARG00000060600	*D. rerio*	capn15.1	*D. rerio_capn15.1*
ENSGALG00000002018	*G. gallus*	capn15	*G. gallus_capn15*
ENSG00000103326	*H. sapiens*	CAPN15	*H. sapiens_CAPN15*
ENSLACG00000016666	*L. chalumnae*	capn15	*L. chalumnae_capn15*
ENSLOCG00000009003	*L. oculatus*	capn15	*L. oculatus_capn15*

ENSMUSG00000037326	*M. musculus*	capn15	*M. musculus_capn15*
ENSORLG00000007128	*O. latipes*	*capn15.1*	*O. latipes_capn15.1*
ENSORLG00000000290	*O. latipes*	capn15.2	*O. latipes_capn15.2*
ENSONIG00000020030	*O. niloticus*	*capn15.1*	*O. niloticus_capn15.1*
ENSONIG00000009415	*O. niloticus*	capn15.2	*O. niloticus_capn15.2*
ENSPFOG00000017205	*P. formosa*	*capn15.1*	*P. formosa_capn15.1*
ENSPMAG00000004340	*P. marinus*	capn15	*P. marinus_capn15*
ENSSAUG00010016176	*S. aurata*	*capn15.2*	***S. aurata_capn15.2***
ENSSAUG00010012619	*S. aurata*	capn15.1	***S. aurata_capn15.1***
ENSSDUG00000000678	*S. dumerilii*	*capn15.1*	*S. dumerilii_capn15.1*
ENSSDUG00000000928	*S. dumerilii*	capn15.2	*S. dumerilii_capn15.2*
ENSTNIG00000004278	*T. nigroviridis*	capn15.1	*T. nigroviridis_capn15.1*
ENSTRUG00000014283	*T. rubripes*	*capn15.1*	*T. rubripes_capn15.1*
ENSTRUG00000031299	*T. rubripes*	capn15.1	*T. rubripes_capn15.1*
ENSXMAG00000019044	*X. maculatus*	*capn15.1*	*X. maculatus_capn15.1*

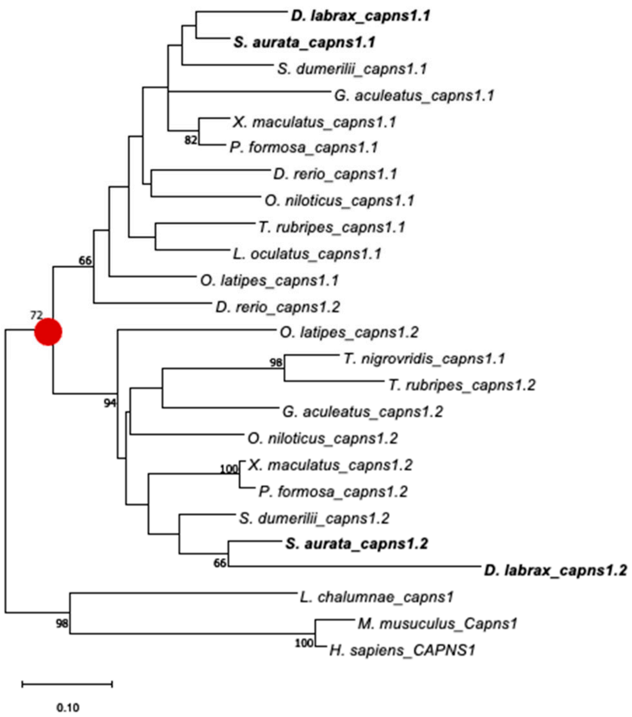

Supplementary Figure S9. Maximum-likelihood (ML) phylogenetic tree of the capns1 protein. The red circle indicates the duplication node consistent with the third round of whole-genome duplication. The substitution model used was JTT+G. Numbers at the nodes represent bootstrap values.

Supplementary Table S13. Table showing the correspondence between Ensembl IDs and the names used in the capns1 phylogenetic tree

Ensembl ID	*species*	*a/a*	*Tree ID*
ENSDLAG00005006529	*D. labrax*	*capns1.1*	***D. labrax_capns1.1***
ENSDLAG00005012396	*D. labrax*	*capns1.2*	***D. labrax_capns1.2***
ENSDARG00000035329	*D. rerio*	*capns1.1*	*D. rerio_capns1.1*
ENSDARG00000013804	*D. rerio*	capns1.2	*D. rerio_capns1.2*
ENSGACG00000020174	*G. aculeatus*	*capns1.1*	*G. aculeatus_capns1.1*
ENSGACG00000013438	*G. aculeatus*	capns1.2	*G. aculeatus_capns1.2*
ENSG00000126247	*H. sapiens*	*CAPNS1*	*H. sapiens_CAPNS1*
ENSLACG00000004905	*L. chalumnae*	capns1	*L. chalumnae_capns1*
ENSLOCG00000014756	*L. oculatus*	capns1.1	*L. oculatus_capns1.1*
ENSMUSG00000001794	*M. musuculus*	Capns1	*M. musuculus_Capns1*
ENSORLG00000000369	*O. latipes*	capns1.1	*O. latipes_capns1.1*
ENSORLG00000000490	*O. latipes*	capns1.2	*O. latipes_capns1.2*
ENSONIG00000027340	*O. niloticus*	*capns1.1*	*O. niloticus_capns1.1*
ENSONIG00000001239	*O. niloticus*	capns1.2	*O. niloticus_capns1.2*
ENSPFOG00000018021	*P. formosa*	*capns1.1*	*P. formosa_capns1.1*
ENSPFOG00000006637	*P. formosa*	capns1.2	*P. formosa_capns1.2*
ENSSAUG00010017388	*S. aurata*	*capns1.1*	***S. aurata_capns1.1***
ENSSAUG00010002445	*S. aurata*	*capns1.2*	***S. aurata_capns1.2***
ENSSDUG00000020449	*S. dumerilii*	*capns1.1*	*S. dumerilii_capns1.1*
ENSSDUG00000004797	*S. dumerilii*	capns1.2	*S. dumerilii_capns1.2*
ENSTNIG00000011172	*T. nigrovridis*	capns1.1	*T. nigrovridis_capns1.1*
ENSTRUG00000006704	*T. rubripes*	capns1.1	*T. rubripes_capns1.1*
ENSTRUG00000026782	*T. rubripes*	capns1.2	*T. rubripes_capns1.2*
ENSXMAG00000017885	*X. maculatus*	*capns1.1*	*X. maculatus_capns1.1*
ENSXMAG00000015233	*X. maculatus*	capns1.2	*X. maculatus_capns1.2*

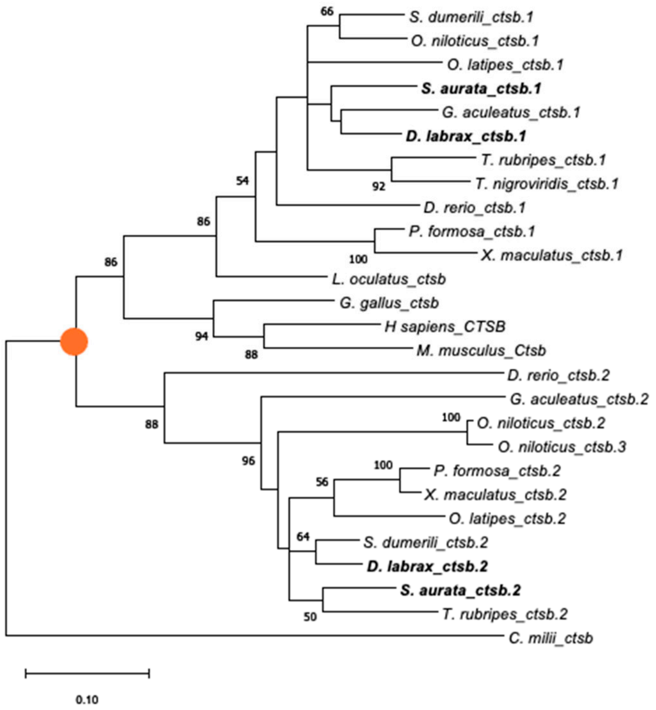

Supplementary Figure S10. Maximum-likelihood (ML) phylogenetic tree of the ctsb protein. The orange circle indicates the duplication node consistent with the second round of whole-genome duplication. The substitution model used was JTT+G+I. Numbers at the nodes represent bootstrap values.

Supplementary Table S14. Table showing the correspondence between Ensembl IDs and the names used in the ctsb phylogenetic tree

Ensembl ID	*species*	*a/a*	*Tree ID*
ENSCMIG00000000073	*C. milii*	ctsb	*C. milii_ctsb*
ENSDLAG00005004816	*D. labrax*	*ctsb.1*	***D. labrax_ctsb.1***
ENSDLAG00005013196	*D. labrax*	ctsb.2	***D. labrax_ctsb.2***
ENSDARG00000055120	*D. rerio*	*ctsb.1*	*D. rerio_ctsb.1*
ENSDARG00000101051	*D. rerio*	ctsb.2	*D. rerio_ctsb.2*
ENSGACG00000016202	*G. aculeatus*	*ctsb.1*	*G. aculeatus_ctsb.1*
ENSGACG00000010234	*G. aculeatus*	ctsb.2	*G. aculeatus_ctsb.2*
ENSGALG00000030016	*G. gallus*	ctsb	*G. gallus_ctsb*
ENSG00000164733	*H sapiens*	CTSB	*H sapiens_CTSB*
ENSLOCG00000000768	*L. oculatus*	ctsb	*L. oculatus_ctsb*
ENSMUSG00000021939	*M. musculus*	Ctsb	*M. musculus_Ctsb*
ENSORLG00000011080	*O. latipes*	*ctsb.1*	*O. latipes_ctsb.1*
ENSORLG00000017347	*O. latipes*	ctsb.2	*O. latipes_ctsb.2*
ENSONIG00000017101	*O. niloticus*	*ctsb.1*	*O. niloticus_ctsb.1*
ENSONIG00000038588	*O. niloticus*	ctsb.2	*O. niloticus_ctsb.2*
ENSONIG00000040884	*O. niloticus*	ctsb.3	*O. niloticus_ctsb.3*
ENSPFOG00000013851	*P. formosa*	*ctsb.1*	*P. formosa_ctsb.1*

ENSPFOG00000005337	*P. formosa*	ctsb.2	*P. formosa_ctsb.2*
ENSSAUG00010008071	*S. aurata*	*ctsb.1*	***S. aurata_ctsb.1***
ENSSAUG00010003083	*S. aurata*	ctsb.2	***S. aurata_ctsb.2***
ENSSDUG00000001149	*S. dumerili*	*ctsb.1*	*S. dumerili_ctsb.1*
ENSSDUG00000015853	*S. dumerili*	ctsb.2	*S. dumerili_ctsb.2*
ENSTNIG00000017695	*T. nigroviridis*	*ctsb.1*	*T. nigroviridis_ctsb.1*
ENSTRUG00000009391	*T. rubripes*	*ctsb.1*	*T. rubripes_ctsb.1*
ENSTRUG00000012315	*T. rubripes*	ctsb.2	*T. rubripes_ctsb.2*
ENSXMAG00000015152	*X. maculatus*	*ctsb.1*	*X. maculatus_ctsb.1*
ENSXMAG00000003780	*X. maculatus*	ctsb.2	*X. maculatus_ctsb.2*

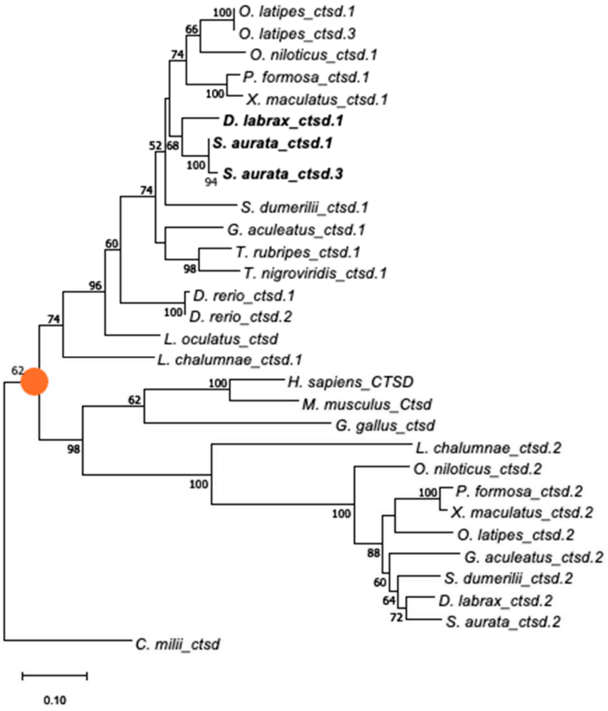

Supplementary Figure S11. Maximum-likelihood (ML) phylogenetic tree of the ctsd protein. The orange circle indicates the duplication node consistent with the second round of whole-genome duplication. The substitution model used was JTT+G+I. Numbers at the nodes represent bootstrap values.

Supplementary Table S15. Table showing the correspondence between Ensembl IDs and the names used in the ctsd phylogenetic tree

Ensembl ID	*species*	*a/a*	*Tree ID*
ENSCMIG00000011996	*C. milii*	ctsd	*C. milii_ctsd*
ENSDLAG00005022128	*D. labrax*	*ctsd.1*	***D. labrax_ctsd.1***
ENSDLAG00005004808	*D. labrax*	*ctsd.2*	***D. labrax_ctsd.2***
ENSDARG00000057698	*D. rerio*	*ctsd.1*	*D. rerio_ctsd.1*

ENSDARG00000113876	*D. rerio*	*ctsd.2*	*D. rerio_ctsd.2*
ENSGACG00000017247	*G. aculeatus*	*ctsd.1*	*G. aculeatus_ctsd.1*
ENSGACG00000008090	*G. aculeatus*	*ctsd.2*	*G. aculeatus_ctsd.2*
ENSGALG00000006613	*G. gallus*	ctsd	*G. gallus_ctsd*
ENSG00000117984	*H. sapiens*	CTSD	*H. sapiens_CTSD*
ENSLACG00000002616	*L. chalumnae*	ctsd.1	*L. chalumnae_ctsd.1*
ENSLACG00000014748	*L. chalumnae*	ctsd.2	*L. chalumnae_ctsd.2*
ENSLOCG00000002297	*L. oculatus*	ctsd	*L. oculatus_ctsd*
ENSMUSG00000007891	*M. musculus*	Ctsd	*M. musculus_Ctsd*
ENSORLG00000000092	*O. latipes*	*ctsd.1*	*O. latipes_ctsd.1*
ENSORLG00000027285	*O. latipes*	*ctsd.3*	*O. latipes_ctsd.3*
ENSORLG00000013465	*O. latipes*	*ctsd.2*	*O. latipes_ctsd.2*
ENSONIG00000015575	*O. niloticus*	*ctsd.1*	*O. niloticus_ctsd.1*
ENSONIG00000008542	*O. niloticus*	*ctsd.2*	*O. niloticus_ctsd.2*
ENSPFOG00000001622	*P. formosa*	*ctsd.1*	*P. formosa_ctsd.1*
ENSPFOG00000005248	*P. formosa*	*ctsd.2*	*P. formosa_ctsd.2*
ENSSAUG00010015701	*S. aurata*	*ctsd.1*	***S. aurata_ctsd.1***
ENSSAUG00010016838	*S. aurata*	*ctsd.3*	***S. aurata_ctsd.3***
ENSSAUG00010016344	*S. aurata*	*ctsd.2*	***S. aurata_ctsd.2***
ENSSDUG00000019768	*S. dumerilii*	*ctsd.1*	*S. dumerilii_ctsd.1*
ENSSDUG00000018135	*S. dumerilii*	*ctsd.2*	*S. dumerilii_ctsd.2*
ENSTNIG00000003945	*T. nigroviridis*	*ctsd.1*	*T. nigroviridis_ctsd.1*
ENSTRUG00000012591	*T. rubripes*	*ctsd.1*	*T. rubripes_ctsd.1*
ENSXMAG00000018351	*X. maculatus*	*ctsd.1*	*X. maculatus_ctsd.1*
ENSXMAG00000029235	*X. maculatus*	*ctsd.2*	*X. maculatus_ctsd.2*

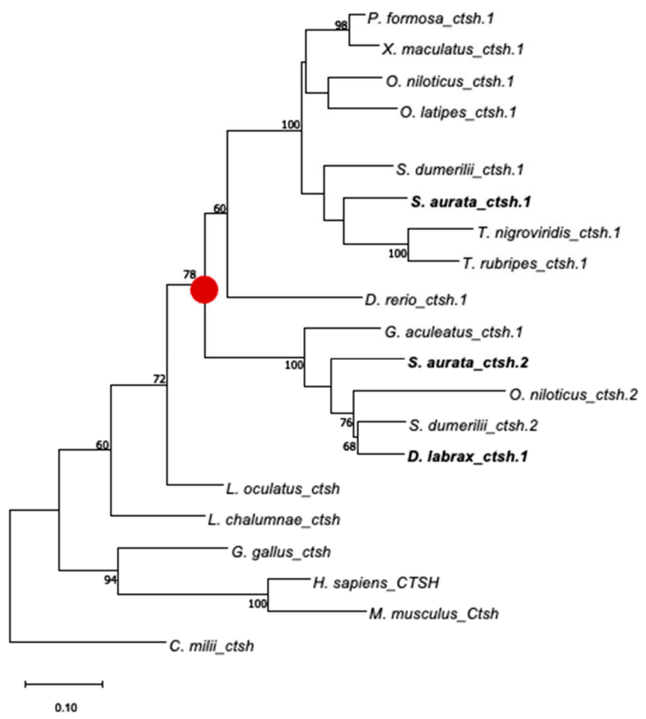

Supplementary Figure S12. Maximum-likelihood (ML) phylogenetic tree of the ctsh protein. The red circle indicates the duplication node consistent with the TS-WGD. The substitution model used was JTT+G+I. Numbers at the nodes represent bootstrap values.

Supplementary Table S16. Table showing the correspondence between Ensembl IDs and the names used in the ctsh phylogenetic tree

Ensembl ID	*species*	*a/a*	*Tree ID*
ENSCMIG00000018300	*C. milii*	ctsh	*C. milii_ctsh*
ENSDLAG00005023385	*D. labrax*	*ctsh.1*	***D. labrax_ctsh.1***
ENSDARG00000041108	*D. rerio*	*ctsh.1*	*D. rerio_ctsh.1*
ENSGACG00000016451	*G. aculeatus*	*ctsh.1*	*G. aculeatus_ctsh.1*
ENSGALG00000033557	*G. gallus*	ctsh	*G. gallus_ctsh*
ENSG00000103811	*H. sapiens*	CTSH	*H. sapiens_CTSH*
ENSLACG00000005657	*L. chalumnae*	ctsh	*L. chalumnae_ctsh*
ENSLOCG00000014308	*L. oculatus*	ctsh	*L. oculatus_ctsh*
ENSMUSG00000032359	*M. musculus*	Ctsh	*M. musculus_Ctsh*
ENSORLG00000023479	*O. latipes*	*ctsh.1*	*O. latipes_ctsh.1*
ENSORLG00000017347	*O. niloticus*	*ctsh.1*	*O. niloticus_ctsh.1*
ENSONIG00000015188	*O. niloticus*	*ctsh.2*	*O. niloticus_ctsh.2*
ENSPFOG00000024227	*P. formosa*	*ctsh.1*	*P. formosa_ctsh.1*
ENSSAUG00010021061	*S. aurata*	*ctsh.1*	***S. aurata_ctsh.1***
ENSSAUG00010015817	*S. aurata*	*ctsh.2*	***S. aurata_ctsh.2***
ENSSDUG00000010987	*S. dumerilii*	*ctsh.1*	*S. dumerilii_ctsh.1*
ENSSDUG00000003990	*S. dumerilii*	*ctsh.2*	*S. dumerilii_ctsh.2*
ENSTNIG00000019245	*T. nigroviridis*	*ctsh.1*	*T. nigroviridis_ctsh.1*

ENSTRUG00000005261	*T. rubripes*	*ctsh.1*	*T. rubripes_ctsh.1*
ENSXMAG00000002937	*X. maculatus*	*ctsh.1*	*X. maculatus_ctsh.1*

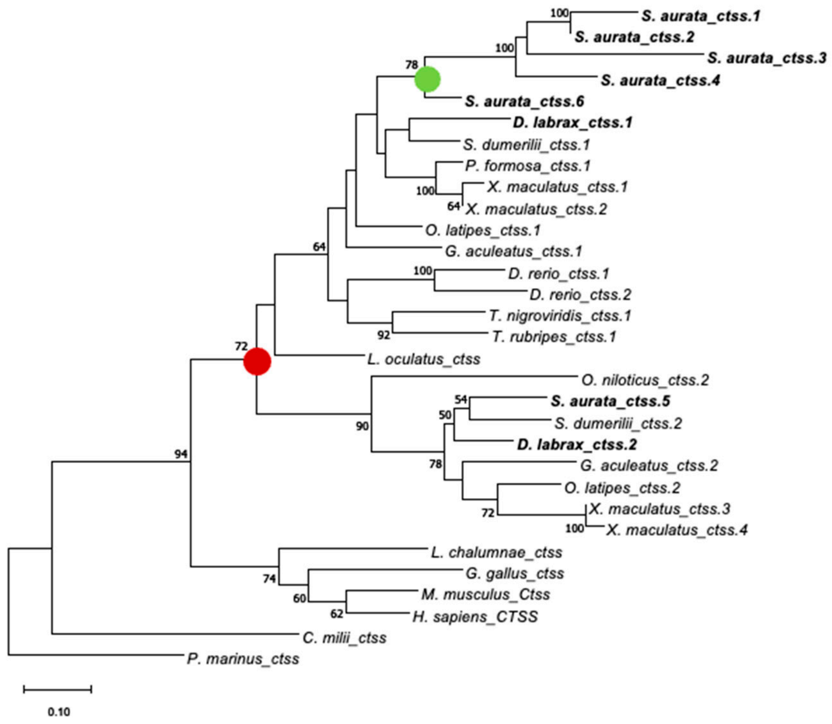

Supplementary Figure S13. Maximum-likelihood (ML) phylogenetic tree of the ctss protein. The red circle indicates the duplication node consistent with the TS-WGD while the green circle indicates a single lineage duplication node. The substitution model used was JTT+G. Numbers at the nodes represent bootstrap values.

Supplementary Table S17. Table showing the correspondence between Ensembl IDs and the names used in the ctss phylogenetic tree

Ensembl ID	*species*	*a/a*	*Tree ID*
ENSCMIG00000018924	*C. milii*	ctss	*C. milii_ctss*
ENSDLAG00005014499	*D. labrax*	*ctss.1*	***D. labrax_ctss.1***
ENSDLAG00005005416	*D. labrax*	ctss.2	***D. labrax_ctss.2***
ENSDARG00000013771	*D. rerio*	*ctss.1*	*D. rerio_ctss.1*
ENSDARG00000074656	*D. rerio*	*ctss.2*	*D. rerio_ctss.2*
ENSGACG00000013470	*G. aculeatus*	*ctss.1*	*G. aculeatus_ctss.1*
ENSGACG00000003870	*G. aculeatus*	ctss.2	*G. aculeatus_ctss.2*
ENSGALG00000000775	*G. gallus*	ctss	*G. gallus_ctss*
ENSG00000163131	*H. sapiens*	CTSS	*H. sapiens_CTSS*
ENSLACG00000000085	*L. chalumnae*	ctss	*L. chalumnae_ctss*
ENSLOCG00000007929	*L. oculatus*	ctss	*L. oculatus_ctss*
ENSMUSG00000038642	*M. musculus*	Ctss	*M. musculus_Ctss*

ENSORLG00000015733	*O. latipes*	*ctss.1*	*O. latipes_ctss.1*
ENSORLG00000003145	*O. latipes*	ctss.2	*O. latipes_ctss.2*
ENSONIG00000002754	*O. niloticus*	ctss.2	*O. niloticus_ctss.2*
ENSONIG00000002559	*P. formosa*	*ctss.1*	*P. formosa_ctss.1*
ENSPFOG00000007895	*P. marinus*	ctss	*P. marinus_ctss*
ENSPMAG00000005817	*S. aurata*	*ctss.1*	***S. aurata_ctss.1***
ENSSAUG00010011128	*S. aurata*	ctss.2	***S. aurata_ctss.2***
ENSSAUG00010011632	*S. aurata*	ctss.3	***S. aurata_ctss.3***
ENSSAUG00010011147	*S. aurata*	ctss.4	***S. aurata_ctss.4***
ENSSAUG00010011115	*S. aurata*	ctss.6	***S. aurata_ctss.6***
ENSSAUG00010011098	*S. aurata*	ctss.5	***S. aurata_ctss.5***
ENSSAUG00010017292	*S. dumerilii*	*ctss.1*	*S. dumerilii_ctss.1*
ENSSDUG00000002074	*S. dumerilii*	ctss.2	*S. dumerilii_ctss.2*
ENSSDUG00000012123	*T. nigroviridis*	*ctss.1*	*T. nigroviridis_ctss.1*
ENSTNIG00000006174	*T. rubripes*	*ctss.1*	*T. rubripes_ctss.1*
ENSXMAG00000003793	*X. maculatus*	*ctss.1*	*X. maculatus_ctss.1*
ENSXMAG00000003801	*X. maculatus*	*ctss.2*	*X. maculatus_ctss.2*
ENSXMAG00000008983	*X. maculatus*	ctss.3	*X. maculatus_ctss.3*
ENSXMAG00000008976	*X. maculatus*	ctss.4	*X. maculatus_ctss.4*

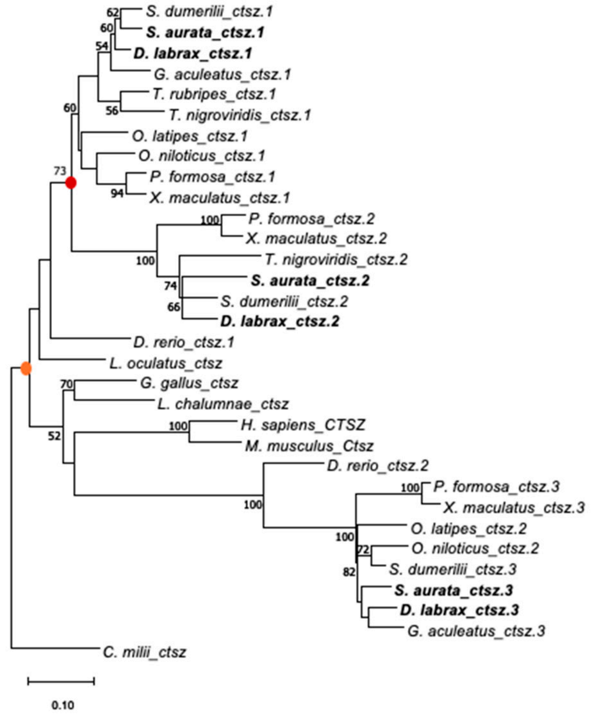

Supplementary Figure S14. Maximum-likelihood (ML) phylogenetic tree of the ctsz protein. The red circle indicates the duplication node consistent with the TS-WGD, while the orange circle indicates the duplication node consistent with the first or second round of whole-genome

duplication. The substitution model used was JTT+G+I. Numbers at the nodes represent bootstrap values.

Supplementary Table S18. Table showing the correspondence between Ensembl IDs and the names used in the ctsz phylogenetic tree

Ensembl ID	*species*	*a/a*	*Tree ID*
ENSCMIG00000015140	*C. milii*	ctsz	C. milii_ctsz
ENSDLAG00005004507	*D. labrax*	ctsz.1	**D. labrax_ctsz.1**
ENSDLAG00005011006	*D. labrax*	ctsz.2	**D. labrax_ctsz.2**
ENSDLAG00005026027	*D. labrax*	ctsz.3	**D. labrax_ctsz.3**
ENSDARG00000043081	*D. rerio*	ctsz.1	D. rerio_ctsz.1
ENSDARG00000099376	*D. rerio*	ctsz.2	D. rerio_ctsz.2
ENSGACG00000000498	*G. aculeatus*	ctsz.1	G. aculeatus_ctsz.1
ENSGACG00000016618	*G. aculeatus*	ctsz.3	G. aculeatus_ctsz.3
ENSGALG00000007462	*G. gallus*	ctsz	G. gallus_ctsz
ENSG00000101160	*H. sapiens*	CTSZ	H. sapiens_CTSZ
ENSLACG00000014649	*L. chalumnae*	ctsz	L. chalumnae_ctsz
ENSLOCG00000006242	*L. oculatus*	ctsz	L. oculatus_ctsz
ENSMUSG00000016256	*M. musculus*	Ctsz	M. musculus_Ctsz
ENSORLG00000025883	*O. latipes*	ctsz.1	O. latipes_ctsz.1
ENSORLG00000003833	*O. latipes*	ctsz.2	O. latipes_ctsz.2
ENSONIG00000019234	*O. niloticus*	ctsz.1	O. niloticus_ctsz.1
ENSONIG00000018462	*O. niloticus*	ctsz.2	O. niloticus_ctsz.2
ENSPFOG00000015501	*P. formosa*	ctsz.1	P. formosa_ctsz.1
ENSPFOG00000018377	*P. formosa*	ctsz.2	P. formosa_ctsz.2
ENSPFOG00000000758	*P. formosa*	ctsz.3	P. formosa_ctsz.3
ENSSAUG00010025140	*S. aurata*	ctsz.1	**S. aurata_ctsz.1**
ENSSAUG00010014606	*S. aurata*	ctsz.2	**S. aurata_ctsz.2**
ENSSAUG00010010858	*S. aurata*	ctsz.3	**S. aurata_ctsz.3**
ENSSDUG00000003690	*S. dumerilii*	ctsz.1	S. dumerilii_ctsz.1
ENSSDUG00000007940	*S. dumerilii*	ctsz.2	S. dumerilii_ctsz.2
ENSSDUG00000008078	*S. dumerilii*	ctsz.3	S. dumerilii_ctsz.3
ENSTNIG00000007076	*T. nigroviridis*	ctsz.1	T. nigroviridis_ctsz.1
ENSTNIG00000013053	*T. nigroviridis*	ctsz.2	T. nigroviridis_ctsz.2
ENSTRUG00000013236	*T. rubripes*	ctsz.1	T. rubripes_ctsz.1
ENSXMAG00000003406	*X. maculatus*	ctsz.1	X. maculatus_ctsz.1
ENSXMAG00000002006	*X. maculatus*	ctsz.2	X. maculatus_ctsz.2
ENSXMAG00000024174	*X. maculatus*	ctsz.3	X. maculatus_ctsz.3

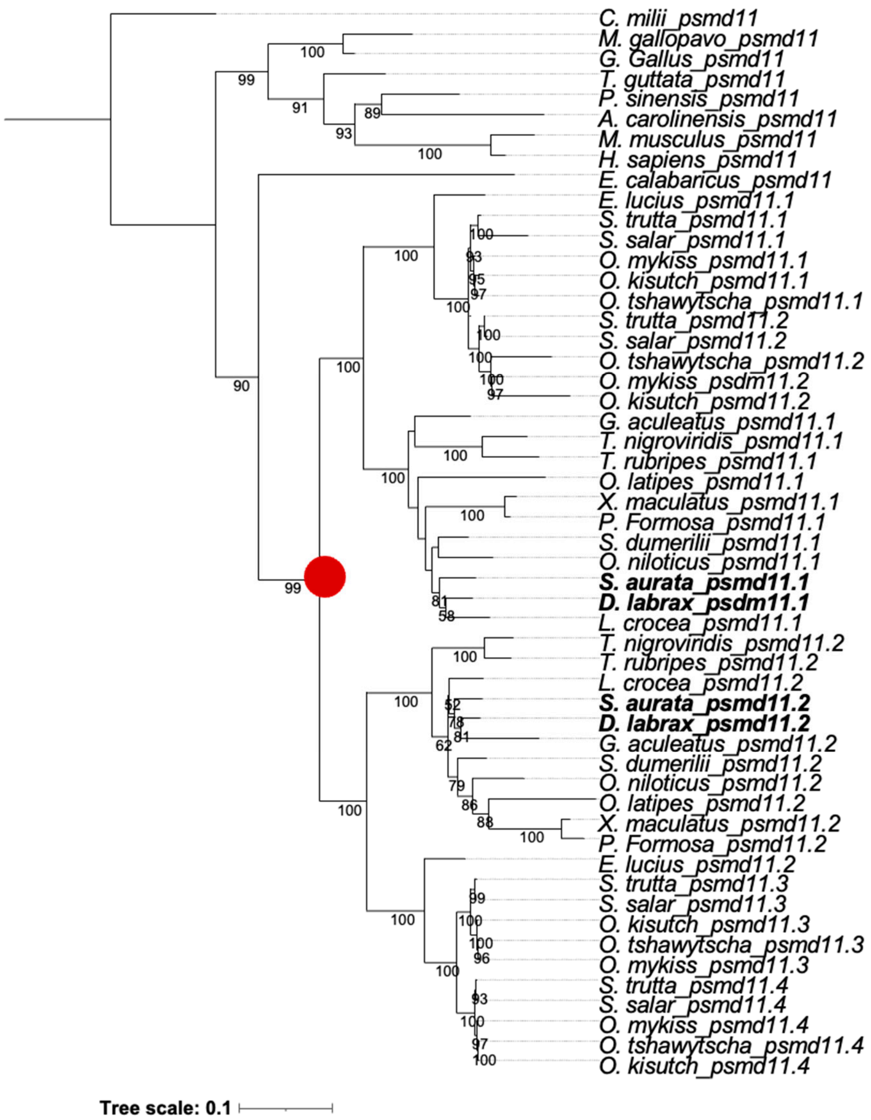

Supplementary Figure S15. Maximum-likelihood (ML) phylogenetic tree of the *psmd11* gene. The red circle indicates the duplication node consistent with the TS-WGD. The substitution model used was GTR+G+I. Numbers at the nodes represent bootstrap values.

Supplementary Table S19. Table showing the correspondence between Ensembl IDs and the names used in the *psmd11* phylogenetic tree

Ensembl ID	species	a/a	Tree ID
ENSACAG00000016508	*A. carolinensis*	*psmd11*	*A. carolinensis_psmd11*
ENSCMIG00000009001	*C. milii*	*psmd11*	*C. milii_psmd11*
ENSDLAG00005023007	*D. labrax*	*psmd11.1*	***D. labrax_psmd11.1***
ENSDLAG00005032181	*D. labrax*	*psmd11.2*	***D. labrax_psmd11.2***
ENSECRG00000011715	*E. calabaricus*	*psmd11*	*E. calabaricus_psmd11*
ENSELUG00000022637	*E. lucius*	*psmd11.1*	*E. lucius_psmd11.1*
ENSELUG00000008204	*E. lucius*	*psmd11.2*	*E. lucius_psmd11.2*
ENSGACG00000005084	*G. aculeatus*	*psmd11.1*	*G. aculeatus_psmd11.1*

ENSGACG00000003795	*G. aculeatus*	*psmd11.2*	*G. aculeatus_psmd11.2*
ENSGALG00010024133	*G. gallus*	*psmd11*	*G. gallus_psmd11*
ENSLCRG00005017156	*L. crocea*	*psmd11.1*	*L. crocea_psmd11.1*
ENSLCRG00005020186	*L. crocea*	*psmd11.2*	*L. crocea_psmd11.2*
ENSMGAG00000006367	*M. gallopavo*	*psmd11*	*M. gallopavo_psmd11*
ENSMUSG00000017428	*M. musculus*	*psmd11*	*M. musculus_psmd11*
ENSOKIG00005002435	*O. kisutch*	*psmd11.1*	*O. kisutch_psmd11.1*
ENSOKIG00005010937	*O. kisutch*	*psmd11.2*	*O. kisutch_psmd11.2*
ENSOKIG00005030783	*O. kisutch*	*psmd11.3*	*O. kisutch_psmd11.3*
ENSOKIG00005050034	*O. kisutch*	*psmd11.4*	*O. kisutch_psmd11.4*
ENSOMYG00000042895	*O. mykiss*	*psmd11.1*	*O. mykiss_psmd11.1*
ENSOMYG00000025835	*O. mykiss*	*psmd11.2*	*O. mykiss_psmd11.2*
ENSOMYG00000046708	*O. mykiss*	*psmd11.3*	*O. mykiss_psmd11.3*
ENSOMYG00000026111	*O. mykiss*	*psmd11.4*	*O. mykiss_psmd11.4*
ENSONIG00000006439	*O. niloticus*	*psmd11.1*	*O. niloticus_psmd11.1*
ENSONIG00000018238	*O. niloticus*	*psmd11.2*	*O. niloticus_psmd11.2*
ENSORLG00000018831	*O. latipes*	*psmd11.1*	*O. latipes_psmd11.1*
ENSORLG00000012079	*O. latipes*	*psmd11.2*	*O. latipes_psmd11.2*
ENSOTSG00005042061	*O. tshawytscha*	*psmd11.1*	*O. tshawytscha_psmd11.1*
ENSOTSG00005046532	*O. tshawytscha*	*psmd11.2*	*O. tshawytscha_psmd11.2*
ENSOTSG00005009598	*O. tshawytscha*	*psmd11.3*	*O. tshawytscha_psmd11.3*
ENSOTSG00005043324	*O. tshawytscha*	*psmd11.4*	*O. tshawytscha_psmd11.4*
ENSPFOG00000014420	*P. formosa*	*psmd11.1*	*P. formosa_psmd11.1*
ENSPFOG00000017872	*P. formosa*	*psmd11.2*	*P. formosa_psmd11.2*
ENSPSIG00000003280	*P. sinensis*	*psmd11*	*P. sinensis_psmd11*
ENSSAUG00010021008	*S. aurata*	*psmd11.1*	***S. aurata_psmd11.1***
ENSSAUG00010026051	*S. aurata*	*psmd11.2*	***S. aurata_psmd11.2***
ENSSDUG00000006787	*S. dumerili*	*psmd11.1*	*S. dumerili_psmd11.1*
ENSSDUG00000010564	*S. dumerili*	*psmd11.2*	*S. dumerili_psmd11.2*
ENSSSAG00000084100	*S. salar*	*psmd11.1*	*S. salar_psmd11.1*
ENSSSAG00000078688	*S. salar*	*psmd11.2*	*S. salar_psmd11.2*
ENSSSAG00000120584	*S. salar*	*psmd11.3*	*S. salar_psmd11.3*
ENSSSAG00000072465	*S. salar*	*psmd11.4*	*S. salar_psmd11.4*
ENSSTUG00000000973	*S. trutta*	*psmd11.1*	*S. trutta_psmd11.1*
ENSSTUG00000009233	*S. trutta*	*psmd11.2*	*S. trutta_psmd11.2*
ENSSTUG00000027628	*S. trutta*	*psmd11.3*	*S. trutta_psmd11.3*
ENSSTUG00000035992	*S. trutta*	*psmd11.4*	*S. trutta_psmd11.4*
ENSTGUG00000002139	*T. guttata*	*psmd11*	*T. guttata_psmd11*
ENSTNIG00000006061	*T. nigroviridis*	*psmd11.1*	*T. nigroviridis_psmd11.1*
ENSTNIG00000011164	*T. nigroviridis*	*psmd11.2*	*T. nigroviridis_psmd11.2*
ENSTRUG00000007262	*T. rubripes*	*psmd11.1*	*T. rubripes_psmd11.1*

ENSTRUG00000002031	*T. rubripes*	*psmd11.2*	*T. rubripes_psmd11.2*
ENSXETG00000029937	*X. tropicalis*	*psmd11*	*X. tropicalis_psmd11*
ENSXMAG00000010693	*X. maculatus*	*psmd11.1*	*X. maculatus_psmd11.1*
ENSXMAG00000017949	*X. maculatus*	*psmd11.2*	*X. maculatus_psmd11.2*
ENSG00000108671	*H. sapiens*	*PSMD11*	*H. sapiens_PSMD11*

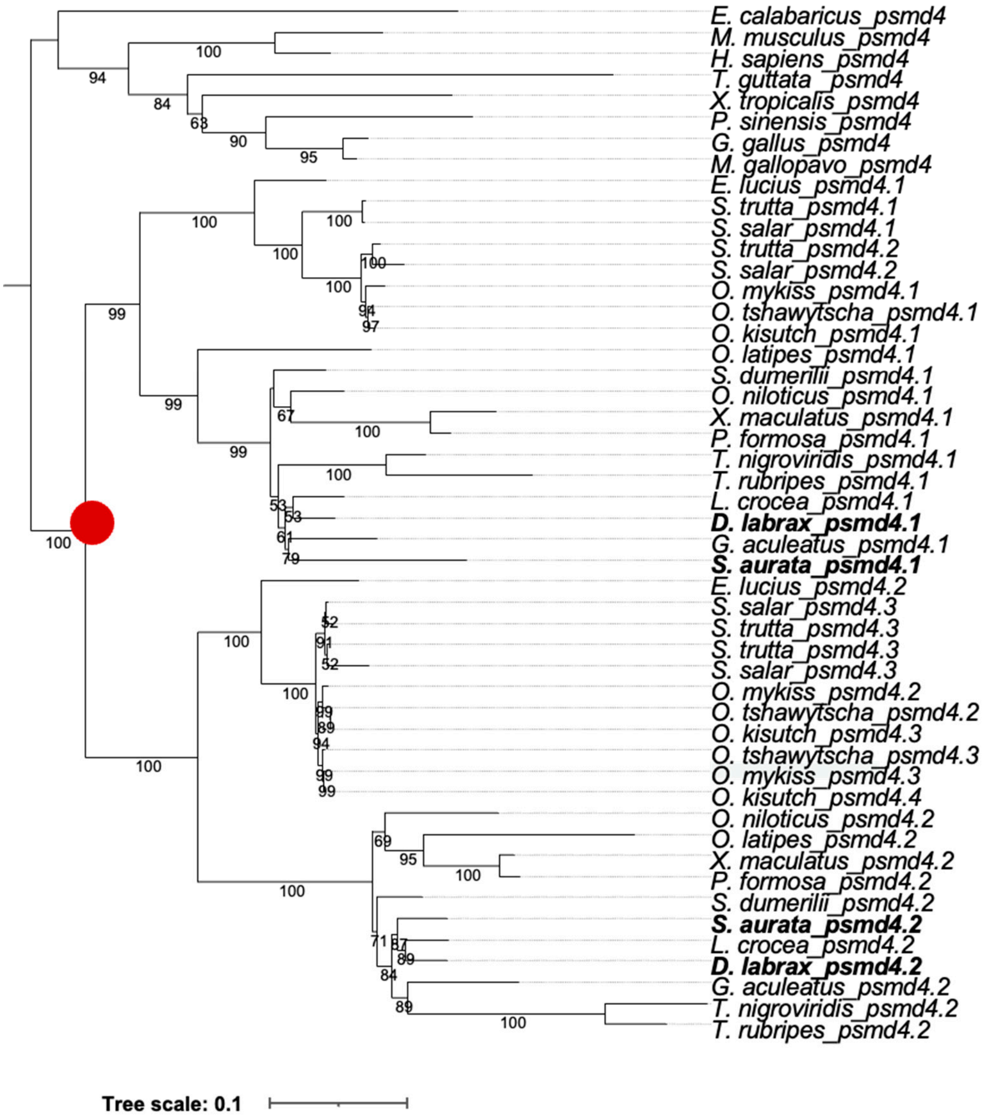

Supplementary Figure S16. Maximum-likelihood (ML) phylogenetic tree of the *psmd4* gene. The red circle indicates the duplication node consistent with the TS-WGD. The substitution model used was GTR+G+I. Numbers at the nodes represent bootstrap values.

Supplementary Table S20. Table showing the correspondence between Ensembl IDs and the names used in the *psmd4* phylogenetic tree

Ensembl ID	species	a/a	Tree ID
ENSCMIG00000000632	*C. milii*	*psmd4*	*C. milii_psmd4*
ENSDLAG00005013701	*D. labrax*	*psmd4.1*	***D. labrax_psmd4.1***
ENSDLAG00005013691	*D. labrax*	*psmd4.2*	***D. labrax_psmd4.2***
ENSECRG00000006611	*E. calabaricus*	*psmd4*	*E. calabaricus_psmd4*
ENSELUG00000011072	*E. lucius*	*psmd4.1*	*E. lucius_psmd4.1*
ENSELUG00000009051	*E. lucius*	*psmd4.2*	*E. lucius_psmd4.2*
ENSGACG00000004332	*G. aculeatus*	*psmd4.1*	*G. aculeatus_psmd4.1*
ENSGACG00000012182	*G. aculeatus*	*psmd4.2*	*G. aculeatus_psmd4.2*
ENSGALG00010028600	*G. gallus*	*psmd4*	*G. gallus_psmd4*
ENSLCRG00005014122	*L. crocea*	*psmd4.1*	*L. crocea_psmd4.1*
ENSLCRG00005020248	*L. crocea*	*psmd4.2*	*L. crocea_psmd4.2*
ENSMGAG00000001020	*M. gallopavo*	*psmd4*	*M. gallopavo_psmd4*
ENSMUSG00000005625	*M. musculus*	*psmd4*	*M. musculus_psmd4*
ENSOKIG00005012420	*O. kisutch*	*psmd4.1*	*O. kisutch_psmd4.1*
ENSOKIG00005012883	*O. kisutch*	*psmd4.2*	*O. kisutch_psmd4.2*
ENSOKIG00005022033	*O. kisutch*	*psmd4.3*	*O. kisutch_psmd4.3*
ENSOKIG00005027823	*O. kisutch*	*psmd4.4*	*O. kisutch_psmd4.4*
ENSOMYG00000041349	*O. mykiss*	*psmd4.1*	*O. mykiss_psmd4.1*
ENSOMYG00000032932	*O. mykiss*	*psmd4.2*	*O. mykiss_psmd4.2*
ENSOMYG00000009256	*O. mykiss*	*psmd4.3*	*O. mykiss_psmd4.3*
ENSOMYG00000002085	*O. mykiss*	*psmd4.4*	*O. mykiss_psmd4.4*
ENSONIG00000006232	*O. niloticus*	*psmd4.1*	*O. niloticus_psmd4.1*
ENSONIG00000019752	*O. niloticus*	*psmd4.2*	*O. niloticus_psmd4.2*
ENSORLG00000000320	*O. latipes*	*psmd4.1*	*O. latipes_psmd4.1*
ENSORLG00000009532	*O. latipes*	*psmd4.2*	*O. latipes_psmd4.2*
ENSOTSG00005023843	*O. tshawytscha*	*psmd4.1*	*O. tshawytscha_psmd4.1*
ENSOTSG00005052338	*O. tshawytscha*	*psmd4.2*	*O. tshawytscha_psmd4.2*
ENSOTSG00005058097	*O. tshawytscha*	*psmd4.3*	*O. tshawytscha_psmd4.3*
ENSOTSG00005003223	*O. tshawytscha*	*psmd4.4*	*O. tshawytscha_psmd4.4*
ENSPFOG00000024342	*P. formosa*	*psmd4.1*	*P. formosa_psmd4.1*
ENSPFOG00000023412	*P. formosa*	*psmd4.2*	*P. formosa_psmd4.2*
ENSPSIG00000017944	*P. sinensis*	*psmd4*	*P. sinensis_psmd4*
ENSSAUG00010012828	*S. aurata*	*psmd4.1*	***S. aurata_psmd4.1***
ENSSAUG00010018503	*S. aurata*	*psmd4.2*	***S. aurata_psmd4.2***
ENSSDUG00000002270	*S. dumerili*	*psmd4.1*	*S. dumerili_psmd4.1*

ENSSDUG00000017529	*S. dumerili*	*psmd4.2*	*S. dumerili_psmd4.2*
ENSSSAG00000038894	*S. salar*	*psmd4.1*	*S. salar_psmd4.1*
ENSSSAG00000046871	*S. salar*	*psmd4.2*	*S. salar_psmd4.2*
ENSSSAG00000004354	*S. salar*	*psmd4.3*	*S. salar_psmd4.3*
ENSSSAG00000092495	*S. salar*	*psmd4.4*	*S. salar_psmd4.4*
ENSSTUG00000029793	*S. trutta*	*psmd4.1*	*S. trutta_psmd4.1*
ENSSTUG00000034773	*S. trutta*	*psmd4.2*	*S. trutta_psmd4.2*
ENSSTUG00000041012	*S. trutta*	*psmd4.3*	*S. trutta_psmd4.3*
ENSSTUG00000047294	*S. trutta*	*psmd4.4*	*S. trutta_psmd4.4*
ENSTGUG00000017424	*T. guttata*	*psmd4*	*T. guttata_psmd4*
ENSTNIG00000017570	*T. nigroviridis*	*psmd4.1*	*T. nigroviridis_psmd4.1*
ENSTNIG00000018343	*T. nigroviridis*	*psmd4.2*	*T. nigroviridis_psmd4.2*
ENSTRUG00000024969	*T. rubripes*	*psmd4.1*	*T. rubripes_psmd4.1*
ENSTRUG00000002109	*T. rubripes*	*psmd4.2*	*T. rubripes_psmd4.2*
ENSXETG00000042857	*X. tropicalis*	*psmd4*	*X. tropicalis_psmd4*
ENSXMAG00000007039	*X. maculatus*	*psmd4.1*	*X. maculatus_psmd4.1*
ENSXMAG00000015234	*X. maculatus*	*psmd4.2*	*X. maculatus_psmd4.2*
ENSG00000159352	*H. sapiens*	*PSMD4*	*H. sapiens_PSMD4*

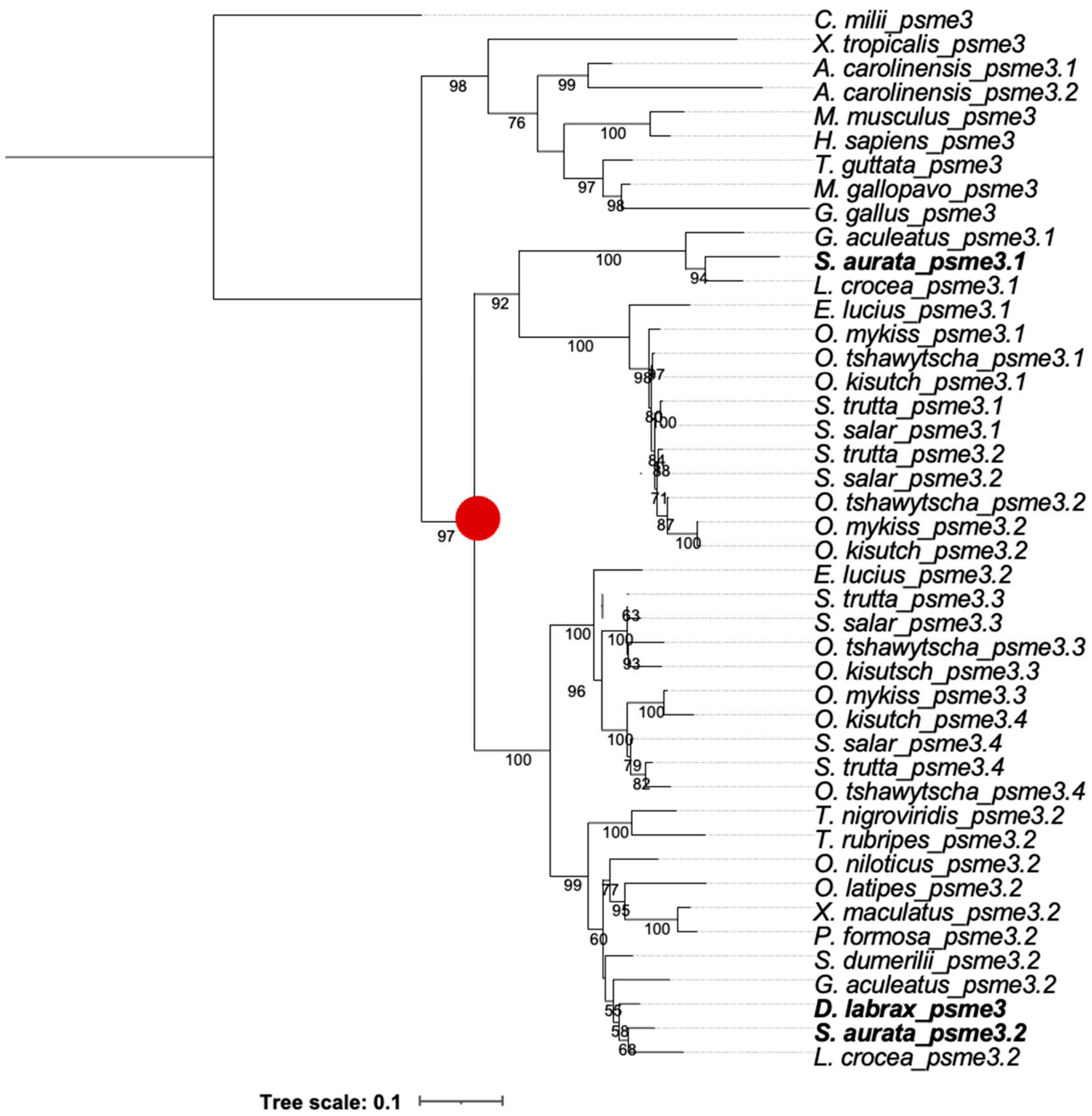

Supplementary Figure S17. Maximum-likelihood (ML) phylogenetic tree of the *psme3* gene. The red circle indicates the duplication node consistent with the TS-WGD. The substitution model used was GTR+G+I. Numbers at the nodes represent bootstrap values.

Supplementary Table S21. Table showing the correspondence between Ensembl IDs and the names used in the *psme3* phylogenetic tree

Ensembl ID	species	a/a	Tree ID
ENSACAG00000007122	*A. carolinensis*	*psme3.1*	*A. carolinensis_psme3.1*
ENSACAG00000013688	*A. carolinensis*	*psme3.2*	*A. carolinensis_psme3.2*
ENSCMIG00000003478	*C. milii*	*psme3*	*C. milii_psme3*
ENSDLAG00005020891	*D. labrax*	*psme3*	***D. labrax_psme3***
ENSECRG00000019810	*E. calabaricus*	*psme3.1*	*E. calabaricus_psme3.1*
ENSECRG00000021334	*E. calabaricus*	*psme3.2*	*E. calabaricus_psme3.2*
ENSELUG00000000273	*E. lucius*	*psme3.1*	*E. lucius_psme3.1*
ENSELUG00000006404	*E. lucius*	*psme3.2*	*E. lucius_psme3.2*

ENSGACG00000005761	*G. aculeatus*	*psme3.1*	*G. aculeatus_psme3.1*
ENSGACG00000003681	*G. aculeatus*	*psme3.2*	*G. aculeatus_psme3.2*
ENSGALG00010022317	*G. gallus*	*psme3*	*G. gallus_psme3*
ENSLCRG00005009551	*L. crocea*	*psme3.1*	*L. crocea_psme3.1*
ENSLCRG00005011376	*L. crocea*	*psme3.2*	*L. crocea_psme3.2*
ENSLOCG00000012181	*L. oculatus*	*psme3*	*L. oculatus_psme3*
ENSMGAG00000004196	*M. gallopavo*	*psme3*	*M. gallopavo_psme3*
ENSMUSG00000078652	*M. musculus*	*psme3*	*M. musculus_psme3*
ENSOKIG00005002942	*O. kisutch*	*psme3.1*	*O. kisutch_psme3.1*
ENSOKIG00005005101	*O. kisutch*	*psme3.2*	*O. kisutch_psme3.2*
ENSOKIG00005007470	*O. kisutch*	*psme3.3*	*O. kisutch_psme3.3*
ENSOKIG00005011203	*O. kisutch*	*psme3.4*	*O. kisutch_psme3.4*
ENSOMYG00000038921	*O. mykiss*	*psme3.1*	*O. mykiss_psme3.1*
ENSOMYG00000043172	*O. mykiss*	*psme3.2*	*O. mykiss_psme3.2*
ENSOMYG00000039962	*O. mykiss*	*psme3.3*	*O. mykiss_psme3.3*
ENSOMYG00000013045	*O. mykiss*	*psme3.4*	*O. mykiss_psme3.4*
ENSONIG00000018267	*O. niloticus*	*psme3*	*O. niloticus_psme3*
ENSORLG00000018778	*O. latipes*	*psme3*	*O. latipes_psme3*
ENSOTSG00005051545	*O. tshawytscha*	*psme3.1*	*O. tshawytscha_psme3.1*
ENSOTSG00005039008	*O. tshawytscha*	*psme3.2*	*O. tshawytscha_psme3.2*
ENSOTSG00005052821	*O. tshawytscha*	*psme3.3*	*O. tshawytscha_psme3.3*
ENSOTSG00005002575	*O. tshawytscha*	*psme3.4*	*O. tshawytscha_psme3.4*
ENSPFOG00000017453	*P. formosa*	*psme3*	*P. formosa_psme3*
ENSSAUG00010007133	*S. aurata*	*psme3.1*	***S. aurata_psme3.1***
ENSSAUG00010021784	*S. aurata*	*psme3.2*	***S. aurata_psme3.2***
ENSSDUG00000011048	*S. dumerili*	*psme3*	*S. dumerili_psme3*
ENSSSAG00000010640	*S. salar*	*psme3.1*	*S. salar_psme3.1*
ENSSSAG00000075257	*S. salar*	*psme3.2*	*S. salar_psme3.2*
ENSSSAG00000076091	*S. salar*	*psme3.3*	*S. salar_psme3.3*
ENSSSAG00000049432	*S. salar*	*psme3.4*	*S. salar_psme3.4*
ENSSTUG00000007920	*S. trutta*	*psme3.1*	*S. trutta_psme3.1*
ENSSTUG00000020913	*S. trutta*	*psme3.2*	*S. trutta_psme3.2*
ENSSTUG00000024841	*S. trutta*	*psme3.3*	*S. trutta_psme3.3*
ENSSTUG00000032585	*S. trutta*	*psme3.4*	*S. trutta_psme3.4*
ENSTGUG00000002797	*T. guttata*	*psme3*	*T. guttata_psme3*
ENSTNIG00000014411	*T. nigroviridis*	*psme3*	*T. nigroviridis_psme3*
ENSTRUG00000006170	*T. rubripes*	*psme3*	*T. rubripes_psme3*
ENSXETG00000040776	*X. tropicalis*	*psme3*	*X. tropicalis_psme3*
ENSXMAG00000027968	*X. maculatus*	*psme3*	*X. maculatus_psme3*
ENSG00000131467	*H. sapiens*	*PSME3*	*H. sapiens_PSME3*

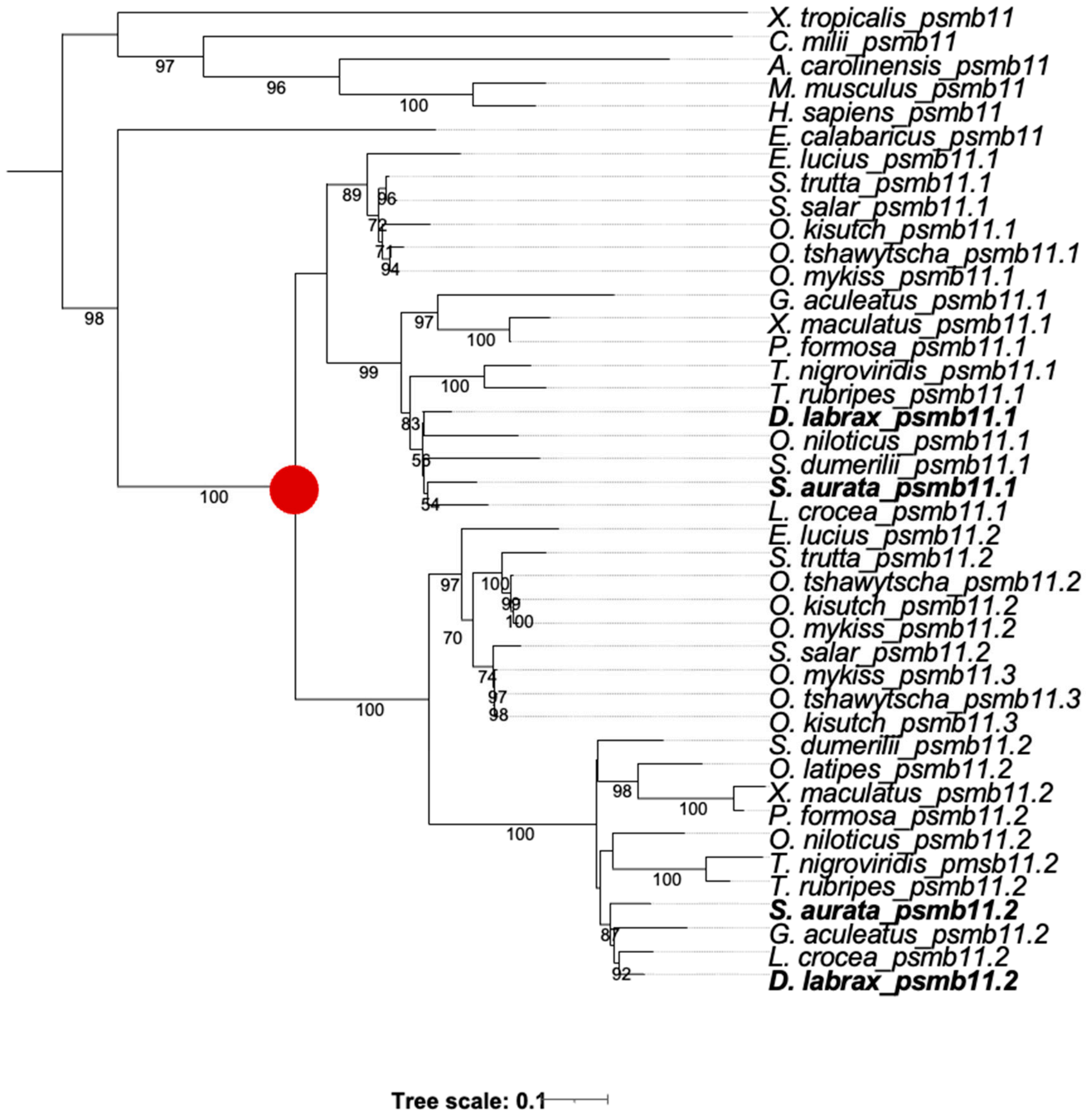

Supplementary Figure S18. Maximum-likelihood (ML) phylogenetic tree of the *psmb11* gene. The red circle indicates the duplication node consistent with the TS-WGD. The substitution model used was GTR+G+I. Numbers at the nodes represent bootstrap values.

Supplementary Table S22. Table showing the correspondence between Ensembl IDs and the names used in the *psmb11* phylogenetic tree

Ensembl ID	species	a/a	Tree ID
ENSACAG00000001963	*A. carolinensis*	*psmb11*	*A. carolinensis_psmb11*
ENSDLAG00005009568	*D. labrax*	*psmb11.1*	***D. labrax_psmb11.1***
ENSDLAG00005018299	*D. labrax*	*psmb11.2*	***D. labrax_psmb11.2***
ENSECRG00000012282	*E. calabaricus*	*psmb11*	*E. calabaricus_psmb11*
ENSELUG00000039514	*E. lucius*	*psmb11.1*	*E. lucius_psmb11.1*
ENSELUG00000004833	*E. lucius*	*psmb11.2*	*E. lucius_psmb11.2*
ENSGACG00000001737	*G. aculeatus*	*psmb11.1*	*G. aculeatus_psmb11.1*
ENSGACG00000023778	*G. aculeatus*	*psmb11.2*	*G. aculeatus_psmb11.2*

ENSLCRG00005001021	*L. crocea*	*psmb11.1*	*L. crocea_psmb11.1*
ENSLCRG00005011085	*L. crocea*	*psmb11.2*	*L. crocea_psmb11.2*
ENSMUSG00000072423	*M. musculus*	*psmb11*	*M. musculus_psmb11*
ENSOKIG00005012152	*O. kisutch*	*psmb11.1*	*O. kisutch_psmb11.1*
ENSOKIG00005039888	*O. kisutch*	*psmb11.2*	*O. kisutch_psmb11.2*
ENSOKIG00005050787	*O. kisutch*	*psmb11.3*	*O. kisutch_psmb11.3*
ENSOMYG00000034199	*O. mykiss*	*psmb11.1*	*O. mykiss_psmb11.1*
ENSOMYG00000077590	*O. mykiss*	*psmb11.2*	*O. mykiss_psmb11.2*
ENSOMYG00000042080	*O. mykiss*	*psmb11.3*	*O. mykiss_psmb11.3*
ENSONIG00000029853	*O. niloticus*	*psmb11.1*	*O. niloticus_psmb11.1*
ENSONIG00000003130	*O. niloticus*	*psmb11.2*	*O. niloticus_psmb11.2*
ENSORLG00000022851	*O. latipes*	*psmb11*	*O. latipes_psmb11*
ENSOTSG00005039307	*O. tshawytscha*	*psmb11.1*	*O. tshawytscha_psmb11.1*
ENSOTSG00005047911	*O. tshawytscha*	*psmb11.2*	*O. tshawytscha_psmb11.2*
ENSOTSG00005040906	*O. tshawytscha*	*psmb11.3*	*O. tshawytscha_psmb11.3*
ENSPFOG00000016979	*P. formosa*	*psmb11.1*	*P. formosa_psmb11.1*
ENSPFOG00000018693	*P. formosa*	*psmb11.2*	*P. formosa_psmb11.2*
ENSSAUG00010015241	*S. aurata*	*psmb11.1*	***S. aurata_psmb11.1***
ENSSAUG00010018769	*S. aurata*	*psmb11.2*	***S. aurata_psmb11.2***
ENSSDUG00000011703	*S. dumerili*	*psmb11.1*	*S. dumerili_psmb11.1*
ENSSDUG00000020267	*S. dumerili*	*psmb11.2*	*S. dumerili_psmb11.2*
ENSSSAG00000090634	*S. salar*	*psmb11.1*	*S. salar_psmb11.1*
ENSSSAG00000066415	*S. salar*	*psmb11.2*	*S. salar_psmb11.2*
ENSSTUG00000005520	*S. trutta*	*psmb11.1*	*S. trutta_psmb11.1*
ENSSTUG00000008182	*S. trutta*	*psmb11.2*	*S. trutta_psmb11.2*
ENSTNIG00000001142	*T. nigroviridis*	*psmb11.1*	*T. nigroviridis_psmb11.1*
ENSTNIG00000006995	*T. nigroviridis*	*psmb11.2*	*T. nigroviridis_psmb11.2*
ENSTRUG00000032620	*T. rubripes*	*psmb11.1*	*T. rubripes_psmb11.1*
ENSTRUG00000013369	*T. rubripes*	*psmb11.2*	*T. rubripes_psmb11.2*
ENSXETG00000022638	*X. tropicalis*	*psmb11*	*X. tropicalis_psmb11*
ENSXMAG00000013873	*X. maculatus*	*psmb11.1*	*X. maculatus_psmb11.1*
ENSXMAG00000000491	*X. maculatus*	*psmb11.2*	*X. maculatus_psmb11.2*
ENSG00000222028	*H. sapiens*	*PSMB11*	*H. sapiens_PSMB11*

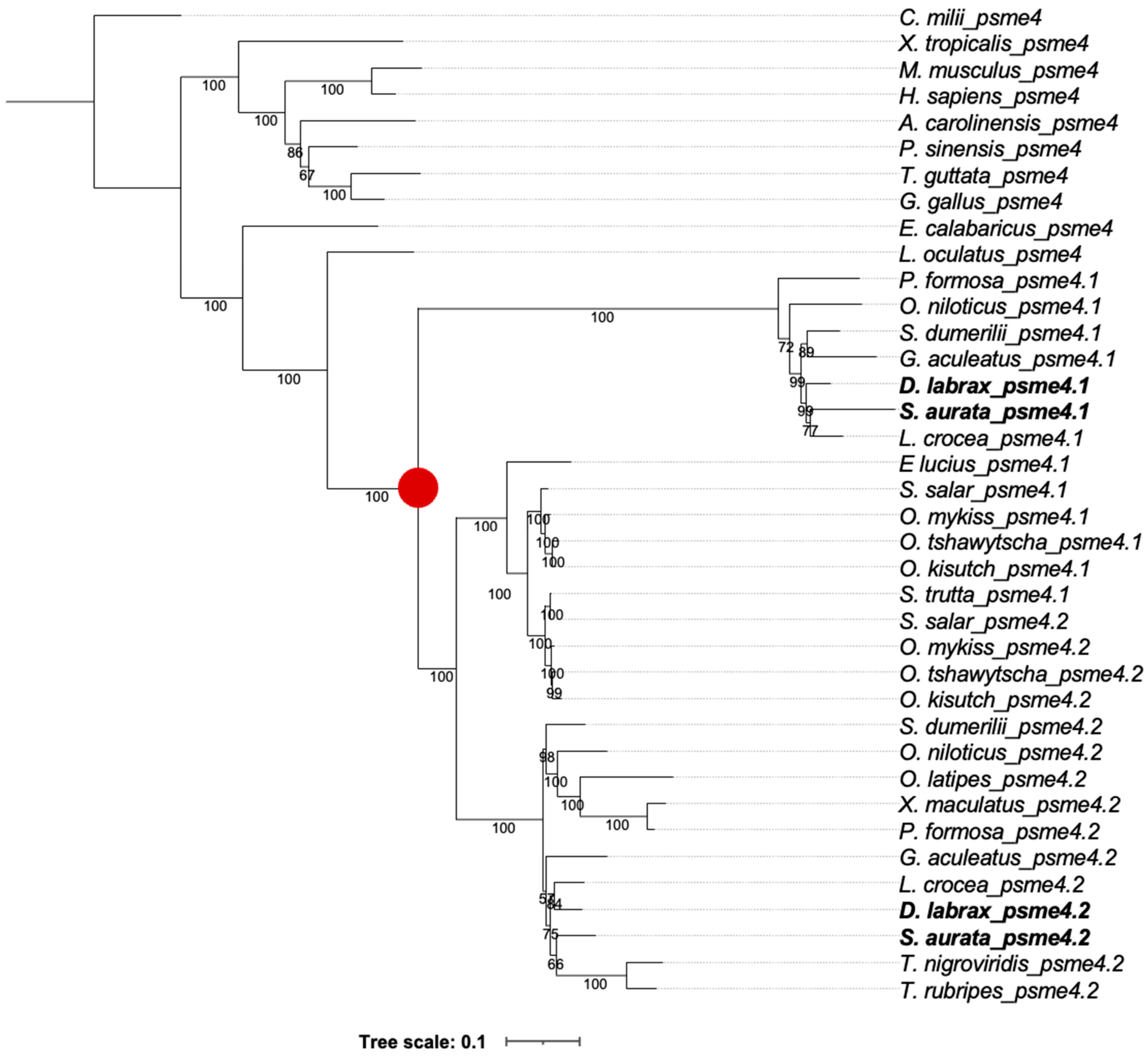

Supplementary Figure S19. Maximum-likelihood (ML) phylogenetic tree of the *psme4* gene. The red circle indicates the duplication node consistent with the TS-WGD. The substitution model used was GTR+G+I. Numbers at the nodes represent bootstrap values.

Supplementary Table S23. Table showing the correspondence between Ensembl IDs and the names used in the *psme4* phylogenetic tree

Ensembl ID	species	a/a	Tree ID
ENSACAG00000012331	*A. carolinensis*	*psme4*	*A. carolinensis_psme4*
ENSCMIG00000004000	*C. milii*	*psme4*	*C. milii_psme4*
ENSDLAG00005020027	*D. labrax*	*psme4.1*	***D. labrax_psme4.1***
ENSDLAG00005014383	*D. labrax*	*psme4.2*	***D. labrax_psme4.2***
ENSECRG00000020210	*E. calabaricus*	*psme4*	*E. calabaricus_psme4*
ENSELUG00000011276	*E. lucius*	*psme4*	*E. lucius_psme4*
ENSGACG00000005686	*G. aculeatus*	*psme4.1*	*G. aculeatus_psme4.1*
ENSGACG00000002691	*G. aculeatus*	*psme4.2*	*G. aculeatus_psme4.2*
ENSGALG00010016938	*G. gallus*	*psme4*	*G. gallus_psme4*

ENSLCRG00005016621	*L. crocea*	*psme4.1*	*L. crocea_psme4.1*
ENSLCRG00005020760	*L. crocea*	*psme4.2*	*L. crocea_psme4.2*
ENSLOCG00000016250	*L. oculatus*	*psme4*	*L. oculatus_psme4*
ENSMGAG00000002484	*M. gallopavo*	*psme4*	*M. gallopavo_psme4*
ENSMUSG00000040850	*M. musculus*	*psme4*	*M. musculus_psme4*
ENSOKIG00005012067	*O. kisutch*	*psme4.1*	*O. kisutch_psme4.1*
ENSOKIG00005036846	*O. kisutch*	*psme4.2*	*O. kisutch_psme4.2*
ENSOMYG00000025175	*O. mykiss*	*psme4.1*	*O. mykiss_psme4.1*
ENSOMYG00000036954	*O. mykiss*	*psme4.2*	*O. mykiss_psme4.2*
ENSONIG00000015943	*O. niloticus*	*psme4.1*	*O. niloticus_psme4.1*
ENSONIG00000009819	*O. niloticus*	*psme4.2*	*O. niloticus_psme4.2*
ENSORLG00000010828	*O. latipes*	*psme4*	*O. latipes_psme4*
ENSOTSG00005026777	*O. tshawytscha*	*psme4.1*	*O. tshawytscha_psme4.1*
ENSOTSG00005029152	*O. tshawytscha*	*psme4.2*	*O. tshawytscha_psme4.2*
ENSPFOG00000006853	*P. formosa*	*psme4.1*	*P. formosa_psme4.1*
ENSPFOG00000014029	*P. formosa*	*psme4.2*	*P. formosa_psme4.2*
ENSPSIG00000010998	*P. sinensis*	*psme4*	*P. sinensis_psme4*
ENSSAUG00010023586	*S. aurata*	*psme4.1*	***S. aurata_psme4.1***
ENSSAUG00010026507	*S. aurata*	*psme4.2*	***S. aurata_psme4.2***
ENSSDUG00000006984	*S. dumerili*	*psme4.1*	*S. dumerili_psme4.1*
ENSSDUG00000016370	*S. dumerili*	*psme4.2*	*S. dumerili_psme4.2*
ENSSSAG00000043370	*S. salar*	*psme4.1*	*S. salar_psme4.1*
ENSSSAG00000071799	*S. salar*	*psme4.2*	*S. salar_psme4.2*
ENSSTUG00000028118	*S. trutta*	*psme4*	*S. trutta_psme4*
ENSTGUG00000007232	*T. guttata*	*psme4*	*T. guttata_psme4*
ENSTNIG00000016594	*T. nigroviridis*	*psme4.1*	*T. nigroviridis_psme4.1*
ENSTNIG00000003254	*T. nigroviridis*	*psme4.2*	*T. nigroviridis_psme4.2*
ENSTRUG00000016378	*T. rubripes*	*psme4*	*T. rubripes_psme4*
ENSXETG00000015195	*X. tropicalis*	*psme4*	*X. tropicalis_psme4*
ENSXMAG00000010781	*X. maculatus*	*psme4*	*X. maculatus_psme4*
ENSG00000068878	*H. sapiens*	*PSME4*	*H. sapiens_PSME4*

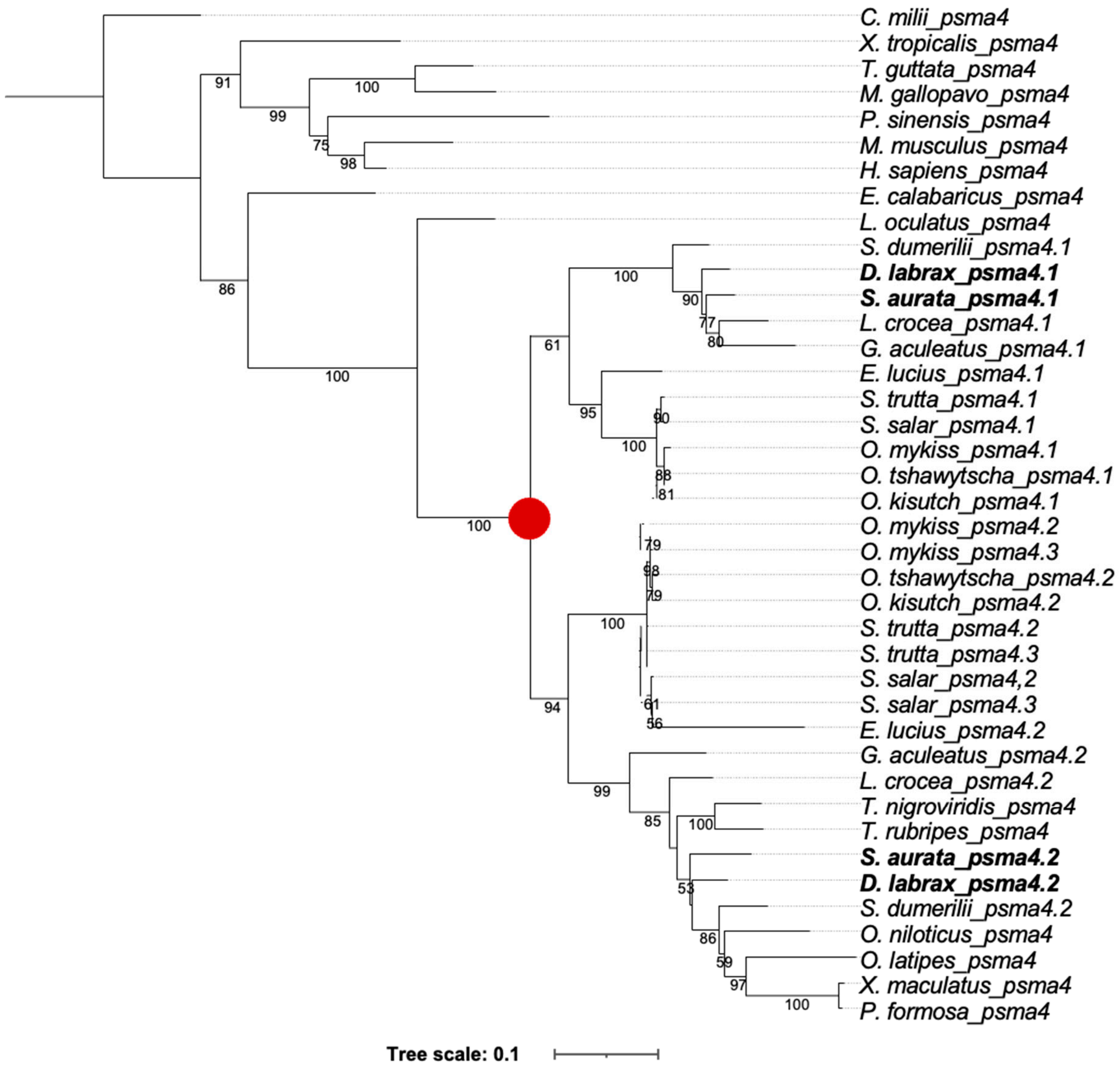

Supplementary Figure S20. Maximum-likelihood (ML) phylogenetic tree of the *psma4* gene. The red circle indicates the duplication node consistent with the TS-WGD. The substitution model used was GTR+G+I. Numbers at the nodes represent bootstrap values.

Supplementary Table S24. Table showing the correspondence between Ensembl IDs and the names used in the *psma4* phylogenetic tree

Ensembl ID	species	a/a	Tree ID
ENSCMIG00000001428	*C. milii*	*psma4*	*C. milii_psma4*
ENSDLAG00005022298	*D. labrax*	*psma4.1*	***D. labrax_psma4.1***
ENSDLAG00005025222	*D. labrax*	*psma4.2*	***D. labrax_psma4.2***
ENSECRG00000021960	*E. calabaricus*	*psma4*	*E. calabaricus_psma4*
ENSELUG00000022757	*E. lucius*	*psma4.1*	*E. lucius_psma4.1*
ENSELUG00000023716	*E. lucius*	*psma4.2*	*E. lucius_psma4.2*
ENSGACG00000007210	*G. aculeatus*	*psma4.1*	*G. aculeatus_psma4.1*
ENSGACG00000017471	*G. aculeatus*	*psma4.2*	*G. aculeatus_psma4.2*
ENSLCRG00005005017	*L. crocea*	*psma4.1*	*L. crocea_psma4.1*

ENSLCRG00005014142	*L. crocea*	*psma4.2*	*L. crocea_psma4.2*
ENSLOCG00000015026	*L. oculatus*	*psma4*	*L. oculatus_psma4*
ENSMGAG00000003682	*M. gallopavo*	*psma4*	*M. gallopavo_psma4*
ENSMUSG00000032301	*M. musculus*	*psma4*	*M. musculus_psma4*
ENSOKIG00005047669	*O. kisutch*	*psma4.1*	*O. kisutch_psma4.1*
ENSOKIG00005048148	*O. kisutch*	*psma4.2*	*O. kisutch_psma4.2*
ENSOMYG00000002105	*O. mykiss*	*psma4.1*	*O. mykiss_psma4.1*
ENSOMYG00000021936	*O. mykiss*	*psma4.2*	*O. mykiss_psma4.2*
ENSOMYG00000024164	*O. mykiss*	*psma4.3*	*O. mykiss_psma4.3*
ENSONIG00000005774	*O. niloticus*	*psma4*	*O. niloticus_psma4*
ENSORLG00000001486	*O. latipes*	*psma4*	*O. latipes_psma4*
ENSOTSG00005010697	*O. tshawytscha*	*psma4.1*	*O. tshawytscha_psma4.1*
ENSOTSG00005061488	*O. tshawytscha*	*psma4.2*	*O. tshawytscha_psma4.2*
ENSPFOG00000016093	*P. formosa*	*psma4*	*P. formosa_psma4*
ENSPSIG00000007470	*P. sinensis*	*psma4*	*P. sinensis_psma4*
ENSSAUG00010003232	*S. aurata*	*psma4.1*	***S. aurata_psma4.1***
ENSSAUG00010017501	*S. aurata*	*psma4.2*	***S. aurata_psma4.2***
ENSSDUG00000000861	*S. dumerili*	*psma4.1*	*S. dumerili_psma4.1*
ENSSDUG00000023519	*S. dumerili*	*psma4.2*	*S. dumerili_psma4.2*
ENSSSAG00000099440	*S. salar*	*psma4.1*	*S. salar_psma4.1*
ENSSSAG00000046820	*S. salar*	*psma4.2*	*S. salar_psma4.2*
ENSSSAG00000091971	*S. salar*	*psma4.3*	*S. salar_psma4.3*
ENSSTUG00000029163	*S. trutta*	*psma4.1*	*S. trutta_psma4.1*
ENSSTUG00000036080	*S. trutta*	*psma4.2*	*S. trutta_psma4.2*
ENSSTUG00000043904	*S. trutta*	*psma4.3*	*S. trutta_psma4.3*
ENSTGUG00000023810	*T. guttata*	*psma4*	*T. guttata_psma4*
ENSTNIG00000009571	*T. nigroviridis*	*psma4*	*T. nigroviridis_psma4*
ENSTRUG00000016631	*T. rubripes*	*psma4*	*T. rubripes_psma4*
ENSXETG00000008877	*X. tropicalis*	*psma4*	*X. tropicalis_psma4*
ENSXMAG00000011091	*X. maculatus*	*psma4*	*X. maculatus_psma4*
ENSG00000041357	*H. sapiens*	*PSMA4*	*H. sapiens_PSMA4*

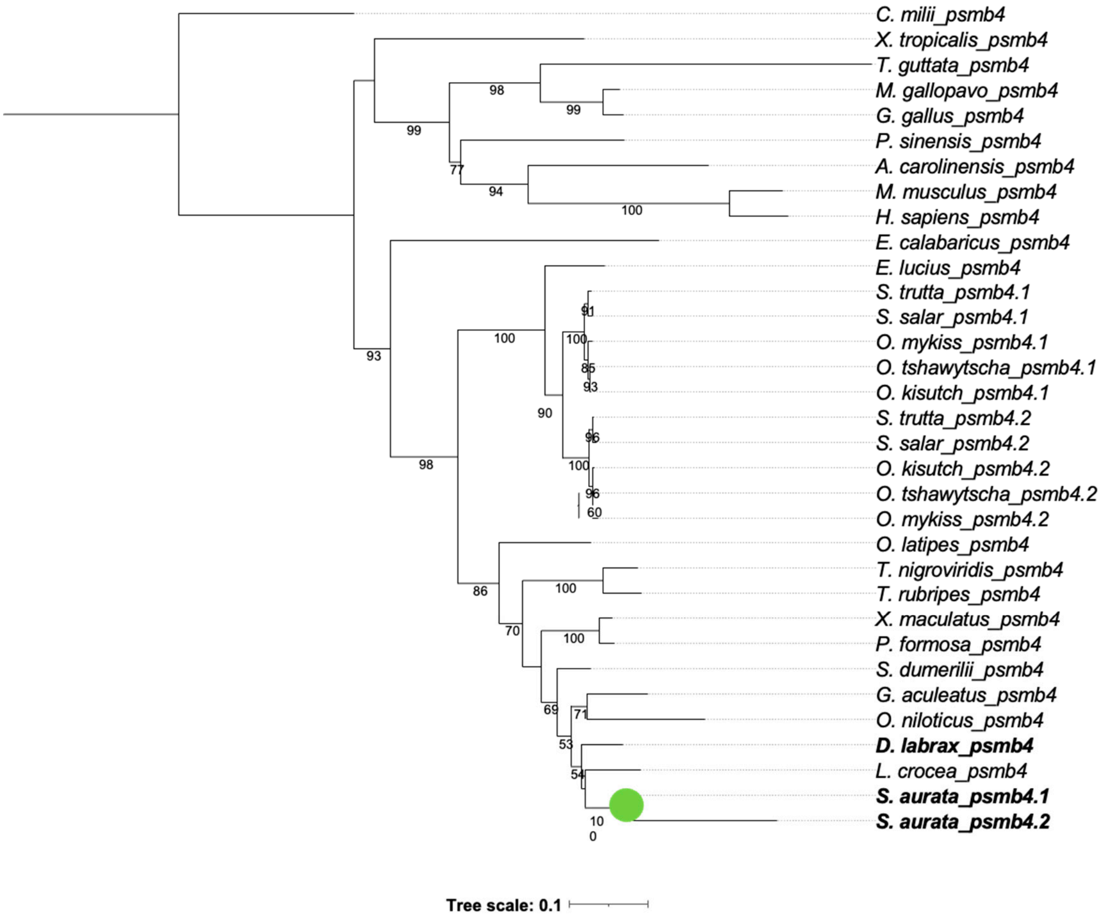

Supplementary Figure S21. Maximum-likelihood (ML) phylogenetic tree of the *psmb4* gene. The green circle indicates a single lineage duplication node. The substitution model used was GTR+G+I. Numbers at the nodes represent bootstrap values.

Supplementary Table S25. Table showing the correspondence between Ensembl IDs and the names used in the *psmb4* phylogenetic tree

Ensembl ID	species	a/a	Tree ID
ENSACAG00000011542	*A. carolinensis*	*psmb4*	*A. carolinensis_psmb4*
ENSCMIG00000003063	*C. milii*	*psmb4*	*C. milii_psmb4*
ENSDLAG00005025170	*D. labrax*	*psmb4*	***D. labrax_psmb4***
ENSECRG00000005432	*E. calabaricus*	*psmb4*	*E. calabaricus_psmb4*
ENSELUG00000016945	*E. lucius*	*psmb4*	*E. lucius_psmb4*
ENSGACG00000009889	*G. aculeatus*	*psmb4*	*G. aculeatus_psmb4*
ENSGALG00010027985	*G. gallus*	*psmb4*	*G. gallus_psmb4*
ENSLCRG00005001103	*L. crocea*	*psmb4*	*L. crocea_psmb4*
ENSMGAG00000001240	*M. gallopavo*	*psmb4*	*M. gallopavo_psmb4*
ENSMUSG00000005779	*M. musculus*	*psmb4*	*M. musculus_psmb4*
ENSOKIG00005012612	*O. kisutch*	*psmb4.1*	*O. kisutch_psmb4.1*
ENSOKIG00005035450	*O. kisutch*	*psmb4.2*	*O. kisutch_psmb4.2*
ENSOMYG00000027735	*O. mykiss*	*psmb4.1*	*O. mykiss_psmb4.1*
ENSOMYG00000017508	*O. mykiss*	*psmb4.2*	*O. mykiss_psmb4.2*
ENSONIG00000003956	*O. niloticus*	*psmb4*	*O. niloticus_psmb4*

ENSORLG00000001283	*O. latipes*	*psmb4*	*O. latipes_psmb4*
ENSOTSG00005045867	*O. tshawytscha*	*psmb4.1*	*O. tshawytscha_psmb4.1*
ENSOTSG00005006266	*O. tshawytscha*	*psmb4.2*	*O. tshawytscha_psmb4.2*
ENSPFOG00000010537	*P. formosa*	*psmb4*	*P. formosa_psmb4*
ENSPSIG00000005631	*P. sinensis*	*psmb4*	*P. sinensis_psmb4*
ENSSAUG00010007698	*S. aurata*	*psmb4.1*	***S. aurata_psmb4.1***
ENSSAUG00010007708	*S. aurata*	*psmb4.2*	***S. aurata_psmb4.2***
ENSSDUG00000001831	*S. dumerili*	*psmb4*	*S. dumerili_psmb4*
ENSSSAG00000069437	*S. salar*	*psmb4.1*	*S. salar_psmb4.1*
ENSSSAG00000040480	*S. salar*	*psmb4.2*	*S. salar_psmb4.2*
ENSSTUG00000020779	*S. trutta*	*psmb4.1*	*S. trutta_psmb4.1*
ENSSTUG00000045103	*S. trutta*	*psmb4.2*	*S. trutta_psmb4.2*
ENSTGUG00000017395	*T. guttata*	*psmb4*	*T. guttata_psmb4*
ENSTNIG00000005387	*T. nigroviridis*	*psmb4*	*T. nigroviridis_psmb4*
ENSTRUG00000000158	*T. rubripes*	*psmb4*	*T. rubripes_psmb4*
ENSXETG00000036566	*X. tropicalis*	*psmb4*	*X. tropicalis_psmb4*
ENSXMAG00000025950	*X. maculatus*	*psmb4*	*X. maculatus_psmb4*
ENSG00000159377	*H. sapiens*	*PSMB4*	*H. sapiens_PSMB4*

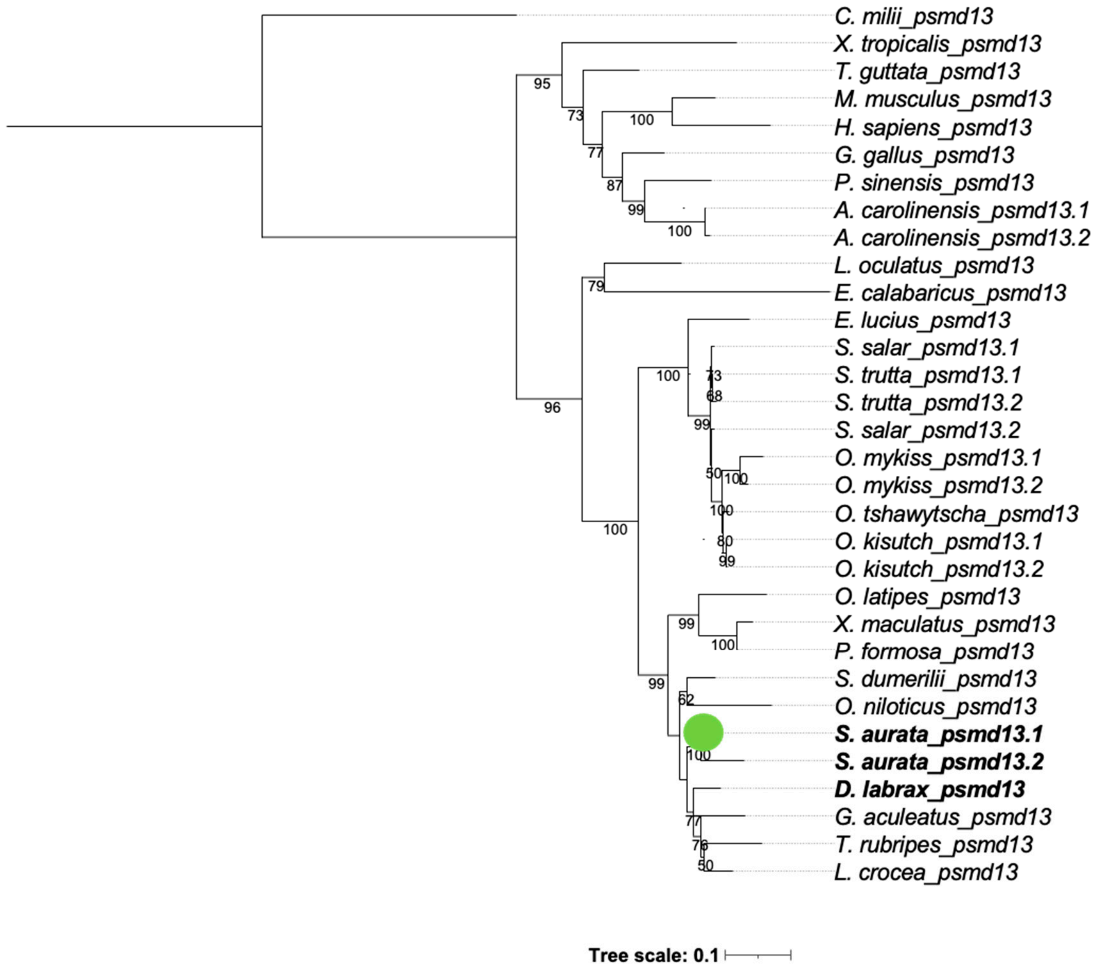

Supplementary Figure S22. Maximum-likelihood (ML) phylogenetic tree of the *psmd13* gene. The green circle indicates a single lineage duplication node. The substitution model used was GTR+G+I. Numbers at the nodes represent bootstrap values.

Supplementary Table S26. Table showing the correspondence between Ensembl IDs and the names used in the *psmd13* phylogenetic tree

Ensembl ID	species	a/a	Tree ID
ENSACAG00000013643	*A. carolinensis*	*psmd13.1*	*A. carolinensis_psmd13.1*
ENSACAG00000017497	*A. carolinensis*	*psmd13.2*	*A. carolinensis_psmd13.2*
ENSCMIG00000001380	*C. milii*	*psmd13.1*	*C. milii_psmd13*
ENSDLAG00005022275	*D. labrax*	*psmd13*	***D. labrax_psmd13***
ENSECRG00000000984	*E. calabaricus*	*psmd13*	*E. calabaricus_psmd13*
ENSELUG00000018515	*E. lucius*	*psmd13*	*E. lucius_psmd13*
ENSGACG00000015724	*G. aculeatus*	*psmd13*	*G. aculeatus_psmd13*
ENSGALG00010020894	*G. gallus*	*psmd13*	*G. gallus_psmd13*
ENSLCRG00005019383	*L. crocea*	*psmd13*	*L. crocea_psmd13*
ENSLOCG00000002439	*L. oculatus*	*psmd13.1*	*L. oculatus_psmd13*
ENSMGAG00000002471	*M. gallopavo*	*psmd13*	*M. gallopavo_psmd13*
ENSMUSG00000025487	*M. musculus*	*psmd13*	*M. musculus_psmd13*
ENSOKIG00005019710	*O. kisutch*	*psmd13.1*	*O. kisutch_psmd13.1*
ENSOKIG00005030286	*O. kisutch*	*psmd13.2*	*O. kisutch_psmd13.2*
ENSOMYG00000030436	*O. mykiss*	*psmd13.1*	*O. mykiss_psmd13.1*
ENSOMYG00000062350	*O. mykiss*	*psmd13.2*	*O. mykiss_psmd13.2*
ENSONIG00000015587	*O. niloticus*	*psmd13*	*O. niloticus_psmd13*
ENSORLG00000000084	*O. latipes*	*psmd13*	*O. latipes_psmd13*
ENSOTSG00005062380	*O. tshawytscha*	*psmd13*	*O. tshawytscha_psmd13*
ENSPFOG00000001193	*P. formosa*	*psmd13*	*P. formosa_psmd13*
ENSPSIG00000006897	*P. sinensis*	*psmd13*	*P. sinensis_psmd13*
ENSSAUG00010016828	*S. aurata*	*psmd13.1*	***S. aurata_psmd13.1***
ENSSAUG00010018216	*S. aurata*	*psmd13.2*	***S. aurata_psmd13.2***
ENSSDUG00000019878	*S. dumerili*	*psmd13*	*S. dumerili_psmd13*
ENSSSAG00000009794	*S. salar*	*psmd13.1*	*S. salar_psmd13.1*
ENSSSAG00000105548	*S. salar*	*psmd13.2*	*S. salar_psmd13.2*
ENSSTUG00000001588	*S. trutta*	*psmd13.1*	*S. trutta_psmd13.1*
ENSSTUG00000035024	*S. trutta*	*psmd13.2*	*S. trutta_psmd13.2*
ENSTGUG00000006851	*T. guttata*	*psmd13*	*T. guttata_psmd13*
ENSTRUG00000012282	*T. rubripes*	*psmd13*	*T. rubripes_psmd13*
ENSXETG00000008827	*X. tropicalis*	*psmd13*	*X. tropicalis_psmd13*
ENSXMAG00000018394	*X. maculatus*	*psmd13*	*X. maculatus_psmd13*
ENSG00000185627	*H. sapiens*	*PSMD13*	*H. sapiens_PSMD13*

Supplementary Table S27. Table showing the correspondence between Ensembl IDs and the names used in the *psma6* phylogenetic tree

Ensembl ID	species	a/a	Tree ID
ENSCMIG00000002150	*C. milii*	*psma6*	*C. milii_psma6*
ENSDLAG00005023268	*D. labrax*	*psma6.1*	*D. labrax_psma6.1*
ENSDLAG00005016118	*D. labrax*	*psma6.2*	*D. labrax_psma6.2*
ENSDLAG00005033478	*D. labrax*	*psma6.3*	*D. labrax_psma6.3*
ENSECRG00000002375	*E. calabaricus*	*psma6.1*	*E. calabaricus_psma6.1*
ENSECRG00000014025	*E. calabaricus*	*psma6.2*	*E. calabaricus_psma6.2*
ENSELUG00000005656	*E. lucius*	*psma6.1*	*E. lucius_psma6.1*
ENSELUG00000013006	*E. lucius*	*psma6.2*	*E. lucius_psma6.2*
ENSELUG00000019641	*E. lucius*	*psma6.3*	*E. lucius_psma6.3*
ENSGACG00000005022	*G. aculeatus*	*psma6.1*	*G. aculeatus_psma6.1*
ENSGACG00000012527	*G. aculeatus*	*psma6.2*	*G. aculeatus_psma6.2*
ENSGALG00010006804	*G. gallus*	*psma6*	*G. gallus_psma6*
ENSLCRG00005010496	*L. crocea*	*psma6.1*	*L. crocea_psma6.1*
ENSLCRG00005015849	*L. crocea*	*psma6.2*	*L. crocea_psma6.2*
ENSLCRG00005022510	*L. crocea*	*psma6.3*	*L. crocea_psma6.3*
ENSLOCG00000009164	*L. oculatus*	*psma6.1*	*L. oculatus_psma6.1*
ENSLOCG00000014186	*L. oculatus*	*psma6.2*	*L. oculatus_psma6.2*
ENSMGAG00000012499	*M. gallopavo*	*psma6*	*M. gallopavo_psma6*
ENSMUSG00000021024	*M. musculus*	*psma6*	*M. musculus_psma6*
ENSOKIG00005017010	*O. kisutch*	*psma6.1*	*O. kisutch_psma6.1*
ENSOKIG00005018423	*O. kisutch*	*psma6.2*	*O. kisutch_psma6.2*
ENSOKIG00005024906	*O. kisutch*	*psma6.3*	*O. kisutch_psma6.3*
ENSOKIG00005032399	*O. kisutch*	*psma6.4*	*O. kisutch_psma6.4*
ENSOMYG00000039553	*O. mykiss*	*psma6.1*	*O. mykiss_psma6.1*
ENSOMYG00000040397	*O. mykiss*	*psma6.2*	*O. mykiss_psma6.2*
ENSOMYG00000036301	*O. mykiss*	*psma6.3*	*O. mykiss_psma6.3*
ENSOMYG00000003459	*O. mykiss*	*psma6.4*	*O. mykiss_psma6.4*
ENSOMYG00000034177	*O. mykiss*	*psma6.5*	*O. mykiss_psma6.5*
ENSOMYG00000047309	*O. mykiss*	*psma6.6*	*O. mykiss_psma6.6*
ENSONIG00000016632	*O. niloticus*	*psma6.1*	*O. niloticus_psma6.1*
ENSONIG00000019500	*O. niloticus*	*psma6.2*	*O. niloticus_psma6.2*
ENSONIG00000038039	*O. niloticus*	*psma6.3*	*O. niloticus_psma6.3*
ENSORLG00000008691	*O. latipes*	*psma6.1*	*O. latipes_psma6.1*
ENSORLG00000015868	*O. latipes*	*psma6.2*	*O. latipes_psma6.2*
ENSOTSG00005003898	*O. tshawytscha*	*psma6.1*	*O. tshawytscha_psma6.1*
ENSOTSG00005009303	*O. tshawytscha*	*psma6.2*	*O. tshawytscha_psma6.2*
ENSOTSG00005046968	*O. tshawytscha*	*psma6.3*	*O. tshawytscha_psma6.3*
ENSOTSG00005024645	*O. tshawytscha*	*psma6.4*	*O. tshawytscha_psma6.4*
ENSOTSG00005032296	*O. tshawytscha*	*psma6.5*	*O. tshawytscha_psma6.5*
ENSG00000100902	*H. sapiens*	*PSMA6*	*H. sapiens_PSMA6*
ENSPFOG00000012070	*P. formosa*	*psma6.1*	*P. formosa_psma6.1*
ENSPFOG00000002628	*P. formosa*	*psma6.2*	*P. formosa_psma6.2*
ENSPFOG00000013811	*P. formosa*	*psma6.3*	*P. formosa_psma6.3*
ENSPSIG00000002493	*P. sinensis*	*psma6.1*	*P. sinensis_psma6.1*
ENSPSIG00000008904	*P. sinensis*	*psma6.2*	*P. sinensis_psma6.2*
ENSSAUG00010006771	*S. aurata*	*psma6.1*	*S. aurata_psma6.1*
ENSSAUG00010011210	*S. aurata*	*psma6.2*	*S. aurata_psma6.2*

ENSSAUG00010021478	*S. aurata*	*psma6.3*	*S. aurata_psma6.3*
ENSSDUG00000006838	*S. dumerili*	*psma6.1*	*S. dumerili_psma6.1*
ENSSDUG00000010613	*S. dumerili*	*psma6.2*	*S. dumerili_psma6.2*
ENSSSAG00000040909	*S. salar*	*psma6.1*	*S. salar_psma6.1*
ENSSSAG00000049820	*S. salar*	*psma6.2*	*S. salar_psma6.2*
ENSSSAG00000044595	*S. salar*	*psma6.3*	*S. salar_psma6.3*
ENSSSAG00000052764	*S. salar*	*psma6.4*	*S. salar_psma6.4*
ENSSSAG00000002721	*S. salar*	*psma6.5*	*S. salar_psma6.5*
ENSSTUG00000003711	*S. trutta*	*psma6.1*	*S. trutta_psma6.1*
ENSSTUG00000010190	*S. trutta*	*psma6.2*	*S. trutta_psma6.2*
ENSSTUG00000018066	*S. trutta*	*psma6.3*	*S. trutta_psma6.3*
ENSSTUG00000025636	*S. trutta*	*psma6.4*	*S. trutta_psma6.4*
ENSSTUG00000037809	*S. trutta*	*psma6.5*	*S. trutta_psma6.5*
ENSSTUG00000040766	*S. trutta*	*psma6.6*	*S. trutta_psma6.6*
ENSTGUG00000011976	*T. guttata*	*psma6*	*T. guttata_psma6*
ENSTNIG00000014333	*T. nigroviridis*	*psma6.1*	*T. nigroviridis_psma6.1*
ENSTNIG00000009709	*T. nigroviridis*	*psma6.2*	*T. nigroviridis_psma6.2*
ENSTRUG00000032693	*T. rubripes*	*psma6.1*	*T. rubripes_psma6.1*
ENSTRUG00000022537	*T. rubripes*	*psma6.2*	*T. rubripes_psma6.2*
ENSTRUG00000020871	*T. rubripes*	*psma6.3*	*T. rubripes_psma6.3*
ENSXETG00000025884	*X. tropicalis*	*psma6*	*X. tropicalis_psma6*
ENSXMAG00000011738	*X. maculatus*	*psma6.1*	*X. maculatus_psma6.1*
ENSXMAG00000002605	*X. maculatus*	*psma6.2*	*X. maculatus_psma6.2*
ENSXMAG00000028877	*X. maculatus*	*psma6.3*	*X. maculatus_psma6.3*

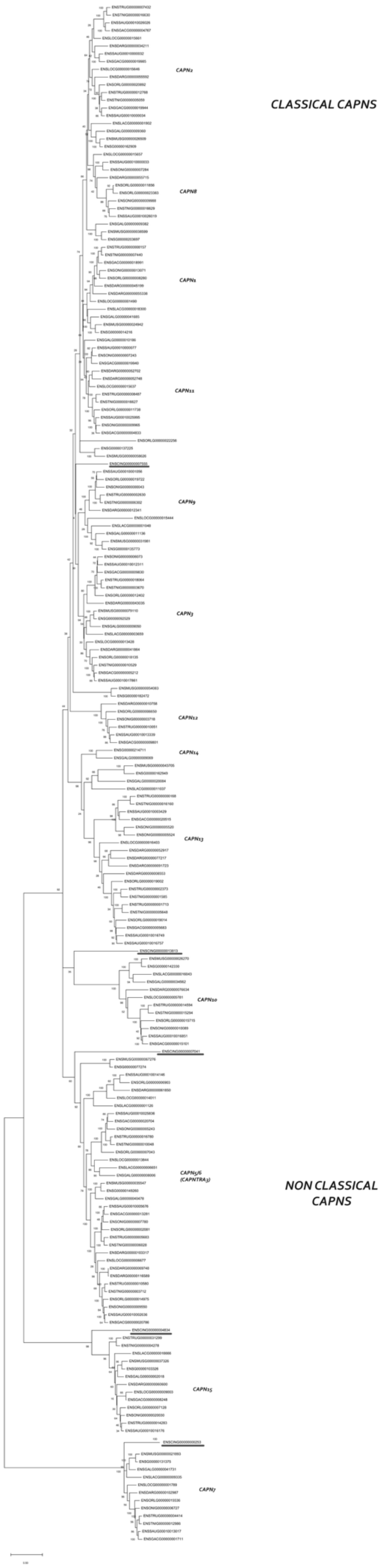
CLASSICAL CAPNS
CAPN2
CAPN8
CAPN1
CAPN11
CAPN9
CAPN3
CAPN12
CAPN14
CAPN13
CAPN10
CAPN5/6
(CAPNTRA3)
NON CLASSICAL
CAPNS
CAPN15
CAPN7

Supplementary Figure S23. Maximum-likelihood (ML) phylogenetic tree of the calpain family. The underlined *Ciona intestinalis* sequences indicate the five urochordate calpain-family genes identified in the analysis. Their clustering within the major calpain subgroups suggests that these lineages were already present before vertebrate whole-genome duplications, whereas the broader expansion of the calpain repertoire occurred later in vertebrates.

Supplementary Table S28. Detailed sequencing quality-control and mapping statistics for both species for each pooled sample.

Species	Sample name	Raw read pairs	Clean read pairs	Raw bases	Clean bases	Error rate(%)	Q20(%)	Q30(%)	GC content(%)
Dicentrarchus labrax	DLFF	30988451	30246645	9.3G	9.1G	0.03	97.79	93.69	47.34
	DLFL	33666672	32152948	10.1G	9.6G	0.03	97.34	92.61	47.74
	DLMM	45521814	43726235	13.7G	13.1G	0.03	97.83	93.89	50.33

	Sample name	Total reads	Total mapped reads	Uniquely mapped reads	Multiple mapped reads	Total mapping rate	Uniquely mapping rate	Multiple mapping rate
Dicentrarchus labrax	DLFF	60,493,290	49,959,673	48,412,541	1,547,132	82.59%	80.03%	2.56%
	DLFL	64,305,896	55,817,583	54,031,293	1,786,290	86.80%	84.02%	2.78%
	DLMM	87,452,470	73,381,791	69,946,459	3,435,332	83.91%	79.98%	3.93%

	Sample name	Raw read pairs	Clean read pairs	Raw bases	Clean bases	Error rate(%)	Q20(%)	Q30(%)	GC content(%)
Sparus aurata	SAFF	30496169	29662496	9.1G	8.9G	0.03	97.69	93.57	49.7
	SAFL	35152628	34398391	10.5G	10.3G	0.03	97.77	93.77	50.39
	SAMM	36265851	35494824	10.9G	10.6G	0.03	97.79	93.83	51.1

	Sample name	Total reads	Total mapped reads	Uniquely mapped reads	Multiple mapped reads	Total mapping rate	Uniquely mapping rate	Multiple mapping rate
Sparus aurata	SAFF	59,324,992	52,664,352	50,363,173	2,301,179	88.77%	84.89%	3.88%
	SAFL	68,796,782	60,961,304	58,328,096	2,633,208	88.61%	84.78%	3.83%
	SAMM	70,989,648	64,058,029	60,274,717	3,783,312	90.24%	84.91%	5.33%

Supplementary Table S29. Coordinates of *capn1*, *capn2*, *capn8*, and *capn11* in *Dicentrarchus labrax* and *Sparus aurata* retrieved from Ensembl.

Species	Gene label	Ensembl gene ID	Sequence region	Genomic location
Dicentrarchus labrax	*capn1*	ENSDLAG00005017924	HG916843.1	HG916843.1:13375764-13398148:+
Dicentrarchus labrax	*capn2.1*	ENSDLAG00005002296	HG916851.1	HG916851.1:11545857-11546643:+
Dicentrarchus labrax	*capn2.2*	ENSDLAG00005000590	HG916828.1	HG916828.1:6388219-6396655:-
Dicentrarchus labrax	*capn8.1*	ENSDLAG00005000702	HG916828.1	HG916828.1:6388337-6402019:-
Dicentrarchus labrax	*capn11.1*	ENSDLAG00005001439	HG916828.1	HG916828.1:6405047-6415428:+
Dicentrarchus labrax	*capn11.2*	ENSDLAG00005016201	HG916851.1	HG916851.1:93603578-93606809:-
Sparus aurata	*capn1*	ENSSAUG00010007897	10	10:17010863-17034115:-
Sparus aurata	*capn2.1*	ENSSAUG00010000032	1	1:556240-569569:-
Sparus aurata	*capn2.2*	ENSSAUG00010026026	15	15:12225762-12233668:+
Sparus aurata	*capn2.3*	ENSSAUG00010000030	1	1:517460-528947:-
Sparus aurata	*capn2.4*	ENSSAUG00010006640	1	1:816028-830656:+
Sparus aurata	*capn2.5*	ENSSAUG00010000034	1	1:585570-599930:+
Sparus aurata	*capn8.1*	ENSSAUG00010000033	1	1:573777-583154:-
Sparus aurata	*capn8.2*	ENSSAUG00010006205	1	1:804142-813892:-
Sparus aurata	*capn11.1*	ENSSAUG00010000077	1	1:604745-622614:+
Sparus aurata	*capn11.2*	ENSSAUG00010008141	1	1:834287-849008:+
Sparus aurata	*capn11.3*	ENSSAUG00010025995	15	15:12199505-12209301:-

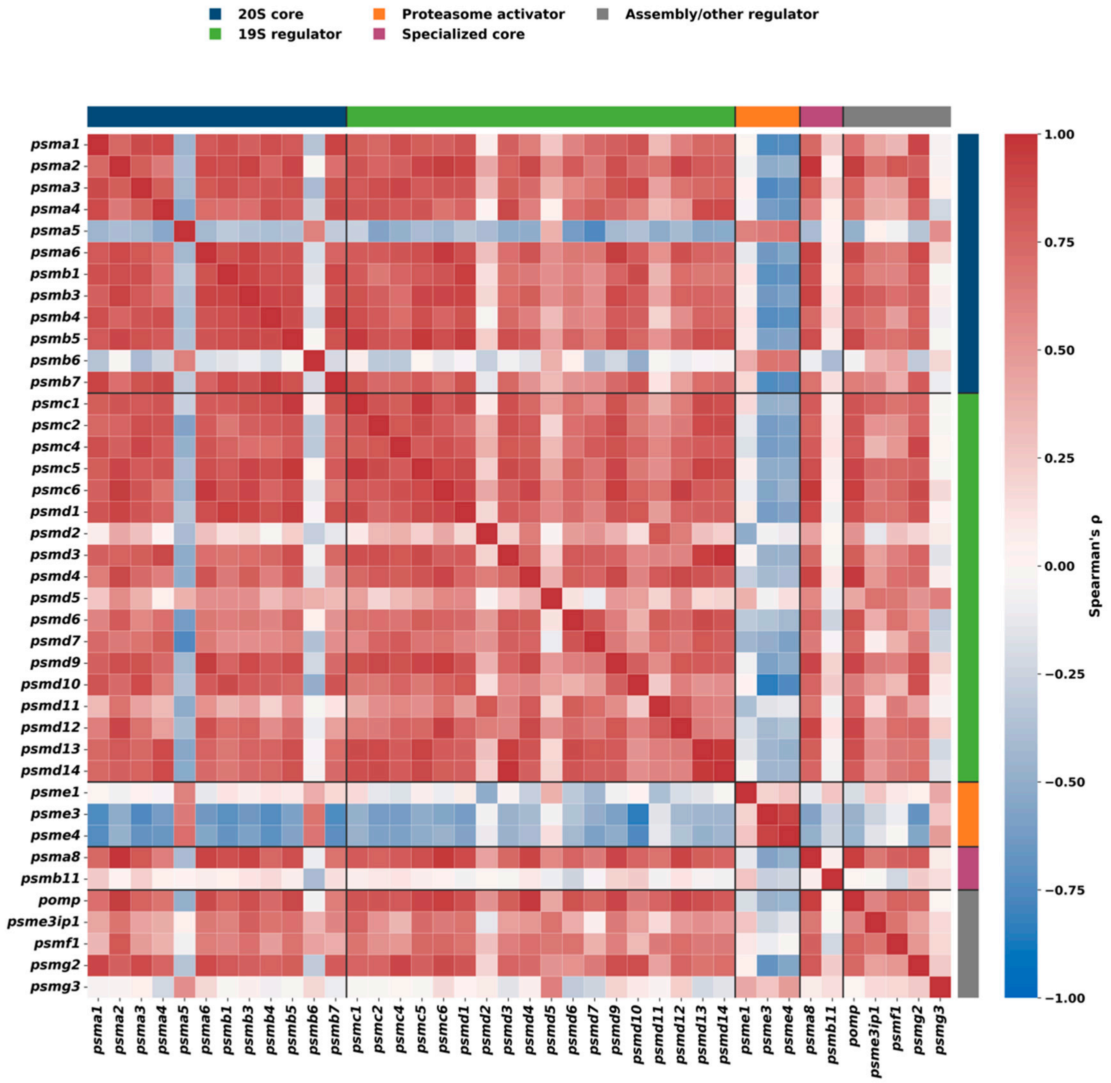

Supplementary Figure S24. Spearman correlation heatmap of UPS subunit expression during European seabass development. Pairwise Spearman correlation coefficients were calculated across the available developmental stages. Where numbered paralogs were present, their TPM values were summed before $\log_2(\text{TPM} + 0.25)$ transformation. Genes with invariant expression profiles were excluded because their correlations could not be calculated. The colour scale ranges from −1 (blue) to +1 (red), while the upper and right annotation bars indicate the functional classification of each subunit. Genes were ordered according to their functional modules, without hierarchical clustering.

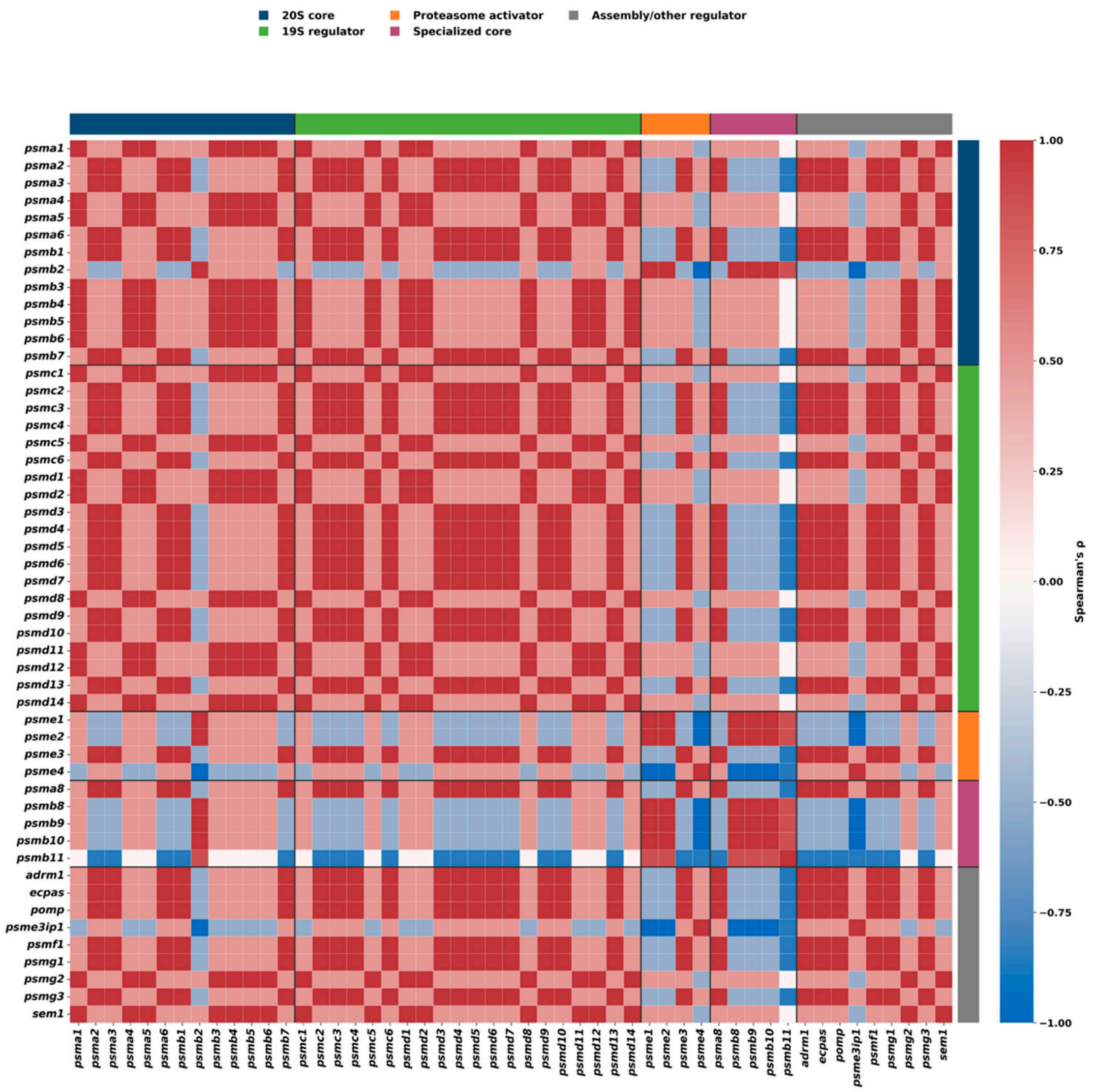

Supplementary Figure S25. Spearman correlation heatmap of UPS subunit expression during gilthead seabream development. Pairwise Spearman correlation coefficients were calculated across the available developmental stages. Where numbered paralogs were present, their TPM values were summed before log_2(TPM + 0.25) transformation. Genes with invariant expression profiles were excluded because their correlations could not be calculated. The colour scale ranges from −1 (blue) to +1 (red), while the upper and right annotation bars indicate the functional classification of each subunit. Genes were ordered according to their functional modules, without hierarchical clustering.

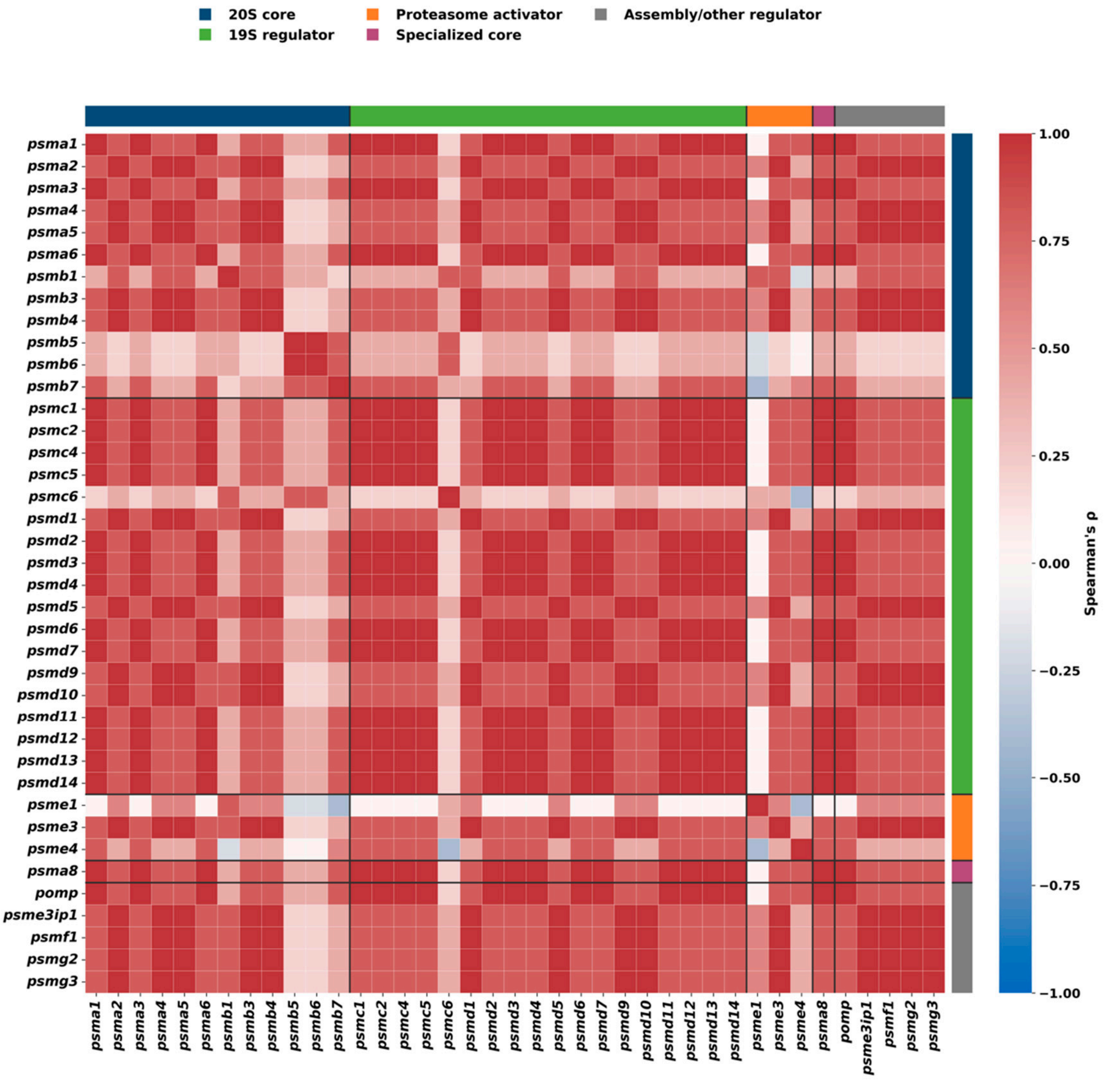

Supplementary Figure S26. Spearman correlation heatmap of UPS subunit expression across European seabass adult tissues. Pairwise Spearman correlation coefficients were calculated between genes across the four adult-tissue profiles. Where numbered paralogs were present, their TPM values were summed before log2(TPM + 0.25) transformation. The matrix is descriptive; no P-value or multiple-testing threshold was applied.

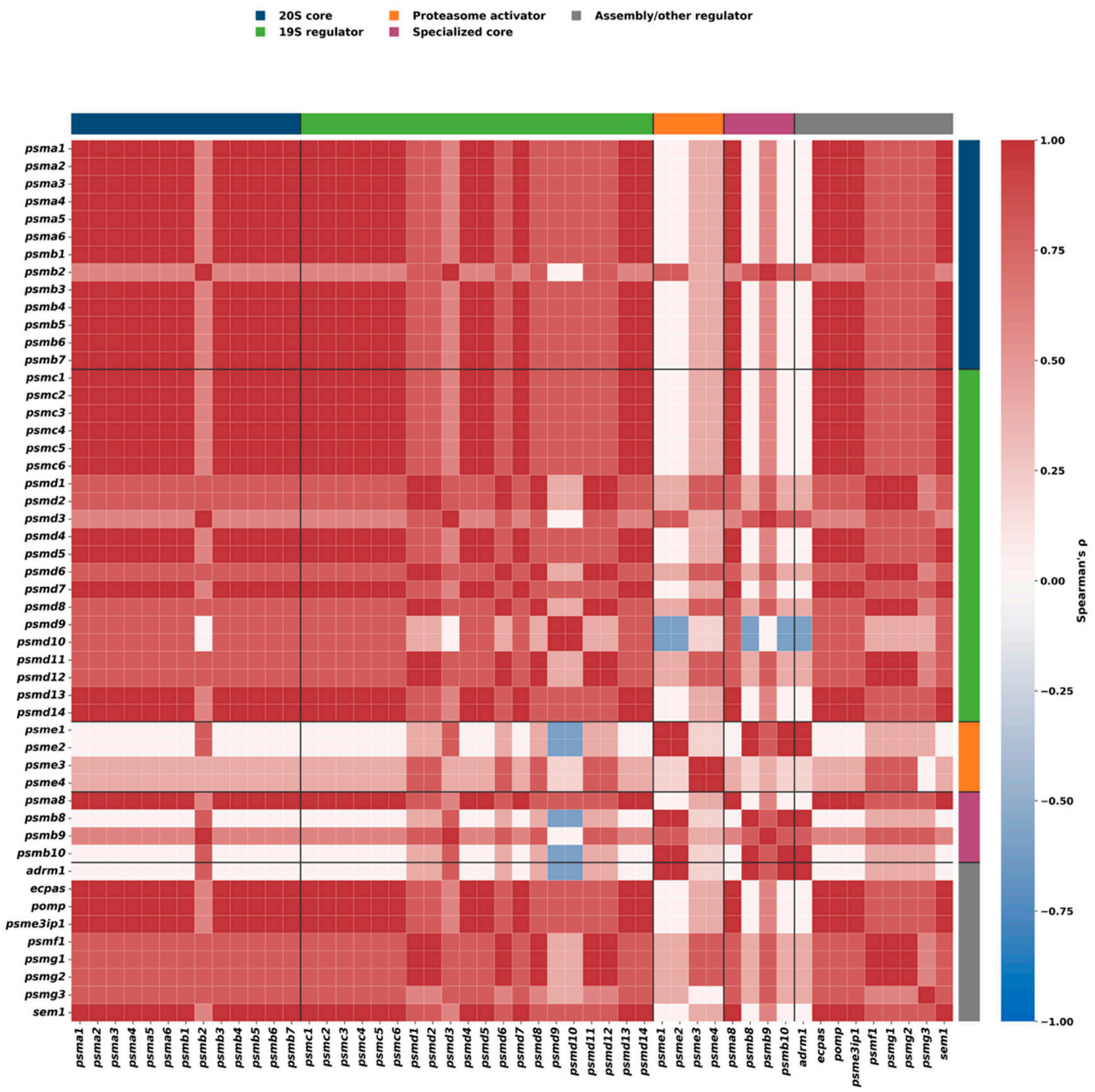

Supplementary Figure S27. Spearman correlation heatmap of UPS subunit expression across gilthead seabream adult tissues. Pairwise Spearman correlation coefficients were calculated between genes across the four adult-tissue profiles. Where numbered paralogs were present, their TPM values were summed before log2(TPM + 0.25) transformation. The matrix is descriptive; no P-value or multiple-testing threshold was applied.
